# Pentacyclic fused diborepinium ions with carbene- and carbone-mediated deep-blue to red emission†

**DOI:** 10.1039/d4sc03835e

**Published:** 2024-08-05

**Authors:** Kimberly K. Hollister, Andrew Molino, VuongVy V. Le, Nula Jones, Wyatt J. Smith, Peter Müller, Diane A. Dickie, David J. D. Wilson, Robert J. Gilliard

**Affiliations:** a Department of Chemistry, Massachusetts Institute of Technology 77 Massachusetts Avenue, Building 18-596 Cambridge MA 02139-4307 USA gilliard@mit.edu; b Department of Chemistry, La Trobe Institute for Molecular Science, La Trobe University Melbourne 3086 Victoria Australia david.wilson@latrobe.edu.au; c Department of Chemistry, University of Virginia Charlottesville Virginia 22904 USA

## Abstract

Designing molecules that can undergo late-stage modifications resulting in specific optical properties is useful for developing structure-function trends in materials, which ultimately advance optoelectronic applications. Herein, we report a series of fused diborepinium ions stabilized by carbene and carbone ligands (diamino-N-heterocyclic carbenes, cyclic(alkyl)(amino) carbenes, carbodicarbenes, and carbodiphosphoranes), including a detailed bonding analysis. These are the first structurally confirmed examples of diborepin dications and we detail how distortions in the core of the pentacyclic fused system impact aromaticity, stability, and their light-emitting properties. Using the same fused diborepin scaffold, coordinating ligands were used to dramatically shift the emission profile, which exhibit colors ranging from blue to red (358–643 nm). Notably, these diborepinium ions access expanded regions of the visible spectrum compared to known examples of borepins, with quantum yields up to 60%. Carbones were determined to be superior stabilizing ligands, resulting in improved stability in the solution and solid states. Density functional theory was used to provide insight into the bonding as well as the specific transitions that result in the observed photophysical properties.

## Abstract 2

Pentacyclic fused diborepinium ions have been synthesized that have carbene- and carbone-mediated deep-blue to red emission profiles.

## Graphical abstract

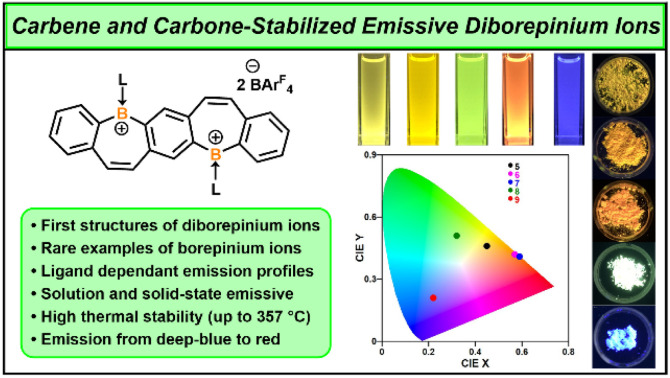

## Introduction

Polycyclic aromatic hydrocarbons (PAHs) have found utility in device technology (*e.g.*, organic light-emitting diodes, photovoltaics, organic field effect transistors) due to their charge transport properties, luminescence, and low cost compared to silicon-based materials.^1^ When designing new luminescent PAH materials with potential for device fabrication and commercial viability, molecules with high thermal stability, color purity, and emission tunability are beneficial.^2^ A common strategy used to modulate emission wavelength is to prepare materials with specific degrees of π-conjugation, where increasing conjugation length tends to lead to a shift toward the red region of the visible spectrum.^1*b*,3^ These efforts are often synthetically arduous, involving challenging multi-step syntheses before the optical properties of a specific material can be tested. Therefore, the development of single-molecule scaffolds that undergo facile late-stage modifications to emit at specified wavelengths is an efficient and economical alternative approach, avoiding the need to synthesize entirely new conjugated materials for each desired optical profile. This can be achieved by the incorporation of boron into the PAH to provide a site for tuning the optoelectronic properties of the material.^1*a*,*c*,4^ The p_*z*_-orbital on boron overlaps with the π-system of PAHs, and therefore, alterations to the structure and bonding around the boron center have a significant impact on the frontier molecular orbitals.^1*c*,3*a*,4^

Borepin is a 7-membered boron-containing ring that is isoelectronic to the tropylium ion. The study of these molecules and their derivatives built on the concept of non-benzenoid aromaticity, introduced by Nozoe^5^ and Dewar^6^ with reports on tropolone, the 7-membered ring ketone.^7^ Since the first aromatic borepin was isolated in 1960,^8^ there have been numerous reports on neutral borepins, with their electronic and optical properties being evaluated.^7*a*^ However, our knowledge of the reactivity, bonding, and optical properties of these systems pales in comparison to 5- and 6-membered boron heterocycles.^1*b*,3*d*,9^ The majority of reported borepins are tricoordinate with an X-type, one-electron-donating ligand at the boron center, typically a sterically demanding aryl substituent that provides kinetic stabilization.^7,10^ This molecular design approach has afforded neutral borepins that can often be handled in air and purified *via* column chromatography. Historically, efforts to tune the electronic and optical properties of borepins have focused on diversifying the X-type ligand on boron,^11^ positing heteroatoms (*e.g.*, S) in rings adjacent to the boracycle,^10*f*,*g*,12^ or embedding the borepin ring into extended conjugated scaffolds.^10*a*–*e*,10*h*,13^ Bonding situations in which the charge- and/or redox-state of borepin is altered are extremely rare, in part due to the need for non-traditional synthetic methodologies to approach these types of compounds.^14^

While there have been recent reports on positively charged boracycles,^15^ cationic borepin chemistry is still in the early stages of development.^14*a*,*b*^ A 2019 report by our laboratory detailed the first borepinium ions, which were stabilized by diamino-N-heterocyclic carbenes (NHCs) and cyclic(alkyl)(amino) carbenes (CAACs).^14*a*^ Jäkle and coworkers then reported monomeric borepinium ions stabilized by NHC ligands,^14*b*^ where the adjacent electron-rich thiophene rings provide stability to the cationic boron center, a strategy often employed in neutral borepins as well.^7*c*,*d*,10*f*,*g*,12,14*b*,16^ These compounds undergo late-stage dimerization reactions to afford diborepinium ions that have enhanced emission as a result of their extended conjugation.^14*b*^ A significant challenge to the isolation of cationic borepins is the preparation of halo-borepin starting materials, which are more reactive than traditional cyclic halo-boranes due to the conformational flexibility of the 7-membered ring, which results in less effective conjugation with the boron p_*z*_-orbital in the boat-shaped conformation.^11*a*^ Consequently, isolated examples of halo-borepins are rare,^14*b*,*d*,17^ compared to more rigid 5- and 6-membered heterocycles.^15*h*,18^ This challenge becomes significantly more pronounced in instances where other heteroatoms are not present in adjacent rings to provide added electronic stabilization.

Tovar and coworkers reported a bis(borepin) scaffold where the borepin units share a common phenyl ring and the boron atoms are stabilized by 1,3,5-tris(*tert*-butyl)phenyl groups.^10*a*,*e*^ Their synthetic pathway made use of an *in situ* generated fused bis(chloro-borepin) to access air- and moisture-stable neutral borepins. We hypothesized that if the bis(chloro-borepin) intermediate could be isolated, it would provide a route to diborepinium ions with enhanced optical properties due to the rigid nature of the pentacyclic fused structure. The majority of neutral borepins emit between 380–500 nm,^7*a*^ with Jäkle's thiophene-linked diborepinium ions emitting in the 446–482 nm range.^14*b*^ Yamaguchi and coworkers reported a neutral planarized B-phenyldibenzo[*b*,*f*]borepin that displayed broad solvatochromic emission spanning from 434–593 nm.^13^ Very recently, Adachi, Ohshita, and coworkers published a reddish phosphorescent neutral borepin stabilized by thiophenes (593–625 nm, solid; 486–493 nm solution).^19^ Achieving emission at these lower energy wavelengths is challenging due to smaller band gaps and aggregation induced quenching effects.^20^

Herein, we report rare examples of borepin cations and the first structurally authenticated examples of diborepinium ions (5–9), providing initial insight into the correlation between bending the pentacyclic core and the photophysical properties. These diborepinium ions are thermally stable up to 163–357 °C, assessed *via* thermogravimetric analysis (TGA) experiments. Notably, compounds 8 and 9 feature two unknown bonding scenarios for borepins, where the boron center accepts both σ- and π-electron density, made possible by the use of carbone ligands as stabilizers. Moreover, compounds 5–9 are among the most blue- and red-shifted borepins that have been reported, spanning from 436–643 nm in solution and 358–600 nm as solids, depending on the electronics of the coordinating ligand. Dramatic shifts in emission are observed between two different carbone ligands, with one emitting in the deep-blue region and the other in the red region, highlighting the potential for late-stage ligand-mediated emission variation. Compounds 5–7 display particularly narrow emission bands, indicating high color purity.^2*a*,*b*^ With the well-established syntheses of diverse carbene ligands,^21^ and the rapidly expanding library of carbone compounds,^22^ this platform provides a unique method to tune the emission range of borepin-containing compounds.

## Results and discussion

**Scheme 1:**
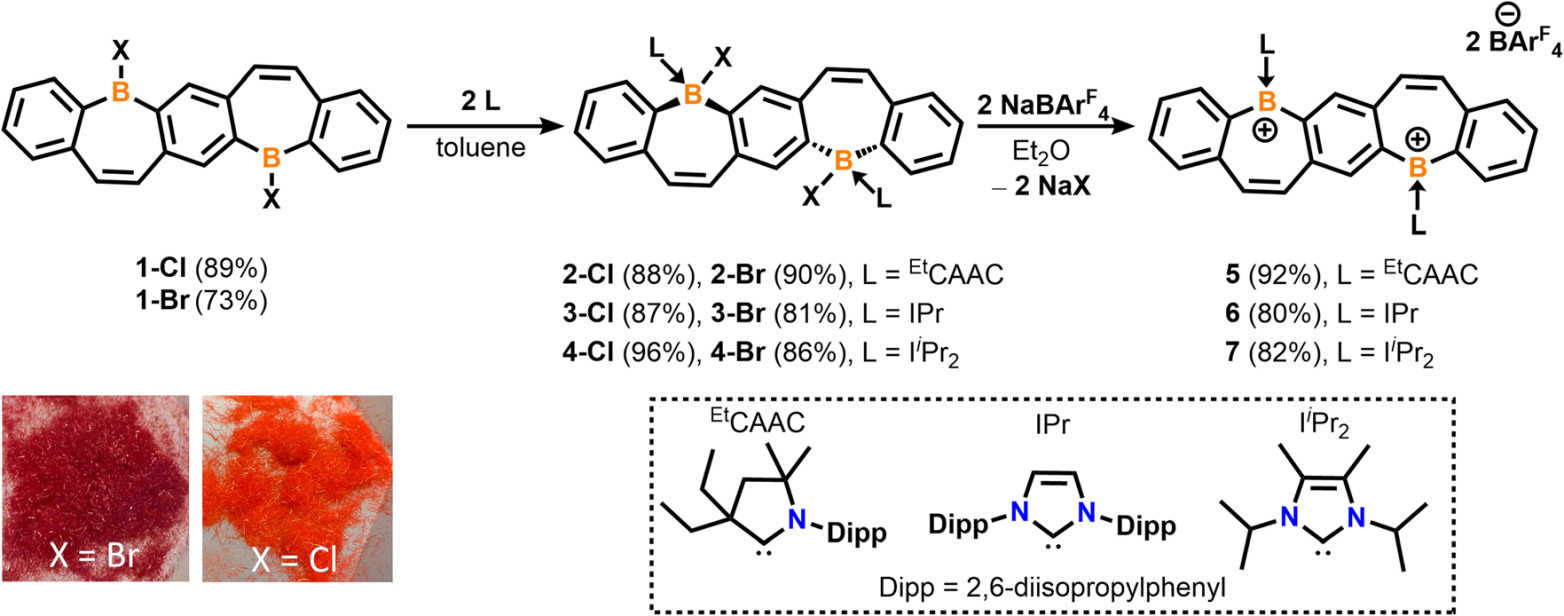
Synthesis of fused bis(Lewis-base stabilized halo-borepin)s 2–4 and dicationic analogues 5–7.

**Fig. 1:**
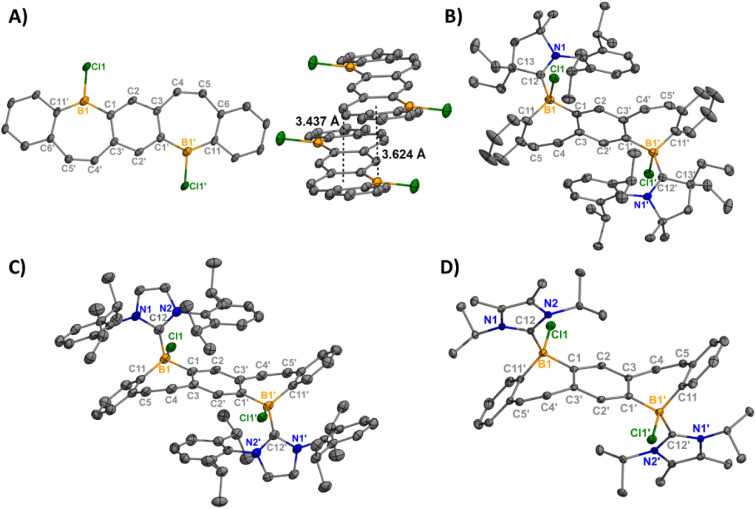
Molecular structures of 1-Cl (left) with displaced π-stacking shown (right) (A), 2-Cl (B), 3-Cl (C), and 4-Cl (D) (thermal ellipsoids at 50% probability; H atoms and co-crystallized solvent omitted for clarity; for 3-Cl, only one of two crystallographically independent molecules shown). Selected bond lengths [Å]: 1-Cl: B1–Cl1 1.800(4), B1–C1 1.561(5), B1–C11′ 1.539(5); 2-Cl: B1–Cl1 1.9669(14), B1–C1 1.615(2), B1–C12 1.6855(18), C12–N1 1.3191(16); 3-Cl: B1–Cl1 1.947(3), B1–C1 1.619(4), B1–C12 1.661(4), C12–N1 1.360(3); 4-Cl: B1–Cl1 1.9341(12), B1–C1 1.6213(16), B1–C12 1.6538(17), C12–N1 1.3558(14). Depictions showing the bend angles in the pentacyclic fused bis(borepin) core are shown in Fig. S60.†

We recently reported the synthesis and isolation of 1-Br*via* a double tin–boron exchange between the reported fused bis(stannacycle)^10*a*^ and boron tribromide (BBr_3_).^14*d*^ Exchanging the borane reagent for BCl_3_, 1-Cl was isolated as an air- and moisture-sensitive bright orange solid in 89% yield (Scheme 1). ^11^B{^1^H} NMR spectroscopy confirms the presence of a tricoordinate halo-borepin with a signal at 58.5 ppm, which is consistent with 1-Br. Orange needle-like single crystals of 1-Cl were grown *via* the slow cooling of a saturated, hot chlorobenzene solution (Fig. 1A). The core of the fused borepin (FBP) is planar and displays parallel displaced π-stacking similar to 1-Br. Interactions between the boron atoms and borepin centroid of an adjacent molecule (3.624(5) Å), as well as the C=C bond of the borepin backbone and an adjacent borepin centroid (3.4367(8) Å), were observed. These intermolecular interactions are slightly shorter than those reported for 1-Br (3.636(3) and 3.4637(6) Å) and may explain the difference in color in the solid-state (1-Br, red; 1-Cl, orange). In a chlorobenzene solution, 1-Br and 1-Cl have nearly identical absorption and emission profiles (Fig. S48†), further supporting that these colorimetric differences are due to solid-state packing effects.

The addition of ^Et^CAAC [2,6-diisopropylphenyl-4,4-diethyl-2,2-dimethyl-pyrrolidin-5-ylidene],^23^ IPr [1,3-bis(2,6-diisopropylphenyl)imidazole-2-ylidene],^24^ or I^i^Pr_2_ [1,3-diisopropyl-4,5-dimethylimidazol-2-ylidene]^25^ to a stirring solution of 1 in toluene resulted in the isolation of compounds 2–4 as white solids in 81–96% yields (Scheme 1). Compounds 2-Cl, 3-Cl, and 4-Cl were characterized by ^1^H, ^13^C, and ^11^B{^1^H} NMR spectroscopy. For the less soluble compounds 2-Cl and 3-Cl, solid-state ^11^B NMR data were obtained, which displayed signals in the range of tetracoordinate boron compounds at −0.8 and −8.4 ppm, respectively. The ^11^B{^1^H} shift of 4-Cl in C_6_D_6_ was observed at −2.5 ppm. The NMR data and structures for bis(Lewis-base stabilized chloro-borepin)s 2–4-Cl are consistent with the bromide analogues, which were discussed in a previous report.^14*d*^ Colorless single crystals of 2-Cl, 3-Cl, and 4-Cl were grown from concentrated THF solutions at −37 °C and exhibited FBP cores that are distorted from planarity (Fig. 1, angles shown in Fig. S60†). All endocyclic and exocyclic boron–carbon bonds are in the range of typical B–C single bonds [1.615(2)–1.6855(18) Å] and the B–Cl bonds [1.9341(12)–1.9669(14) Å] are lengthened compared to 1-Cl [1.800(4) Å].

Upon the addition of sodium tetrakis[3,5-bis(trifluoromethyl)phenyl]borate (NaBAr^F^_4_) to colorless diethyl ether (Et_2_O) solutions of 2–4, the solutions immediately turned bright red (5) or orange (6, 7), signaling the formation of dicationic fused bis(borepins) 5–7. These diborepinium ions were ultimately isolated in 80–92% yields (Scheme 1) and displayed two ^11^B{^1^H} NMR chemical shifts, one corresponding to the tricoordinate positively charged boron (53.4 to 61.2 ppm) and one for the BAr^F^_4_^−^ counteranion (−6.6 to −6.9 ppm). The ^19^F NMR spectra of 5–7 show one peak at −62.8 ppm, corresponding to the F atoms in BAr^F^_4_. Both bromide and chloride precursors were utilized in the halide abstraction reactions with similar success. Although the discussion in this report will focus on the BAr^F^_4_ salts, the utilization of other halide abstraction reagents, such as silver bis(trifluoromethanesulfonyl)imide (AgNTf_2_), also resulted in the formation of diborepinium ions, and their structures are included in the ESI (S5–S7, Fig. S66–68).† Compounds 5–7 were isolated as analytically pure solids based on combustion elemental analysis experiments. However, the ^1^H NMR spectra indicated some protonated ^Et^CAAC (5) or IPr (6) present when redissolved in CD_2_Cl_2_ (details in ESI†). Unfortunately, poor solubility precluded us from utilizing other NMR solvents. Upon further analysis, 5–7 are moderately unstable in halogenated solvents, which is expedited when exposed to ambient light. Decomposition of 7 proceeds the slowest, and therefore suitable NMR data was obtained.

**Fig. 2:**
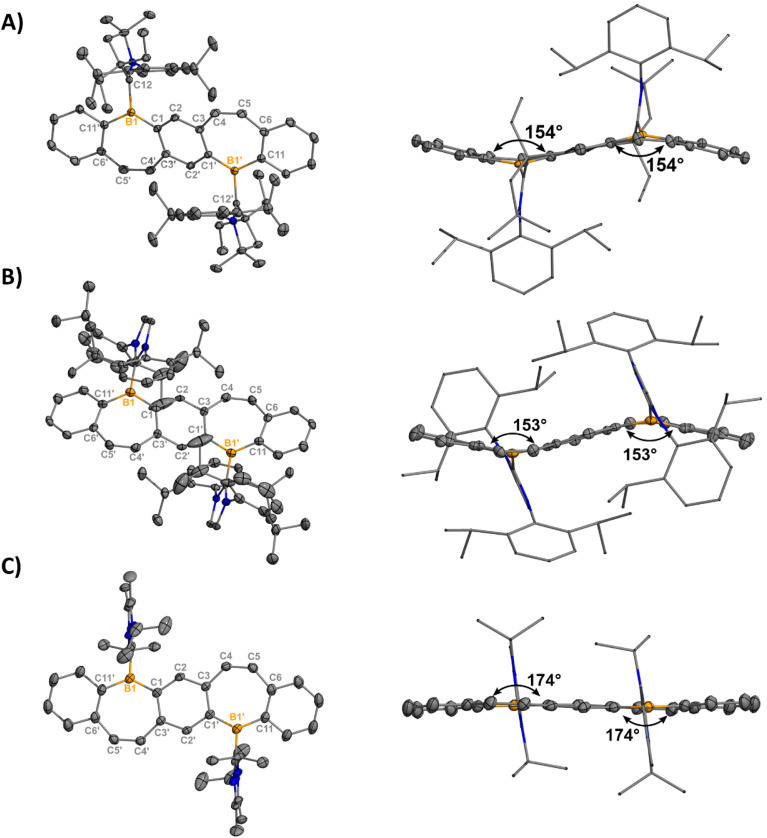
Molecular structures of 5 (A), 6 (B), and 7 (C) with their side views (right) displaying distortion of the FBP core from planarity (thermal ellipsoids are shown at 50% probability; H atoms and co-crystallized solvent were omitted for clarity. Counteranions were omitted for clarity). Angles depicted were measured by drawing planes through the boron atoms and adjacent six-membered rings. Structures shown with anions in Fig. S61†). Selected bond lengths [Å] and angles [°]: 5: B1–C1 1.5535(17), B1–C12 1.6234(17), B1–C11′ 1.5424(17), C12–N1 1.3040(15), C4–C5 1.3487(18), N1–C12–B1–C1 102.14(13); 6: B1–C1 1.547(5), B1–C12 1.617(5), B1–C11 1.525(5), C12–N1 1.362(4), C4–C5 1.341(4), N1–C12–B1–C1 -107.9(4); 7: B1–C1 1.546(2), B1–C12 1.605(2), B1–C11 1.532(2), C12–N1 1.3418(19), C4–C5 1.350(2), N1–C12–B1–C1 96.48(16).

Orange (5, 7) and red (6) single crystals suitable for X-ray diffraction analysis were grown from concentrated solutions of dichloromethane (DCM) in the dark at −37 °C and confirm the halide abstraction from 2–4 (Fig. 2). Notably, these represent the first structurally authenticated diborepinium ions. Charge-separated ion pairs were observed with no contacts between the cationic boron center and BAr^F^_4_ counteranions. Formation of the tricoordinate boron results in increasing planarization of the FBP core compared to the tetracoordinate adducts (Fig. S60†), with 7 being almost fully planar. In 5 and 6, the bulkier ^Et^CAAC and IPr ligands prevented complete planarization of the FBP, and distortion angles of 26° and 27° were measured by drawing planes through the boron atoms and adjacent 6-membered rings. A slight shortening of the ^carbene^C–B1 bond was observed in 5–7 [1.605(2)–1.6234(17)] compared to 2–4 [1.6538(17)–1.6855(18)], although these distances are all consistent with B–C single bonds.^26^ The C4–C5 bonds of the borepin backbone are consistent with C=C double bonds in all three compounds [1.341(4)–1.350(2) Å]. The NHC ligands are orthogonal to the FBP plane with large N1–C12–B1–C1 torsion angles of 102.14(13)° (5), −107.9° (6), and 96.48(16)° (7). In 5, close contacts are observed between the CH_3_ protons of the ethyl groups on CAAC and the cationic boron center (2.4888(14) Å, Fig. S63†). In 7, the isopropyl groups on I^i^Pr_2_ are oriented in opposite directions, with close contacts between the methylene proton and boron (2.3767(17) Å) and the CH_3_ protons and boron (2.4412(16) Å, Fig. S64†).

**Scheme 2:**
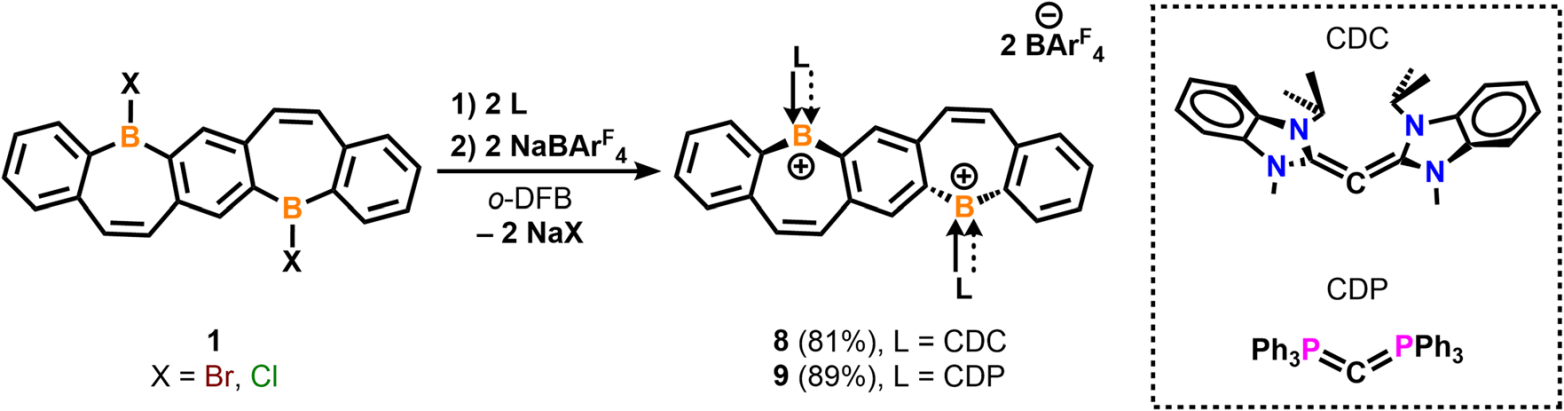
Synthesis of carbone-stabilized diborepinium ions 8 and 9.

Motivated by our recent work utilizing carbone ligands to synthesize air-stable borafluorenium^15*g*^ and azaboraacenium ions,^15*i*^ we sought to understand the impact carbones have on the stability and optical properties of diborepinium ions. Starting from suspensions of bis(halo-borepin) 1 in *o*-difluorobenzene, the carbone species bis(1-isopropyl-3-methyl-benzimidazol-2-ylidene) [CDC]^27^ or hexaphenylcarbodiphosphorane [CDP]^28^ was added, which resulted in dissolution of the solids and an immediate color change to red or yellow, respectively. These color changes were consistent with the nucleophilic displacement of the halide and formation of the dications, resulting from the enhanced σ- and π-donor abilities of the carbones compared to carbenes.^15*g*^ The ^11^B{^1^H} NMR spectra also indicated the formation of the dications, although attempts to crystallize the dications with halide counteranions were unsuccessful. Thus, we employed a one-pot synthesis combining 1, the corresponding carbone, and NaBAr^F^_4_ in *o*-difluorobenzene (*o*-DFB) to form species 8 and 9 in 81 and 89% yields, respectively (Scheme 2). ^1^H NMR experiments indicated clean conversion to the diborepin dications. The ^11^B{^1^H} NMR signals correlated to the tricoordinate cationic boron (55.4 and 59.1 ppm) and tetracoordinate BAr^F^_4_ counteranion (−6.6 and −6.6 ppm), which were consistent with the carbene adducts 5–7. The N–CH_3_ signal in the ^1^H NMR of 8 is broadened, indicating that there may be interactions between the protons and cationic boron center.^15*g*^ The ^19^F NMR spectra of 8 and 9 both exhibited shifts at −62.8 ppm, similar to 5–7. The ^31^P NMR spectrum of compound 9 showed one signal at 24.1 ppm, which is consistent with a reported carbodiphosphorane-difluoroborenium ion (24.8 ppm).^29^ In contrast to the carbene-stabilized diborepin dications, carbone-diborepinium ions 8 and 9 were persistent in solution with no evidence of decomposition after weeks in solution.

**Fig. 3:**
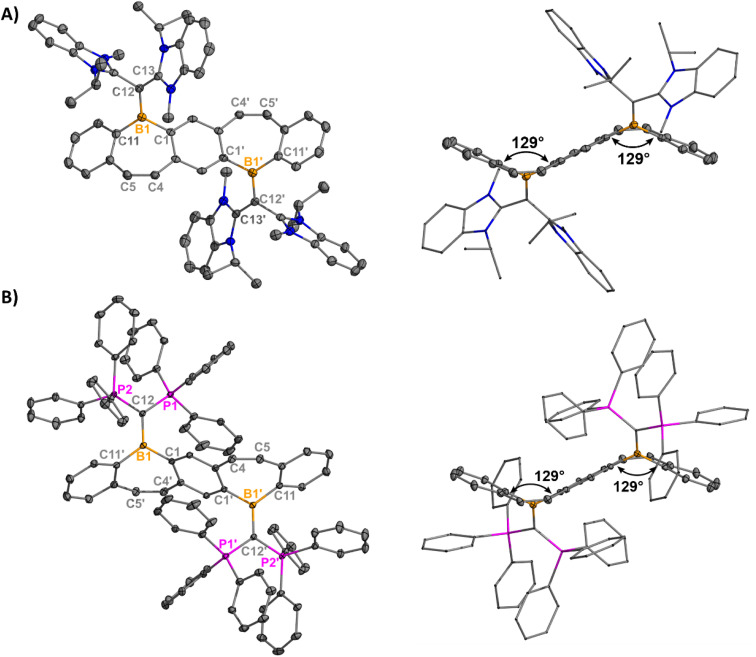
Molecular structures of 8 (A) and 9 (B) with their side views (right) displaying distortion of the FBP core from planarity (thermal ellipsoids are shown at 50% probability; H atoms and co-crystallized solvent were omitted for clarity. Counteranions were omitted for clarity). Angles depicted were measured by drawing planes through the boron atoms and adjacent six-membered rings. Structures shown with anions in Fig. S62†). Selected bond lengths [Å] and angles [°]: 8: B1–C1 1.573(3), B1–C12 1.512(3), B1–C11 1.576(3), C12–C13 1.441(3), C4–C5 1.345(3), C13–C12–B1–C1 −26.8(3); 9: B1–C1′ 1.5688(17), B1–C12 1.5321(18), B1–C11 1.5844(18), C12–P1 1.7610(12), C4–C5 1.3469(18), P1–C12–B1–C1′ 28.79(12).

Yellow plate-like single crystals of 8 were grown from an Et_2_O/hexanes mixture after one week at room temperature, and colorless plate-like crystals of 9 were grown from a DCM/Et_2_O layer at room temperature overnight (Fig. 3). Structural analyses indicated cation formation with no interactions between the BAr^F^_4_ counteranions and borepinium ions. The ^carbone^C–B bonds [8: 1.512(3) Å, 9: 1.5321(18) Å] are shortened compared to the ^carbene^C–B bonds [5: 1.6234(17), 6: 1.617(5), 7: 1.605(2)], which is consistent with the added π-donor ability of the carbone, leading to increased double bond character. Carbodicarbenes are stronger nucleophiles than carbodiphosphoranes,^30^ explaining the slightly shorter bond in 8, compared to 9. The FBP cores are significantly bent out of plane (51° for both), which is much larger than in 5–7 (6–27°). This significant distortion is likely due to the steric bulk of the carbone ligands, and 8 and 9 are stabilized by the extra π-electron donation from the ligand. A short distance was also observed between the N–CH_3_ protons of the CDC and the cationic boron [2.5731(101) Å] in 8 (Fig. S65†). A similar type of intramolecular agostic interaction was previously observed and chemically probed in our CDC-borafluorenium complex [2.657 Å], and the broadening in the ^1^H NMR discussed above supports this interaction persisting in solution.^15*g*^

Thermogravimetric analyses (TGA) were performed to probe the thermal stability of compounds 1-Cl, 1-Br, and 5–9 (Fig. S49–S55†). Bis(halo-borepins) 1-Cl and 1-Br are stable up to 328 and 320 °C respectively. This is quite high for Lewis acidic boracycles and is likely due to the intermolecular π-B(p_*z*_) interactions observed in the solid-state. Remarkably, the majority of the diborepin dications also exhibit exceptional stability. The CAAC- and NHC-diborepinium ions [5, 282 °C; 6, 163 °C; 7, 293 °C] are less thermally stable than the carbone-diborepinium ions [8, 319 °C; 9, 357 °C], consistent with the superior donor capability of carbones. A significant decrease in stability was observed in 6, which also proved to be the least stable in solution. This can be rationalized by the loss of planarity in the FBP core compared to 7 and the weaker donor ability of NHCs compared to CAACs and carbones. Compound 9 displayed the highest thermal stability, likely due to the strength of the CDP ligand and steric protection of the B=C bond by the large PPh_3_ groups.

Compounds 5–9 are stable in the solid-state and can be stored for months under inert conditions. Tests were performed *via* NMR spectroscopy on solid samples of 5–9 to test their stability under air. Compounds 5 and 6 were significantly less stable, with 5 exhibiting over 50% protonation and 6 completely decomposing after three days. Compound 7 displayed very slow protonation of the carbene ligand, containing only 7% protonated I^i^Pr_2_ after three days. Notably, 8 and 9 showed no evidence of decomposition after weeks on the benchtop. These observed trends are consistent with the stability observed in solution and indicate that increased stability can be gained through added electron donation from the ligand (carbones *vs.* carbenes) or through the planarization of the FBP core (7*vs.*5/6).

**Fig. 4:**
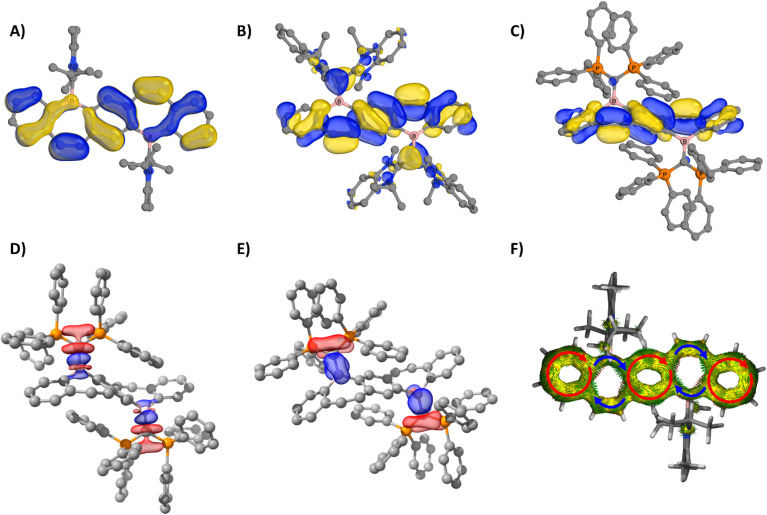
HOMO of 7 (A), 8 (B), and 9 (C) (B3LYP-D3(BJ)/def2-TZVP (CPCM, Et_2_O)). EDA-NOCV deformation densities Δ*ρ*_(1)_ (D) and Δ*ρ*_(2)_ (E) associated with orbital interactions Δ*E*_orb(σ)_ and Δ*E*_orb(π)_ for 9 (charge flow red → blue, isosurface = 0.003). AICD isosurface (0.04) of the π system of 7 (F). Arrows indicate localized diatropic ring currents in each annulated phenyl moiety (red) and conjugated linkages (blue). Boron atoms are depicted in pink, and phosphorus atoms are shown in orange. All compounds are displayed in the same orientation.

The electronic structure of compounds 5–9 were examined using density functional theory. In all compounds, both the highest occupied molecular orbital (HOMO) and the HOMO−1 exhibit π-bonding characteristics across the entire FBP core. For the carbene-stabilized diborepinium ions 5–7, π-bonding in the HOMO is observed across both boron centers (Fig. 4A and S69†). In contrast, for the carbone-stabilized species 8 and 9, the significant planar distortion of the FBP core, induced by the sterically bulky carbone ligands, disrupts π conjugation across the boron centers (Fig. 4B and C). The stability of 8 and 9, even in the absence of stabilization through conjugation within the FBP framework, highlights the essential role of the carbone ligands in stabilizing the borepinium centers. To evaluate the bonding interactions in carbone-stabilized FBPs 8 and 9, energy decomposition analysis combined with the natural orbitals for chemical valence method (EDA-NOCV) was conducted (Table S3†). The ^carbone^C–B bonds in 8 and 9 are predominantly characterized by ^carbone^C→B σ-donating interactions (Δ*E*_orb(σ)_ = −168 to −175 kcal mol^−1^, 63–65%; Fig. 4D, S70 and 71†), with moderate ^carbone^C→B π-donation (Δ*E*_orb(π)_ = −39 to −50 kcal mol^−1^, 15–18%; Fig. 4E, S70 and S71†). While the carbone–borepinium bonding interactions exhibit the same relative orbital contributions, the interaction energies differ significantly. The ^carbone^C–B bonds are stronger in 9 (Δ*E*_int_ = −401.1 kcal mol^−1^) compared to those in 8 (Δ*E*_int_ = −327.0 kcal mol^−1^), largely due to an increased electrostatic interaction in 9 (ΔΔ*E*_elstat_ = 56 kcal mol^−1^). This enhanced electrostatic interaction in 9 is corroborated by the calculated natural atomic partial charges. Specifically, the charges on the ^carbone^C and boron centers are more extreme in 9 (^carbone^C = −1.45*e*, B = +1.02*e*) compared to those in 8 (^carbone^C = −0.70*e*, B = +0.76*e*).

Motivated by the conjugated nature of the frontier molecular orbitals, the aromatic properties of diborepinium ions 5–7 were investigated using nucleus-independent chemical shifts (NICS) and anisotropy of the induced current density (AICD) analysis. According to the Lewis description, the FBP core of diborepinium ions 5–7 is composed of 22 π electrons. In line with Hückel's 4*n* + 2 (*n* = 5) rule, each ring within the FBP framework comprises 6 π electrons and is anticipated to exhibit aromatic character. For reference, an analysis was also conducted on our previously reported monomeric ^IPr^NHC-stabilized borepinium ion^14*a*^ (14 π electrons) to assess the influence of extended annulation on aromaticity. The NICS(1)_ZZ_ value of −27.6 ppm calculated for the annulated phenyl moieties of the monomeric borepinium ion indicates a high degree of aromatic character. Similarly, diborepinium ions 5–7 exhibit significant aromatic character in both the outer and central phenyl moieties, with NICS(1)_ZZ_ values ranging from −26.2 to −28.5 ppm (Table S4†). The planar distortion of the FBP core is therefore determined to have a minimal impact on the local aromaticity of the annulated phenyl rings. The borepinium ring of the monomeric species demonstrates moderate aromatic character, with a calculated NICS(1)_ZZ_ value of −9.2 ppm. The borepinium rings of diborepinium ions 5–7 exhibit only moderate aromatic character, with chemical shift values ranging from −7.4 ppm in the more distorted FBP cores of 5 and 6 to −9.6 ppm in the planar FBP core of 7. AICD analysis identified distinct diatropic ring currents in the annulated phenyl moieties, whereas no circular magnetic currents were detected in the borepinium fragments (Fig. 4F and S72†). Instead, the diborepinium moieties function as conjugated bridges linking the annulated phenyl rings. These findings indicate that the aromaticity identified in the borepinium fragments emerges from annulation rather than electronic delocalization. The combined local and global aromaticity assessments suggest that diborepinium ions 5–7 are most accurately represented as Clar structures, featuring three Clar sextets (Fig. S73†).

**Fig. 5:**
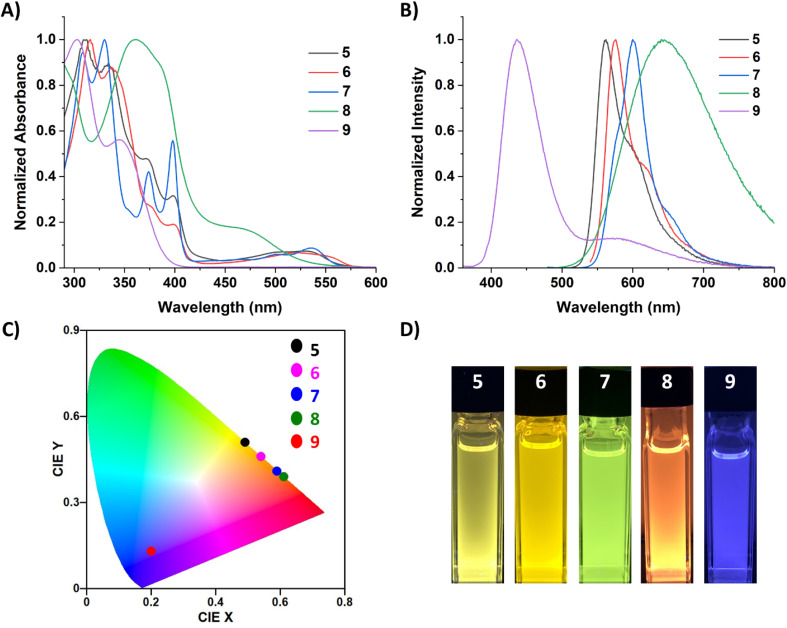
(A) Normalized UV-vis spectra for 5–9 in *ortho*-difluorobenzene (*o*-DFB). (B) Normalized fluorescence spectra for 5–9 in *o*-DFB (*λ*_ex_ = 350 (9), 470 (7, 8), 500 (5), 530 (6) nm; 7–9 = 0.01 mM; 5, 6 = >0.05 mM). (C) CIE (1931) chromaticity diagram of 5–9 in *o*-DFB. (D) Images of cuvettes containing 5–9 in *o*-DFB (*λ*_ex_ = 365 nm).

The photophysical properties of 5–9 were studied *via* UV-vis and fluorescence spectroscopy, quantum yield (QY) determination, and time-controlled single photon counting (TCSPC) measurements. Unfortunately, limited solution-state studies were performed on 5–7 due to their instability in solution at the low concentrations required for QY and lifetime measurements (∼0.001 mM). It should be cautioned that even at higher concentrations, protonated carbene can be observed in the ^1^H NMR spectra of 5 and 6, but dilute solutions of 5–7 gave markedly different UV-vis and fluorescence spectra (Fig. S45–S47†) compared to those displayed in Fig. 5. Compound 7 proved to be more stable than 5 and 6, only decomposing fully at very low concentrations (0.001 mM) and after sitting in solution for a few hours. Alternatively, 8 and 9 persisted in solution even at low concentrations. The UV-vis spectra of 5–9 in *o*-DFB (Fig. 5A) displayed multiple transitions for all five compounds. The fluorescence spectra of 5–9 in *o*-DFB (Fig. 5B) showed significant differences in the emission based on the coordinating ligand, which is more clearly depicted in the International Commission of Illumination (CIE) diagram^31^ (Fig. 5C). The carbene-stabilized species red-shift from 5 < 6 < 7 and display small Stokes shifts (5: 1046.45, 6: 1839.47, 7: 2024.92 cm^−1^) from their weak low energy absorptions (5: 529, 6: 520: 7: 535 nm). Their emission was also quite sharp, with the full width at half maximum (FWHM) being 1703.72 (5), 1245.84 (6), and 1348.95 (7) cm^−1^, although small shoulders are observed at the base of the peak. This is likely due to the planarity/rigidity of the FBP core, leading to minimal structural changes in the excited state. The rigidity of the more planarized compounds leads to fewer vibrational energy levels and lower recombination energies, minimizing the impact of molecular vibrations on the emission profile and therefore having a small FWHM.^2*a*,*b*,32^ Notably, the monomeric borepin cations reported by Jäkle and coworkers display small FWHMs, but the dimerized borepin cations have broad emission profiles with FWHMs larger than 2700 cm^−1^.^14*b*^ The strategy of extending the conjugation while fusing the two borepin rings together in a more rigid scaffold significantly reduces this broad emission profile and produces more efficient emitters. Narrowband emission (<70 nm) is desired for the commercial viability of materials for display applications because it indicates high color purity.^2*a*,*b*^

The carbone-stabilized compounds differ significantly from their carbene counterparts due to the electronic differences of the ligands and the large distortion from planarity in the FBP core. Compound 8 features broad emission in the orange/red region, while 9 shows a pronounced blue-shift relative to the other dications. TD-DFT calculations reveal that the lowest energy transition for carbene-stabilized species 5–7 involve π*←π transitions localized to the FBP core. In contrast, the lowest energy transition in CDC-stabilized diborepinium 8 is characterized by a HOMO to LUMO transition with moderate charge-transfer (CT) character from the CDC to the FBP core. For CDP-diborepinium 9, the experimentally observed lowest energy transition at 360 nm consists of two closely lying electronic transitions: S_1_←S_0_ at 357.4 nm, and S_2_←S_1_ at 349.7 nm. The first excited state (S_1_) involves an FBP-centered π*←π transition from the HOMO to LUMO, whereas the second excited state (S_2_) is characterized by a weakly allowed (*f* = 0.007) HOMO−1 to LUMO CT transition between the CDP ^carbone^C and borepinium centers. Analysis of the relative energies of the molecular orbitals involved in these transitions provides insights into the blue-shifted absorption and emission profiles observed for 9. The LUMO energies in both 8 (−2.88 eV) and 9 (−2.69 eV) are relatively similar. However, the occupied orbitals in CDP-stabilized diborepinium 9 are significantly more stabilized compared to those in CDC-stabilized 8. This stabilization is particularly evident for the HOMO−1 of 9, which exhibits large orbital coefficients on the ^carbone^C–borepinium bond (HOMO−1: 8 (−6.50 eV), 9 (−6.98 eV)) (Fig. S69†). The increased stability of the HOMO and HOMO–1 in 9 is attributed to charge-polarization along the ^carbone^C–borepinium bond, resulting in an enhanced electrostatic interaction. This stabilization leads to a lower energy HOMO−1, contributing to the blue-shifted absorption and emission profiles of 9.

**Fig. 6:**
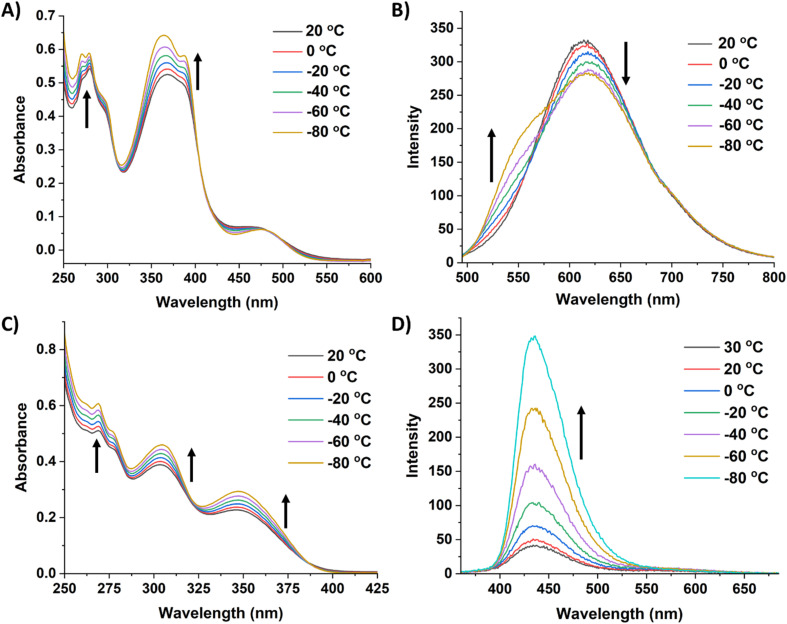
(A) Variable temperature UV-vis spectra of 8 in DCM [0.01 mM]. (B) Variable temperature fluorescence spectra of 8 in DCM (*λ*_ex_ = 480 nm, [0.01 mM]). (C) Variable temperature UV-vis spectra of 9 in DCM [0.01 mM]. (D) Variable temperature fluorescence spectra of 9 in DCM (*λ*_ex_ = 350 nm, [0.01 mM]). The arrows indicate how the features are changing as the temperature decreases.

Due to the robust nature of 8 and 9 in solution, variable temperature (VT) experiments were performed to probe their optical response to temperature changes (Fig. 6). The VT fluorescence spectrum of 8 in DCM displays a decrease in the emission intensity of the peak at 614 nm as temperature decreases, with a small shoulder growing in around 555 nm. This affords a slight change to the overall fluorescence color, moving from red/orange to yellow at lower temperatures (CIE diagram, Fig. S52†). Compound 8 had a QY of 13.4% in DCM at room temperature and 12.2% at −78 °C. In notable contrast, 9 exhibits cryoluminescence, with fluorescence intensity increasing substantially at lower temperatures. Consequently, there was a substantial change in QY from 3.2% at room temperature to 16.4% at −78 °C.

**Fig. 7:**
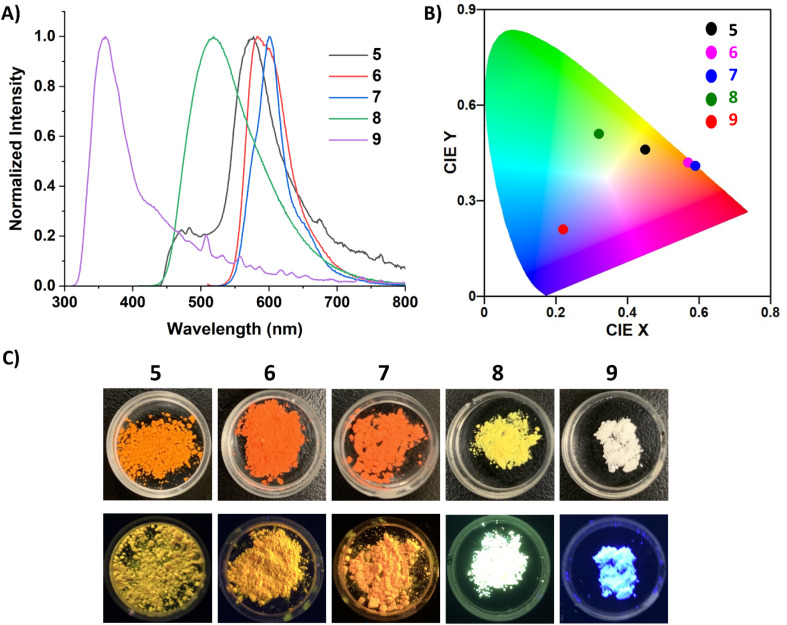
(A) Normalized solid-state fluorescence spectra for 5–9 (*λ*_ex_ = 300 (9), 400 (5, 8), 500 (6, 7) nm). (B) CIE (1931) chromaticity diagram of 5–9 in the solid-state. (C) Images of 5–9 in the solid-state under ambient light (top) and 365 nm UV light irradiation (bottom).

The fluorescence profiles of powder samples of 5–7 were more red-shifted compared to 8 and 9, which is attributed to enhanced conjugation due to the smaller bending angle of their FBP core (Fig. 7A). Therefore 7, which has the most planar solid-state structure (174°), is the furthest red-shifted at 600 nm. Compounds 6 and 7 maintain particularly narrow emission bands in the solid-state, with their FWHM being 1907.72 and 1370.24 cm^−1^, respectively, indicating their high color purity.^2*a*,*b*^ The emission of carbone-stabilized diborepinium ions 8 and 9 are blue-shifted relative to the carbene-diborepinium ions 5–7 and is attributed to the larger distortion from planarity in their solid-state structures. Notably, 8 displays a dramatic hypsochromic shift in the solid-state compared to solution, which is due to the suppression of the charge transfer process that was observed in an *o*-DFB solution. Interestingly, 9 is colorless under ambient light and blue emissive, which is in stark contrast to the yellow (8) and orange (5–7) dications (Fig. 7C).

QY and fluorescence lifetime data were collected on 5–9 in the solution- and solid-state (Table 1). Due to the propensity for 5–7 to form protonated carbene at dilute concentrations necessary for these data, only their solid-state QYs and lifetimes are reported. Compounds 8 and 9 displayed relatively low QYs in an *o*-DFB solution, 9.3 and 4.9%, respectively, although these single digit QYs are comparable to the thiophene-linked borepinium dimers.^14*b*^ Both compounds were fit with a biexponential decay, consistent with the charge transfer processes discussed above, which also helps to explain the moderately low QYs. In the solid-state, 5, 6, and 9 had the lowest QYs, around 9–10%. Compound 7, the most stable of the NHC analogues, displayed a significant increase in solid-state QY, which is attributed to the extended conjugation as a result of the more planar FBP core. Remarkably, 8 had the highest solid-state QY at 60%, which is a significant increase from its solution QY. This type of aggregation-induced emission (AIE) is attributed to the suppression of charge transfer, and therefore, less energy is lost due to non-radiative processes.^33^ Notably, all of the solid-state QYs are higher than reported borepinium ions, with 7 and 8 showing a substantial increase.^14*a*,*b*^

**Table 1:** Quantum yield data and fluorescence lifetimes for 5–9

Cmpd	*Φ*_F_^(*o*-DFB)^	*τ*_s_ b/ns^(*o*-DFB)^	*Φ*_F_^(solid)^	*τ*_s_ b/ns^(solid)^
5	a	a	9.8	4.2
6	a	a	9.1	3.5
7	a	a	44.7	18.5
8	9.3	3.59 (80.9%), 12.47 (19.1%)	60.5	3.2 (89.7%), 14.3 (10.3%)
9	4.9	0.35 (94.4%), 4.70 (5.6%)	10.8	0.78

a Compounds decompose at low concentrations.

b Lifetimes collected at *λ*_ex_ = 337.5 nm.

## Conclusions

We have synthesized and fully characterized five new diborepinium ions stabilized by carbenes and carbones. Notably, these are the first diborepinium ions that have been structurally confirmed, and we detail how alterations to the pentacyclic fused ring system impact their properties. Improved stability of the cationic boron is observed upon the utilization of formally four-electron donating carbone ligands, compared to the two-electron donating carbenes. Thermogravimetric analysis experiments indicated that the dications are thermally stable up to 357 °C, and all above 100 °C which is of interest to researchers who fabricate devices. Fluorescence studies demonstrated that the FBP scaffold is tunable based on the addition of easily synthesized ligands and exhibited emission from 360 to 643 nm. Particularly narrow emission bands were observed in the carbene-stabilized complexes, indicating good color purity. The QYs are also higher than what has been previously reported on borepinium ions, reaching up to 60% in the solid-state. With the library of reported carbenes and carbones dramatically expanding over the past decade, this FBP platform provides an opportunity to design emitters spanning the entire visible region.

## Author contributions

The manuscript was written through contributions of all authors. All authors have given approval to the final version of the manuscript.

## Conflicts of interest

The authors declare no competing financial interest.

## Supplementary Material

SC-015-D4SC03835E-s001

SC-015-D4SC03835E-s002

## Data Availability

Crystallographic data for 1–9 and S5–S7 (CIF) can be found in the Cambridge Crystallographic Database (CCDC: 2344133–2344142, 2344145 and 2344146). All other data have been provided in the ESI,† including NMR spectra, UV-vis and fluorescence spectra, thermogravimetric analysis data, single-crystal X-ray diffraction data, and computational details.
