# Supplementary material for: Pentacyclic fused diborepinium ions with carbene- and carbone-mediated deep-blue to red emission
Source: Chem Sci. 2024 Aug 5;15(35):14358–70. doi: 10.1039/d4sc03835e (PMC11325318; doi:10.1039/d4sc03835e)
Supplement: SC-015-D4SC03835E-s001 [file SC-015-D4SC03835E-s001.pdf]

Supplementary Information (SI) for Chemical Science.
This journal is © The Royal Society of Chemistry 2024

Supporting Information

Pentacyclic Fused Diborepinium Ions with Ligand-Mediated Blue to Red Emission

Kimberly K. Hollister,[a] Andrew Molino,[ab] VuongVy V. Le,[c] Nula Jones,[c] Wyatt J. Smith,[c] Diane A. Dickie,[c] Peter Müller,[a] David J. D. Wilson,[b]* and Robert J. Gilliard, Jr.[a]*

[a] Department of Chemistry, Massachusetts Institute of Technology, 77 Massachusetts Avenue, Building 18-596, Cambridge, MA 02139-4307, United States

[b] Department of Chemistry, La Trobe Institute for Molecular Science, La Trobe University, Melbourne, 3086, Victoria, Australia

[c] Department of Chemistry, University of Virginia, Charlottesville, Virginia 22904, United States

*To whom correspondence should be addressed; david.wilson@latrobe.edu.au, gilliard@mit.edu

Table of Contents **Pages**

General Procedures……………………………………………………………………2-3

Experimental Procedures…………………………………………………………...……...4-12

NMR Data……………………………………………………………………………….....13-55

Photophysical Data………………………………………………………………………...56-58

Thermogravimetric Analysis Data…………………………………………………………59-62

X-ray Crystallography Collection Details…………………………………………..………..63-69

Theoretical Calculations………………………………………………………………….……..70-79

References………………………………………………………………………………80-81

General Procedures: All air- and moisture-sensitive reactions were carried out under an inert atmosphere of argon using standard Schlenk techniques or in a MBRAUN LABmaster glovebox equipped with a -37 °C freezer. Reaction solvents including toluene and hexanes were purified by distillation over sodium (Na) metal. Diethyl ether and tetrahydrofuran (THF) were purified by distillation over Na/benzophenone. Dichloromethane (DCM), o-difluorobenzene (o-DFB), and chlorobenzene were purified via distillation over calcium hydride (CaH_2). Deuterated solvents were purchased from Cambridge Isotope Laboratories and distilled over Na/benzophenone (C_6D_6), Na/K alloy (tol-d_8) and CaH_2 (CD_2Cl_2). All reaction glassware was oven-dried overnight at 190°C. The NMR spectra were collected on Bruker Avance Neo 500mHz, Bruker Advance III 600mHz, Bruker Advance III 800mHz, Varian Inova 500mHz, or Varian 600mHz spectrometers. Proton and carbon signals are reported in ppm and referenced to residual solvent peaks in the deuterated solvent (^{1}H: C_6D_6 δ 7.16, CD_2Cl_2 δ 5.34 $C_6D_5CD_3$ δ 2.08 6.97 7.01 7.09; ^{13}C: CD_2Cl_2 δ 53.84). All solution boron signals are reported in ppm and referenced to BF_3•Et_2O [^{11}B: δ = 0.0] following the standards and procedures established by IUPAC[1] using the unified scale approach. In some cases, the borosilicate probe from the spectrometer can be observed from -20 to 40 ppm when the signal from the compound is too weak and broad to fully suppress the background signal due to similar frequencies. Phosphorus signals were referenced to H_3PO_4 [^{31}P: δ = 0.0] and fluorine signals and were referenced to $CFCl_3$ [^{19}F: δ = 0.0] using the same procedures stated above. All solid-state boron signals are reported in ppm and referenced to $NaBF_4$ [^{11}B: δ = -3.51]. All solid-state carbon signals are reported in ppm and referenced to adamantane. UV-vis data were collected on a Cary 60 UV-vis spectrometer coupled with a Unisoku CoolSpeK cryostat for variable temperature scans. Fluorescence data were collected on an Edinburgh Instruments FS5 spectrofluorometer equipped with a double monochromator for excitation and emission. Variable temperature

fluorescence spectra were collected on a Cary Eclipse fluorescence spectrometer coupled with a Unisoku CoolSpeK cryostat. Samples were prepared in 1 cm square quartz cuvettes. Absolute fluorescence quantum yields were determined using a Hamamatsu C11347-11 Quantaurus-QY Absolute PL Quantum Yield Spectrometer. Samples were prepared inside the glovebox in quartz Petri dishes (solid) or 1 cm square quartz cuvettes with Teflon screw caps (solutions). Solutions were prepared in o-DFB and data were collected with absorbance values below 0.1. Fluorescence lifetimes were recorded using a time-correlated single photon counting (TCSPC) method using an Edinburgh Instruments FS5 spectrofluorometer equipped with a double monochromator for excitation and emission. Measurements were made in the right-angle geometry mode, and the emission was collected through a polarizer set to the magic angle. The excitation source was a 337.5 nm excitation LED lamp. The instrument response function (IRF) was measured for solution samples using a Ludox® solution in deionized water. The quality of all decay fits was judged to be satisfactory, based on the calculated values of the reduced χ^2 (0.8-1.2) and Durbin Watson parameters and visual inspection of the weighted residuals. The following compounds were prepared according to literature procedures: fused bis(stannacycle),[2] **1-Br**,[3] 2,6-(diisopropylphenyl)-4,4-diethyl-2,2-dimethyl-pyrrolidin-5-ylidene [^{Et}CAAC],[4] 1,3-bis-(2,6-diisopropylphenyl)-imidazol-2-ylidene [IPr],[5] 1,3-diisopropyl-4,5-dimethylimidazol-2-ylidene [I^{i}Pr$_2$],[6] **2-Br**,[3] **3-Br**,[3] **4-Br**,[3] bis(1-isopropyl-3-methyl-benzimidazol-2-ylidene)methane [CDC],[7] and hexaphenylcarbodiphosphorane [CDP].[8-9] Sodium tetrakis[3,5-bis(trifluoromethyl)phenyl]borate (NaBAr$^F_4$) was purchased from Ambeed and dried under vacuum with P_2O_5 for one week. All other compounds were purchased from Sigma Aldrich and used as received.

Synthesis of 1-Cl

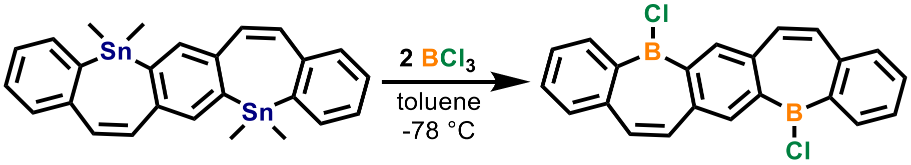

fused bis(stannacycle) **1-Cl**

A solution of fused bis(stannacycle) (0.751 g, 1.30 mmol) in toluene (120 mL) was cooled to -78 °C before BCl_3 (1M in hexanes, 2.87 mL, 2.87 mmol) was added dropwise. The reaction was stirred at -78 °C for 3 hours before warming to room temperature for 30 minutes. The solvent was removed under vacuum and the crude pink solid was washed with hexanes (100 mL). **1-Cl** was isolated as an orange powder (432 mg, 1.16 mmol, 89.2% yield). ^{1}H NMR (600 MHz, CD_2Cl_2) δ= 9.17 (s, 2H), 8.88 (d, 2H), 7.80 (m, 4H), 7.65 (t, 2H), 7.51 (d, 2H), 7.35 (d, 2H). $^{11}B\{^{1}H\}$ NMR (192 MHz, C_6H_5Cl, 100 °C) δ = 58.5. Anal. Calcd. For $C_{22}H_{14}B_2Cl_2$: C, 71.25; H, 3.81. Found: C, 71.35; H, 3.98. *Due to poor solubility no sufficiently resolved ^{13}C NMR could be obtained. Note: **1-Cl** is a strong Lewis acid. Recommended storage is inside the glovebox freezer in a taped vial to prevent potential coordination reactions initiated by solvent vapors in the glovebox atmosphere.

Synthesis of 2-Cl

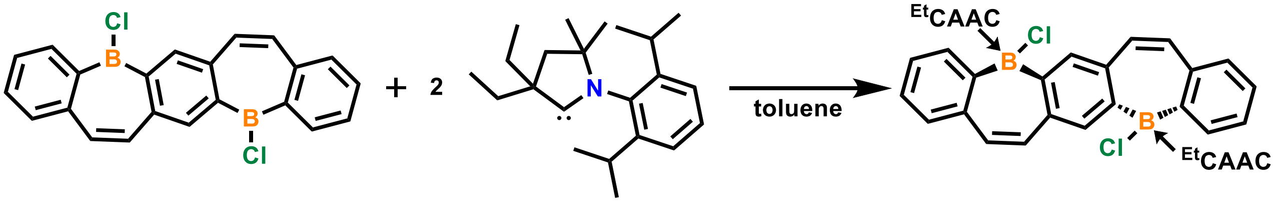

1-Cl **^{Et}CAAC** **2-Cl**

To a suspension of **1-Cl** (95.2 mg, 0.257 mmol) in toluene (4 mL), a solution of 2,6-(diisopropylphenyl)-4,4-diethyl-2,2-dimethyl-pyrrolidin-5-ylidene (**^{Et}CAAC**) (170 mg, 0.539 mmol) in toluene (2 mL) was added dropwise. The reaction was stirred at room temperature for 16 hours before the toluene was removed under vacuum. The crude solid was washed with hexanes

to give **2-Cl** as a light-yellow solid (228 mg, 0.227 mmol, 88.3 % yield). Crystals were grown from concentrated a concentrated THF solution at -37 °C. ^{1}H NMR (600 MHz, Tol-d$_8$, 20 °C) δ 8.78 (d, *J* = 8.5 Hz, 2H), 7.56 (s, 2H), 7.36 (t, *J* = 7.3 Hz, 4H), 7.26 – 7.21 (m, 2H), 6.86 (s, 4H), 6.73 (dd, *J* = 7.8, 1.6 Hz, 2H), 6.07 (d, *J* = 7.7 Hz, 2H), 5.84 – 5.75 (m, 2H), 3.34 (dt, *J* = 11.0, 5.8 Hz, 2H), 2.42 – 2.27 (m, 4H), 1.94 (dq, *J* = 15.3, 7.8 Hz, 2H), 1.87 (d, *J* = 6.2 Hz, 6H), 1.62 (dd, *J* = 14.2, 7.6 Hz, 2H), 1.46 (d, *J* = 7.9 Hz, 6H), 1.30 (s, 6H), 1.20 (d, *J* = 6.5 Hz, 6H), 0.95 (d, *J* = 6.5 Hz, 6H), 0.85 (s, 6H), 0.69 – 0.61 (m, 18H). Solid-state ^{13}C NMR (CP/MAS, 20 kHz) δ 192.18, 144.01, 139.32, 137.76, 135.76, 133.37, 131.92, 129.49, 125.46, 84.73, 57.90, 40.76, 29.56, 28.12, 22.51, 20.64, 11.15. Solid-state ^{11}B NMR (MAS, 20 kHz) δ -0.8. Anal. Calcd. For $C_{66}H_{84}B_2Cl_2N_2$•THF: C, 78.57; H, 8.67; N, 2.62 %. Found: C, 78.62; H, 9.04; N, 2.47 %.

Synthesis of 3-Cl

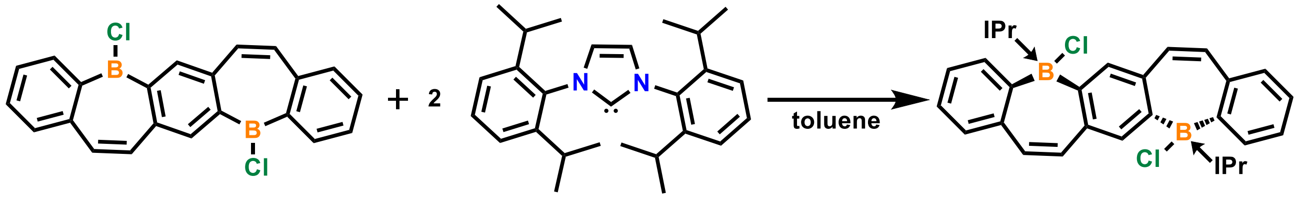

To a suspension of **1-Cl** (36.8 mg, 0.099 mmol) in toluene (4 mL), a solution of 1,3-bis(2,6-diisopropylphenyl)imidazol-2-ylidene (**IPr**) (70.10 mg, 0.180 mmol) in toluene (2 mL) was added dropwise. The reaction was stirred at room temperature for 16 hours before the toluene was removed under vacuum. The crude solid was washed with hexanes to give **3-Cl** as a white solid (65.8 mg, 0.087 mmol, 87.2% yield). ^{1}H NMR (600 MHz, tol-d$_8$, 100 °C) δ 8.13 (br s, 2H), 7.41 (br s, 2H), 7.04 (br s, 3H), 6.90 (br s, 4H), 6.70-6.65 (br m, 8H), 6.46-6.35 (br m, 7H), 6.31 (br s, 4H), 2.95 (br s, 8H, C*H*(CH$_3$)$_2$), 1.47 (br s, 12H, CH(C*H*$_3$)$_2$), 1.35 (br s, 12H, CH(C*H*$_3$)$_2$), 0.80 (br s, 12H, CH(C*H*$_3$)$_2$), 0.74 (br s, 12H, CH(C*H*$_3$)$_2$). *Compound **3-Cl** was not very soluble at room

temperature and the resolution on expected multiplicity was not able to be observed even at higher temperatures. Solid-state ^{13}C NMR (CP/MAS, 20 kHz) δ 170.47, 146.17, 144.30, 140.27, 135.24, 132.93, 130.11, 127.38, 125.89, 124.56, 122.08, 29.41, 28.26, 26.54, 24.23, 23.23, 22.51, 21.36. Solid-state ^{11}B NMR (MAS, 20 kHz) δ -8.4. Anal. Calcd. For $C_{76}H_{86}B_2Cl_2N_4$: C, 79.51; H, 7.55; N, 4.88 %. Found: C, 79.16; H, 7.75; N, 4.73 %.

Synthesis of 4-Cl

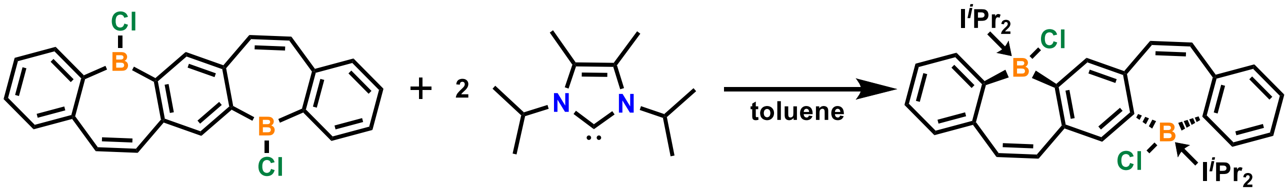

1-Cl **I^{i}Pr$_2$** **4-Cl**

To a suspension of **1-Cl** (22.8 mg, 0.062 mmol) in toluene (2 mL), a solution of 1,3-diisopropyl-4,5-dimethylimidazol-2-ylidene (**I^{i}Pr$_2$**) (22.4 mg, 0.124 mmol) in toluene (2 mL) was added dropwise. The reaction stirred at room temperature overnight before the toluene was removed under vacuum. The crude solid was washed with hexanes to give **4-Cl** as a white solid (32.6 mg, 0.059 mmol, 96.2% yield). ^1H NMR (400 MHz, C_6D_6, 20 °C) δ 8.87 (d, J = 7.6 Hz, 2H), 8.75 (s, 2H), 7.50 (td, J = 7.4, 1.4 Hz, 2H), 7.28 (d, J = 6.3 Hz, 2H), 7.18 (d, J = 7.0 Hz, 2H), 7.15 (d, J = 5.1 Hz, 2H), 6.81 (d, J = 11.7 Hz, 2H), 4.69 (br s, 4H), 1.34 (s, 12H), 0.66 (d, J = 7.0 Hz, 24H). ^{13}C NMR (126 MHz, C_6D_6, 20 °C) δ 137.77, 137.42, 134.01, 131.29, 130.75, 130.15, 128.35, 127.74, 127.38, 124.52, 49.58, 20.74, 10.01. *Due to coupling with quadrupolar nulcei, the carbon atoms adjacent to boron could not be resolved. ^{11}B NMR (192 MHz, C_6D_6, 20 °C) δ -2.5.

Synthesis of 5

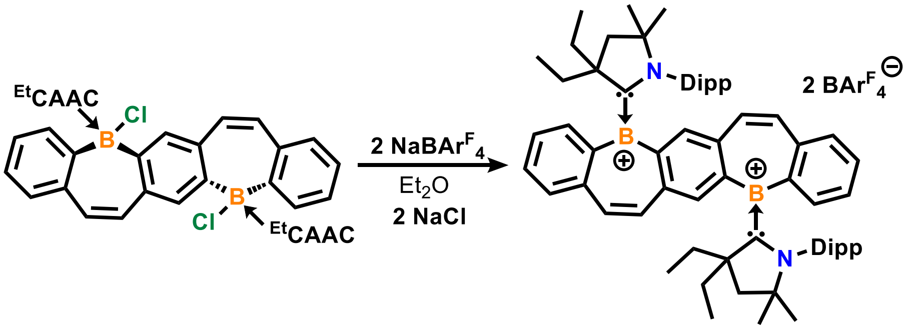

2-Cl **5**

To a suspension of **2-Cl** (77.8 mg, 0.0780 mmol) in Et_2O (3 mL), $NaBAr^F_4$ (145 mg, 0.164 mmoL) was added in one portion. A color change from white to clear red was observed. The reaction was stirred for 3 hours at room temperature before passing through a 0.45 µm PTFE syringe filter to remove fine white NaCl powder and dried. The sticky solid was triturated with hexanes (2 x 2 mL) to isolate **5** as a red powder (190 mg, 0.0716 mmol, 91.8 % yield). *The reaction also proceeds with **2-Br**. Crystals were grown from a concentrated DCM solution at -37 °C. ^1H NMR (500 MHz, CD_2Cl_2, 20 °C) δ 8.17 (br s, 1H), 8.01 (d, J = 7.9 Hz, 1H), 7.94 (t, J = 7.6 Hz, 1H), 7.88 (d, J = 7.0 Hz, 1H), 7.84 – 7.68 (m, 21H), 7.63 (ddd, J = 8.1, 5.4, 2.9 Hz, 2H), 7.55 (s, 8H), 7.47 (d, J = 12.5 Hz, 1H), 7.41 (q, J = 7.8 Hz, 2H), 7.35 (d, J = 12.3 Hz, 1H), 7.21 (d, J = 7.5 Hz, 2H), 7.14 (d, J = 7.3 Hz, 1H), 7.06 (d, J = 7.6 Hz, 1H), 6.83 (s, 1H), 2.94 (s, 1H), 2.82 – 2.70 (m, 1H), 2.70 – 2.55 (m, 4H), 2.47 – 2.35 (m, 1H), 2.34 – 2.25 (m, 1H), 2.10 (h, J = 7.4 Hz, 1H), 1.99 – 1.85 (m, 1H), 1.76 – 1.60 (m, 11H), 1.55 (s, 4H), 1.40 (d, J = 6.5 Hz, 4H), 1.29 (d, J = 6.5 Hz, 4H), 1.20 – 1.07 (m, 10H), 0.87 (t, J = 7.3 Hz, 4H), 0.81 (q, J = 7.5 Hz, 6H), 0.67 (t, J = 7.4 Hz, 4H), 0.23 (s, 3H), -0.27 (d, J = 6.6 Hz, 3H), -0.36 (s, 1H). ^{13}C NMR (126 MHz, CD_2Cl_2) δ 162.15 (dd, J = 99.5, 49.9 Hz, BAr^F_4 CF_3), 145.36 (d, J = 24.8 Hz), 144.97, 144.91, 136.65, 136.39, 135.21, 134.58, 133.04, 132.69, 132.09, 131.14, 129.72 – 129.59 (m), 129.40 (dd, J = 5.8, 3.0 Hz), 129.15 (dd, J = 5.4, 2.6

Hz), 128.90 (dd, J = 5.7, 3.0 Hz), 128.65 (d, J = 15.2 Hz), 128.25, 127.93 (d, J = 18.8 Hz), 127.64, 127.44, 126.08, 123.91, 121.74, 117.89, 86.62, 86.13, 66.23, 66.19, 65.77, 31.06, 30.93, 30.69, 30.53, 30.44, 30.26, 30.19, 29.39, 27.38, 26.53, 25.86, 25.27, 24.98, 15.44, 10.38, 10.30. ^{11}B NMR (160 MHz, CD_2Cl_2) δ 59.7, -6.6. ^{19}F NMR (471 MHz, CD_2Cl_2, 20 °C) -62.8. Anal. Calcd. For $C_{130}H_{108}B_4F_{48}N_2$: C, 58.84; H, 4.10; N, 1.06 %. Found: C, 58.80; H, 4.04; N, 1.04 %.

Synthesis of 6

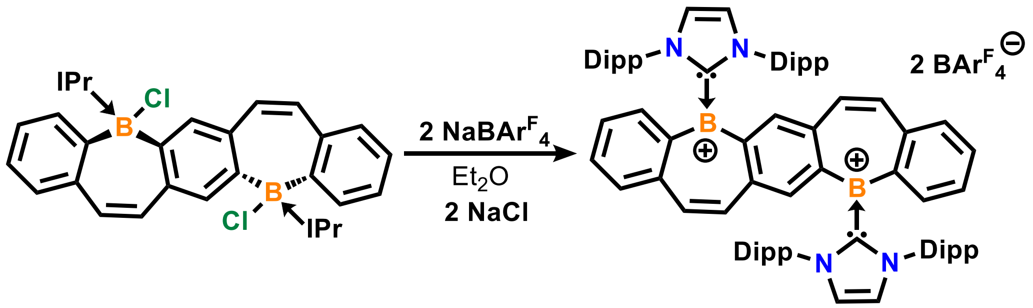

3-Cl **6**

To a suspension of **3-Cl** (64.4 mg, 0.0561 mmol) in Et_2O (2 mL), NaBAr$^F_4$ (105 mg, 0.119 mmol) was added in one portion. A color change from white to an orange suspension was observed. The reaction was stirred for 3 hours at room temperature before passing through a 0.45 µm PTFE syringe filter and removing the solvent. The sticky solid was triturated with hexanes (2 x 3 mL) to isolate **6** as a bright orange solid (125 mg, 0.0446 mmol, 79.5% yield). Crystals were grown from a concentrated DCM solution at -37 °C. ^{1}H NMR (500 MHz, CD_2Cl_2, 20 °C) δ 7.81 (td, J = 7.5, 1.5 Hz, 2H, FBP CH), 7.78 (s, 4H, IPr CH), 7.74 – 7.70 (m, 20H, BAr$^F_4$ CH/IPr CH), 7.55 (s, 8H, BAr$^F_4$ CH), 7.47 (t, J = 8.0 Hz, 6H), 7.40 (t, J = 7.5 Hz, 2H, FBP CH), 7.27 (d, J = 12.6 Hz, 2H, FBP CH=CH), 7.15 (d, J = 7.8 Hz, 8H, IPr CH), 6.86 (d, J = 12.4 Hz, 2H, FBP CH=CH), 2.52 (hept, J = 6.6 Hz, 8H, IPr CH(CH_3)$_2$), 1.09 (d, J = 6.7 Hz, 24H, IPr CH(CH_3)$_2$), 0.46 (d, J = 6.7 Hz, 24H, IPr CH(CH_3)$_2$). ^{13}C NMR (101 MHz, CD_2Cl_2) δ 162.15 (dd, J = 99.9, 49.8 Hz, BAr$^F_4$ CF$_3$), 145.28, 145.17, 144.30, 140.44, 140.11, 138.58, 136.39, 135.94, 135.21, 133.52, 133.45,

133.24, 132.13, 131.51, 129.40, 129.06, 128.01, 126.35, 126.10, 125.70, 123.65, 117.88, 29.88 (IPr *C*H(CH3)2), 26.41 (IPr CH(*C*H3)2), 21.94 (IPr CH(*C*H3)2). $^{11}B\{^1H\}$ NMR (192 MHz, CD_2Cl_2, 20 °C) δ = 61.2, -6.7. ^{19}F NMR (471 MHz, CD_2Cl_2, 20 °C) -62.8. Anal. Calcd. For $C_{140}H_{110}B_4F_{48}N_4$: C, 59.98; H, 3.95; N, 2.00 %. Found: C, 59.57; H, 3.82; N, 1.79 %.

Synthesis of 7

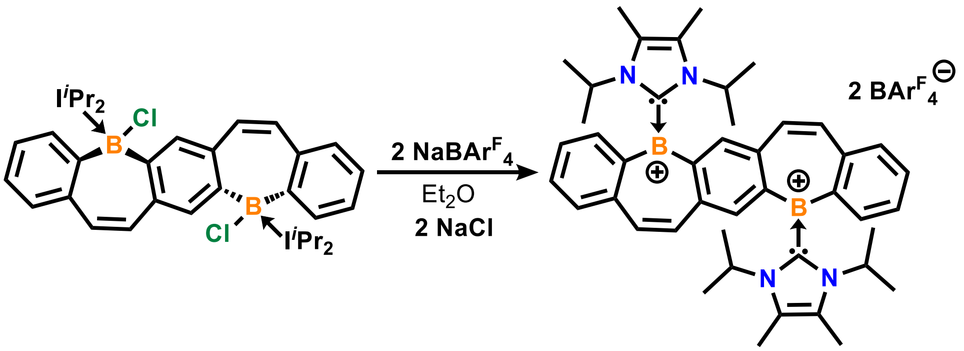

4-Cl 7

To a suspension of **4-Cl** (68.8 g, 0.0920 mmol) in Et_2O (3 mL), $NaBAr^F_4$ (171 mg, 0.193) was added in one portion. A color change from white to an orange suspension was observed. The reaction was stirred for 3 hours at room temperature before filtering and washing the solid with Et_2O (2 x 5 mL) and hexanes (2 x 5 mL). A bright orange solid was isolated (180 mg, 0.075 mmol, 82% yield). Crystals were grown from a concentrated DCM solution at -37 °C. ^1H NMR (500 MHz, CD_2Cl_2, 20 °C) δ 8.18 (s, 2H, FBP C*H*), 8.08 (td, *J* = 7.6, 1.4 Hz, 2H, FBP C*H*), 8.01 (dd, *J* = 8.0, 0.9 Hz, 2H, FBP C*H*), 7.75 (td, *J* = 7.7, 1.4 Hz, 2H, FBP C*H*), 7.72 – 7.69 (m, 16H, BAr^F_4 C*H*), 7.61 – 7.56 (m, 6H, FBP C*H*), 7.53 (s, 8H, BAr^F_4 C*H*), 4.21 (hept, *J* = 6.8 Hz, 4H, I^iPr_2 C*H*(CH3)2), 2.53 (s, 12H, C*H*3), 1.28 (dd, *J* = 6.9, 4.7 Hz, 24H, CH(C*H*3)2). ^{13}C NMR (126 MHz, CD_2Cl_2) δ 162.13 (dd, *J* = 100.0, 49.9 Hz, BAr^F_4 *C*F3)., 147.39, 146.41, 142.84, 141.78, 138.14, 137.22, 135.65, 135.19, 134.12, 129.60, 129.39, 129.14, 128.22, 126.05, 123.89, 121.72, 119.23, 117.88, 66.17, 54.78 (I^iPr_2 *C*H(CH3)2), 21.99 (d, *J* = 13.3 Hz, I^iPr_2 CH(*C*H3)2), 10.69 (I^iPr_2 *C*H3).

^{11}B NMR (161 MHz, CD_2Cl_2, 20 °C) δ 53.4, -6.9. ^{19}F NMR (471 MHz, CD_2Cl_2, 20 °C) δ -62.9. Anal. Calcd. For $C_{108}H_{78}B_4F_{48}N_4•2CH_2Cl_2$: C, 51.67; H, 3.23; N, 2.19 %. Found: C, 51.74; H, 3.06; N, 2.19 %.

Synthesis of 8

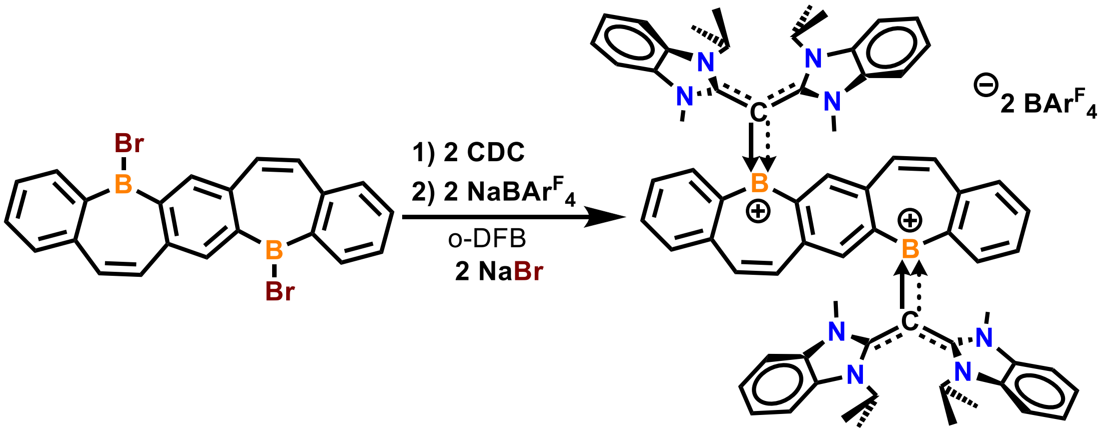

To a suspension of **1-Br** (60.1 mg, 0.131 mmol) in o-difluorobenzene, bis(1-isopropyl-3-methyl-benzimidazol-2-ylidene)methane [CDC] (99 mg, 0.28 mmol) was added in one portion. The reaction immediately turned to a red suspension. After 5 minutes, NaBAr$^F_4$ (243 mg, 0.275 mmol) was added in one portion. The reaction was stirred for 17 hours before passing through a 0.45 µm PTFE syringe filter and removing the solvent. The sticky solid was triturated with hexanes (3 x 3 mL) and **8** was isolated as a red solid (292 mg, 0.106 mmol, 81.1% yield). ^1H NMR (500 MHz, CD_2Cl_2, 20 °C) δ 7.72 (t, J = 2.8 Hz, 16H, BAr$^F_4$ C*H*), 7.60 – 7.39 (m, 28H, BAr$^F_4$ C*H*/FBP C*H*/CDC C*H*), 7.39 – 7.29 (m, 4H, FBP C*H*), 7.09 (td, J = 7.4, 1.3 Hz, 2H, FBP C*H*), 6.87 (d, J = 12.5 Hz, 2H, FBP C*H*=C*H*), 6.53 (d, J = 13.1 Hz, 2H, FBP C*H*=C*H*), 4.30 (p, J = 7.0 Hz, 4H, CDC C*H*($CH_3)_2$), 3.72 (br s, 12H, CDC C*H*$_3$), 1.05 (br s, 24H, CDC CH(C*H*$_3)_2$). ^{13}C NMR (126 MHz, CD_2Cl_2) δ 162.14 (dd, J = 99.5, 49.9 Hz, BAr$^F_4$ *C*F_3), 157.52, 135.20, 133.91, 132.73, 131.90, 131.68, 130.49, 129.23, 128.40, 126.37, 126.06, 123.90, 121.73, 117.88, 117.84, 114.09,

112.04, 52.17 (CDC $CH(CH_3)_2$), 33.85 (CDC CH_3), 20.17 (CDC $CH(CH_3)_2$). ^{11}B NMR (161 MHz, CD_2Cl_2, 20 °C) δ 55.4, -6.6. ^{19}F NMR (471 MHz, CD_2Cl_2, 20 °C) δ -62.8. Anal. Calcd. for $C_{132}H_{94}B_4F_{48}N_8$: C, 57.71; H, 3.45; N, 4.08 %. Found: C, 57.52; H, 4.03; N, 4.00.

Synthesis of 9

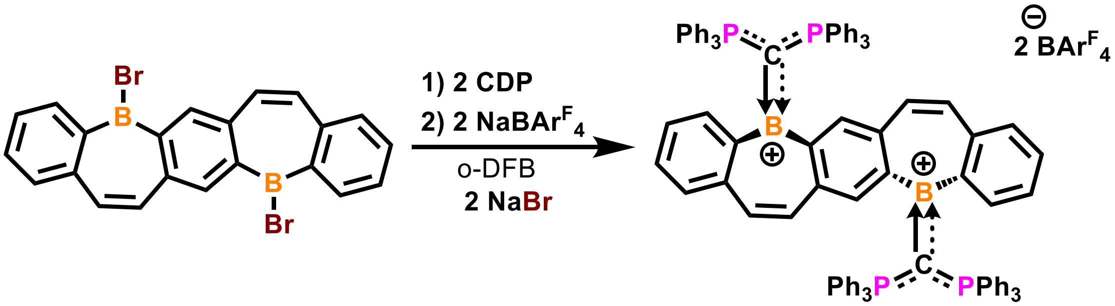

1-Br **9**

To a suspension of **1-Br** (67.6 mg, 0.147 mmol) in o-difluorobenzene, hexaphenylcarbodiphosphorane [CDP] (166 mg, 0.309 mmol) was added in one portion. Reaction immediately turned cloudy yellow. After five minutes, $NaBAr^F_4$ (274 mg, 0.309 mmol) was added in one portion. Reaction was stirred for 17 hours before passing through a 0.45 µm PTFE syringe filter and removing the solvent. Sticky solid was triturated with hexanes (3 x 3 mL). Pale yellow solid isolated (407 mg, 0.131 mmol, 89.3% yield). Product can be further purified by suspending in diethyl ether and putting in freezer before filtering cold. ^{1}H NMR (500 MHz, CD_2Cl_2, 20 °C) δ 7.76 (t, J = 2.4 Hz, 16H, BAr^F_4 CH), 7.57 (s, 8H, BAr^F_4 CH), 7.22 (br s, 38H, PPh_3 CH)*, 7.11 (d, J = 7.4 Hz, 2H, FBP CH)*, 7.04 (br s, 22H, FBP CH)*, 6.89 (td, J = 7.4, 1.4 Hz, 2H, FBP CH), 6.84 (td, J = 7.4, 1.3 Hz, 2H, FBP CH), 6.75 (d, J = 7.6 Hz, 2H, FBP CH), 6.41 (s, 2H. FBP CH), 6.29 (d, J = 12.2 Hz, 2H, FBP CH=CH), 5.98 (d, J = 12.0 Hz, 2H, FBP CH=CH). *Peaks at 7.22, 7.11 and 7.04 overlap but total to 62 protons. ^{13}C NMR (126 MHz, CD_2Cl_2, 20 °C) δ 162.19 (dd, J = 99.7, 49.6 Hz, BAr^F_4 CF_3), 135.90, 135.23 (BAr^F_4 CH), 134.23 – 133.83 (m, PPh_3 CH), 133.12, 133.01, 131.96 (FBP CH=CH), 130.46 (FBP CH=CH), 129.69 (dd, J = 5.5, 2.8 Hz), 129.44 (dd, J

= 5.5, 2.6 Hz), 129.26 – 128.98 (m, PPh_3 *C*), 128.94 (dd, *J* = 6.1, 2.8 Hz), 128.27, 128.14, 127.94, 127.53, 127.10, 126.99, 126.10, 125.32, 123.94, 121.77, 117.91 (p, *J* = 4.1 Hz). ^{11}B NMR (161 MHz, CD_2Cl_2, 20 °C) δ 59.1, -6.6. ^{31}P NMR (203 MHz, CD_2Cl_2, 20 °C) δ 24.1. ^{19}F NMR (471 MHz, CD_2Cl_2, 20 °C) δ -62.8. Anal. Calcd. for $C_{160}H_{98}B_4F_{48}P_4$: C, 62.00; H, 3.19 %. Found: C, 62.58; H, 3.21.

NMR data

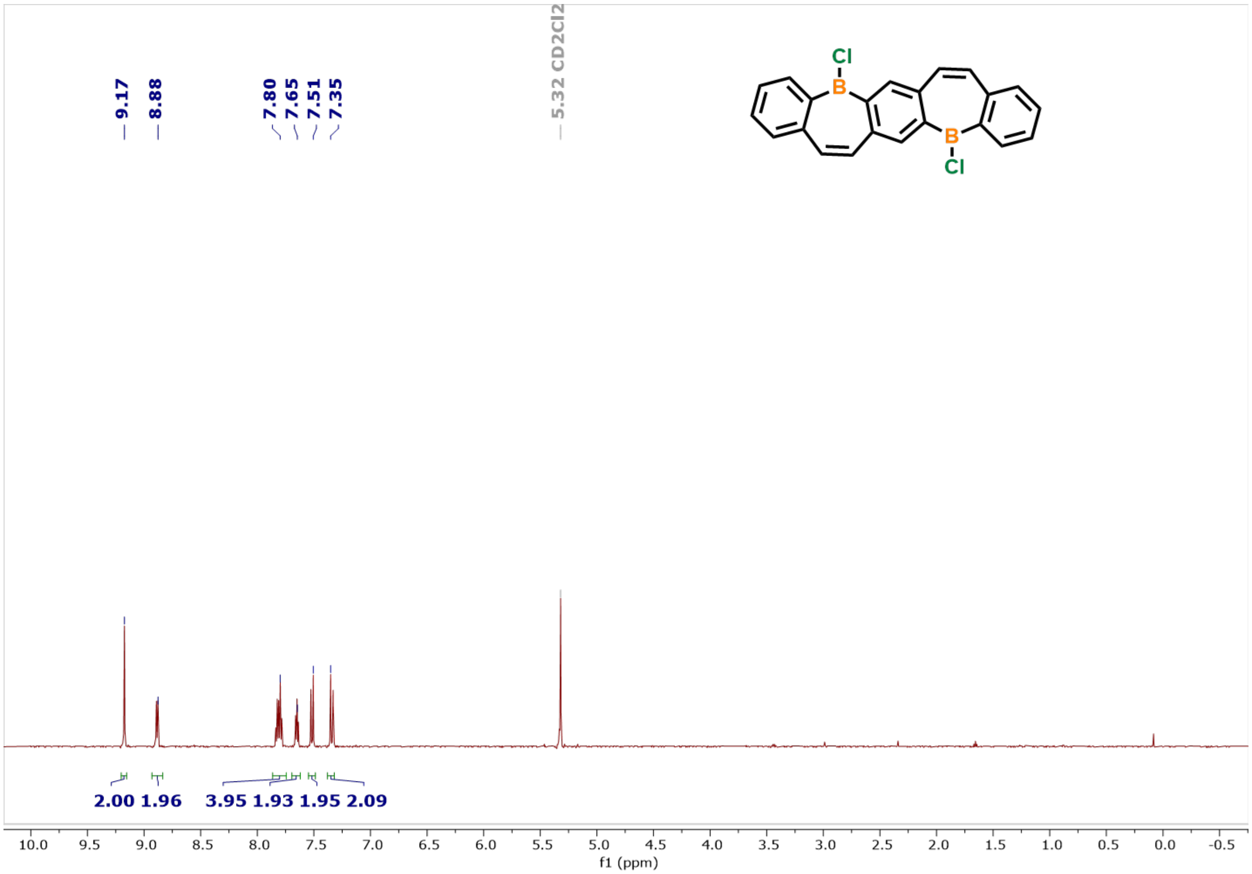

Figure S1. ^{1}H NMR (600 MHz, CD_2Cl_2, 20 °C) spectrum of **1-Cl**.

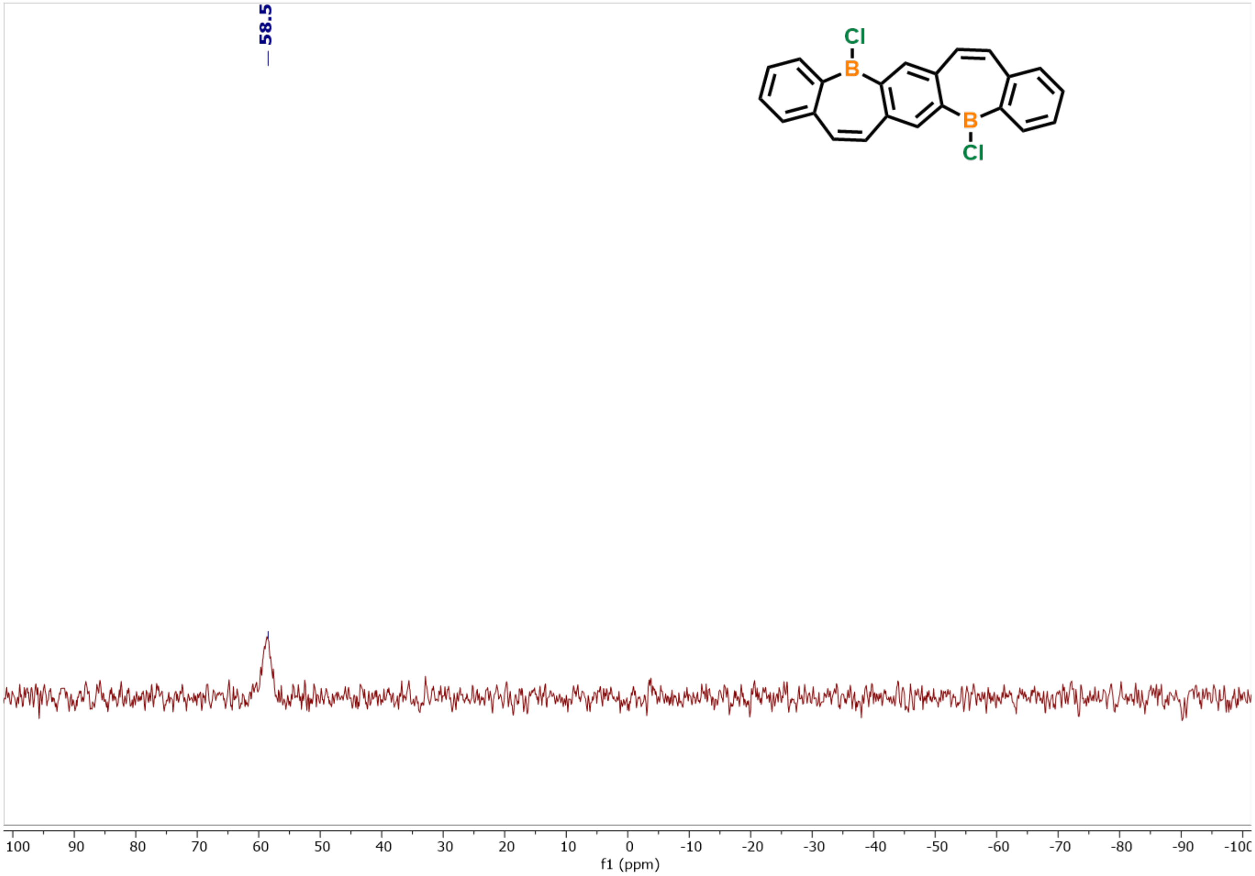

Figure S2. $^{11}B\{^{1}H\}$ NMR (192 MHz, C_6H_5Cl, 100 °C) spectrum of **1-Cl**.

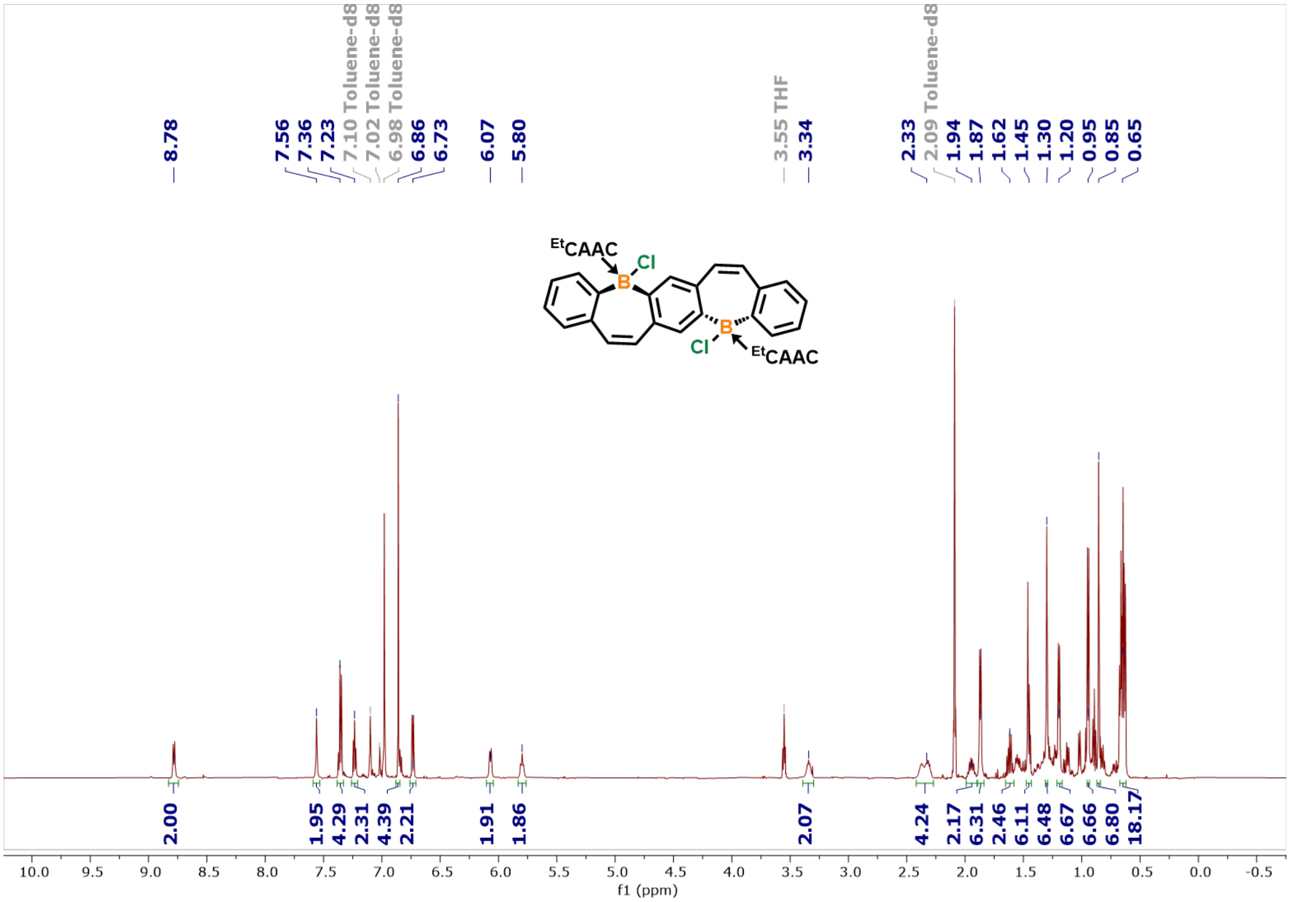

Figure S3. ^{1}H NMR (600 MHz, toluene-d_8, 20 °C) spectrum of **2-Cl**.

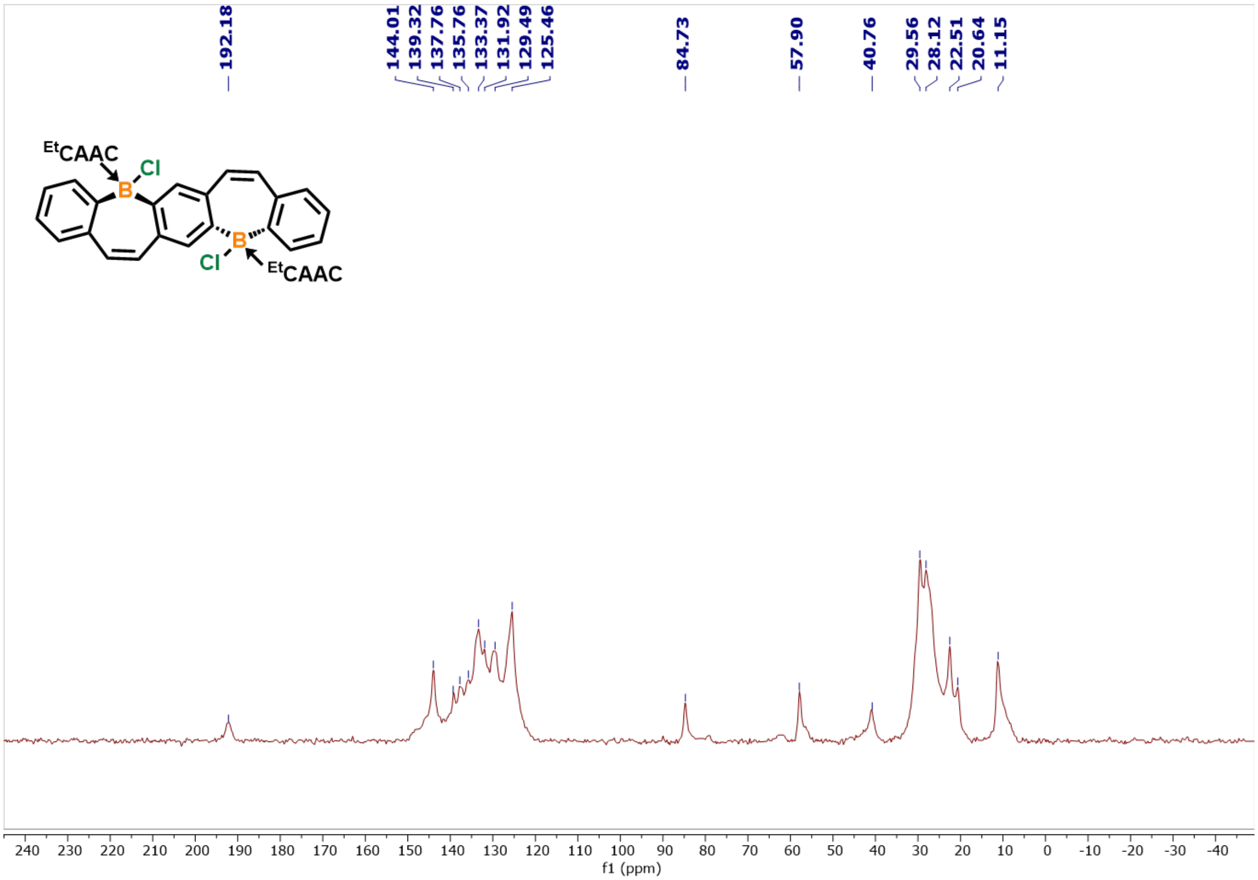

Figure S4. ^{13}C NMR (CP/MAS, 20 kHz) spectrum of **2-Cl**.

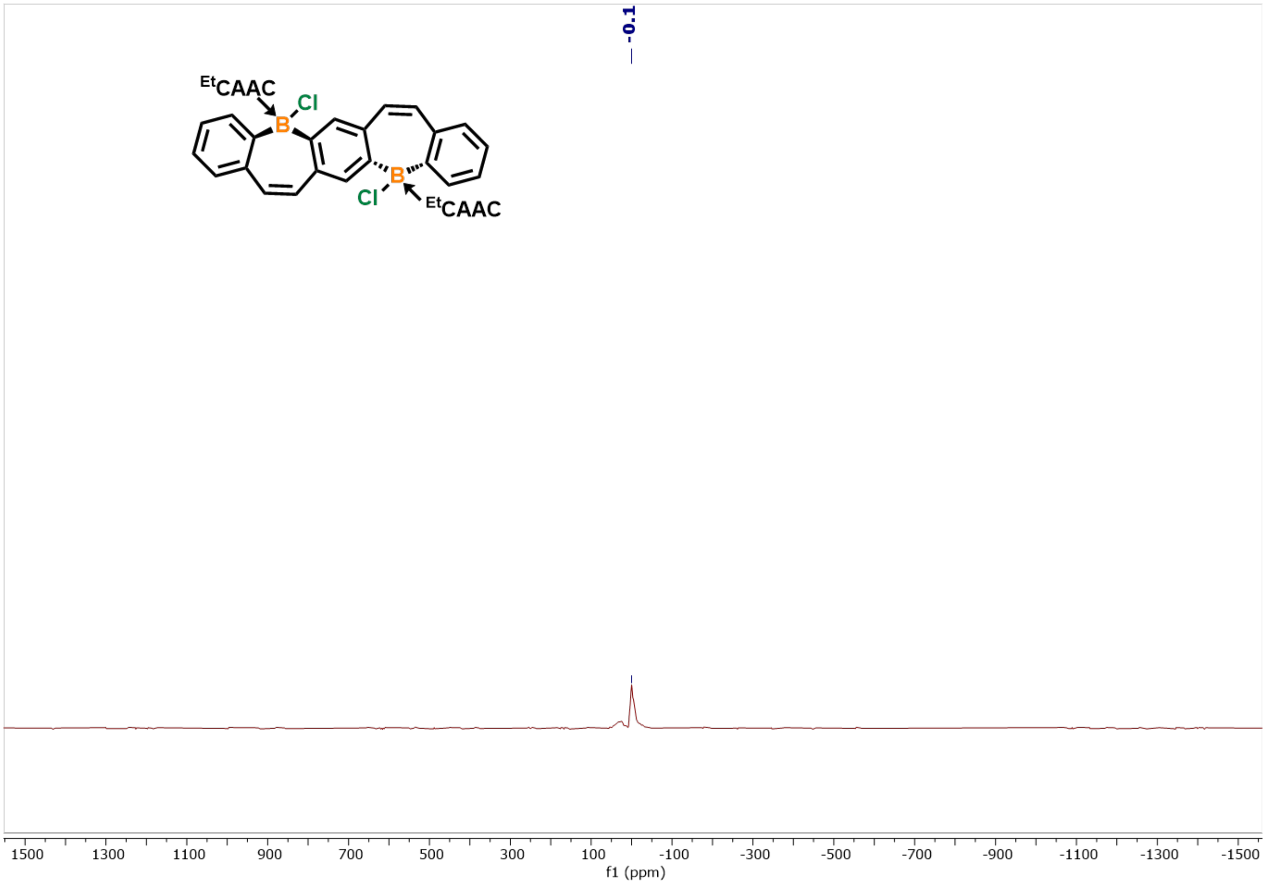

Figure S5. Solid-state ^{11}B NMR (MAS, 20 kHz) spectrum of **2-Cl**.

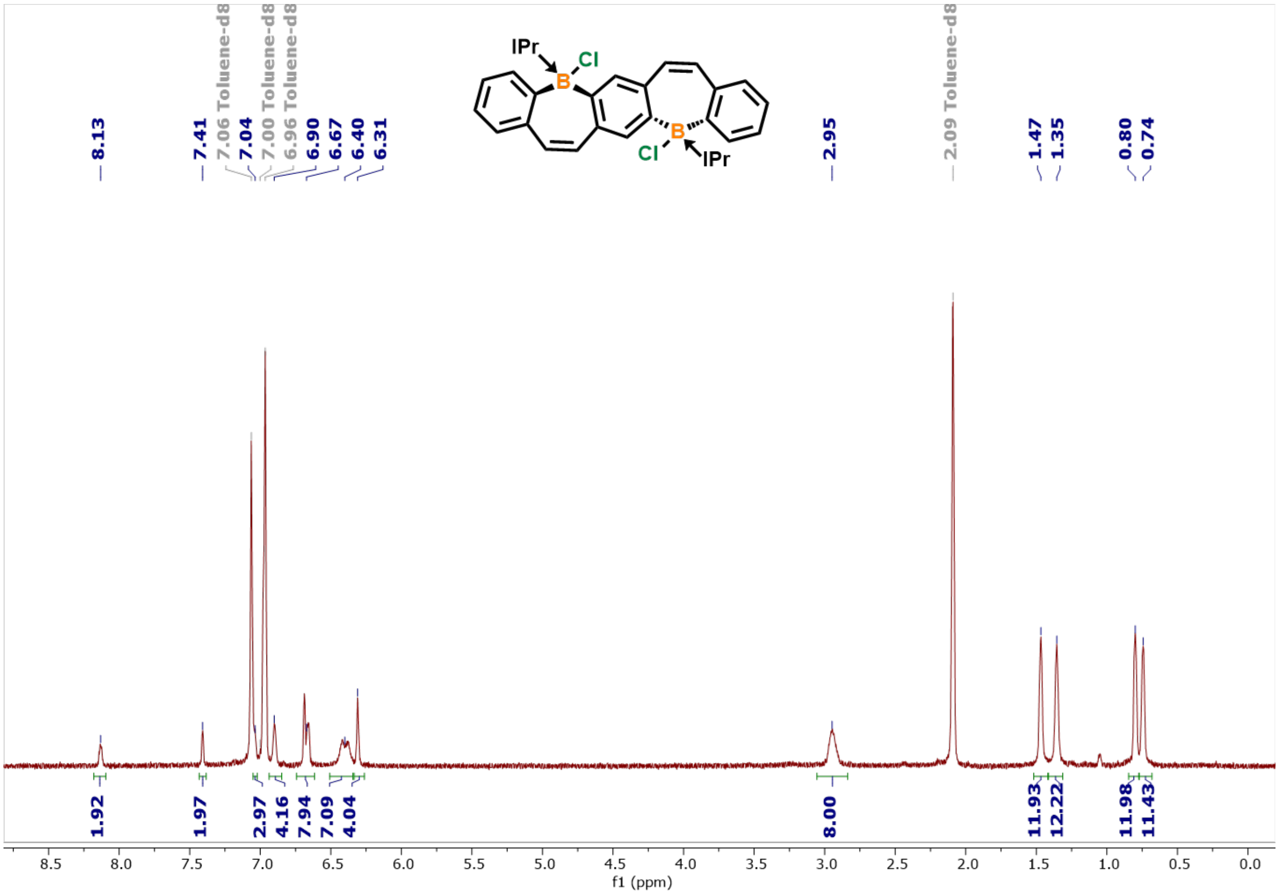

Figure S6. ^{1}H NMR (600 MHz, toluene-d$_8$, 100 °C) spectrum of **3-Cl**.

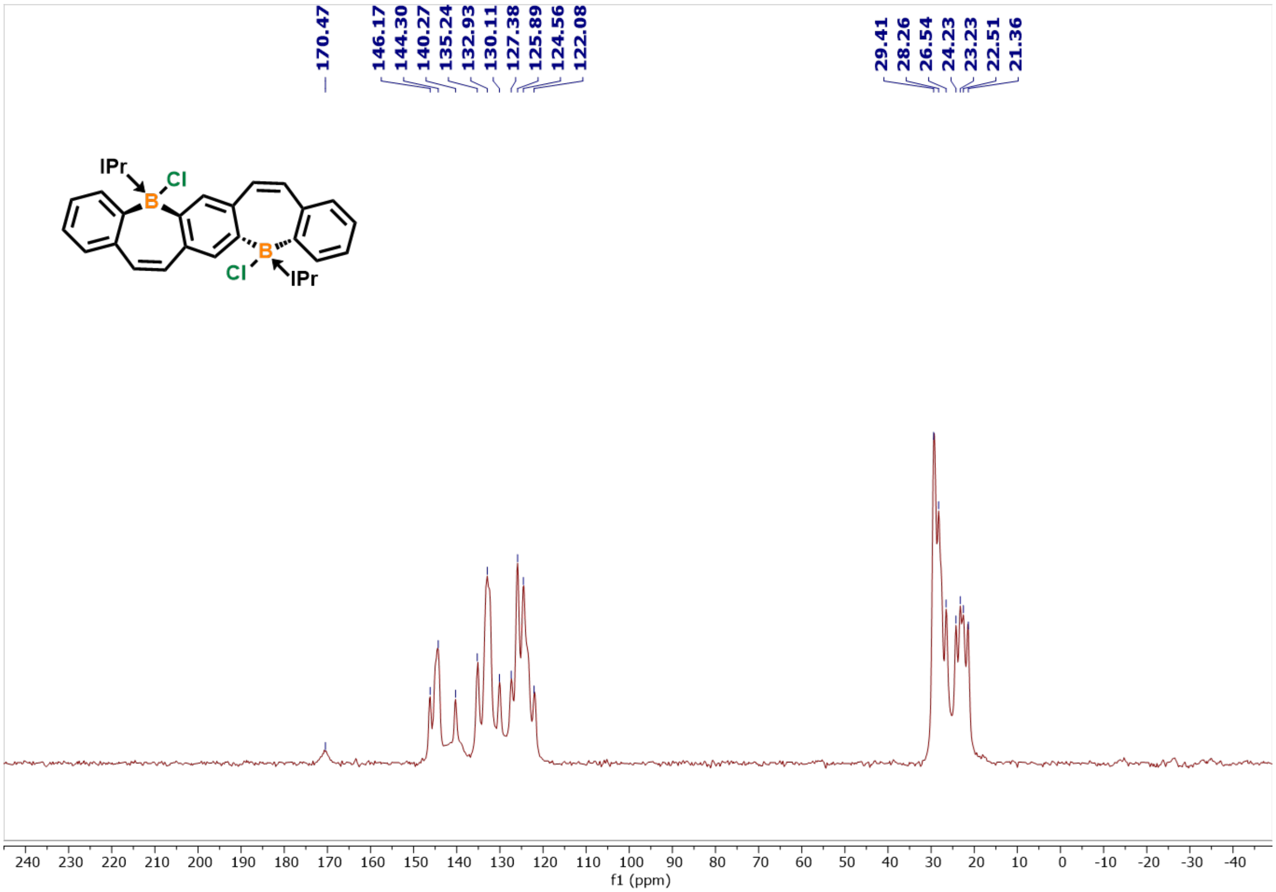

Figure S7. ^{13}C NMR spectrum (CP/MAS, 20 kHz) of **3-Cl**.

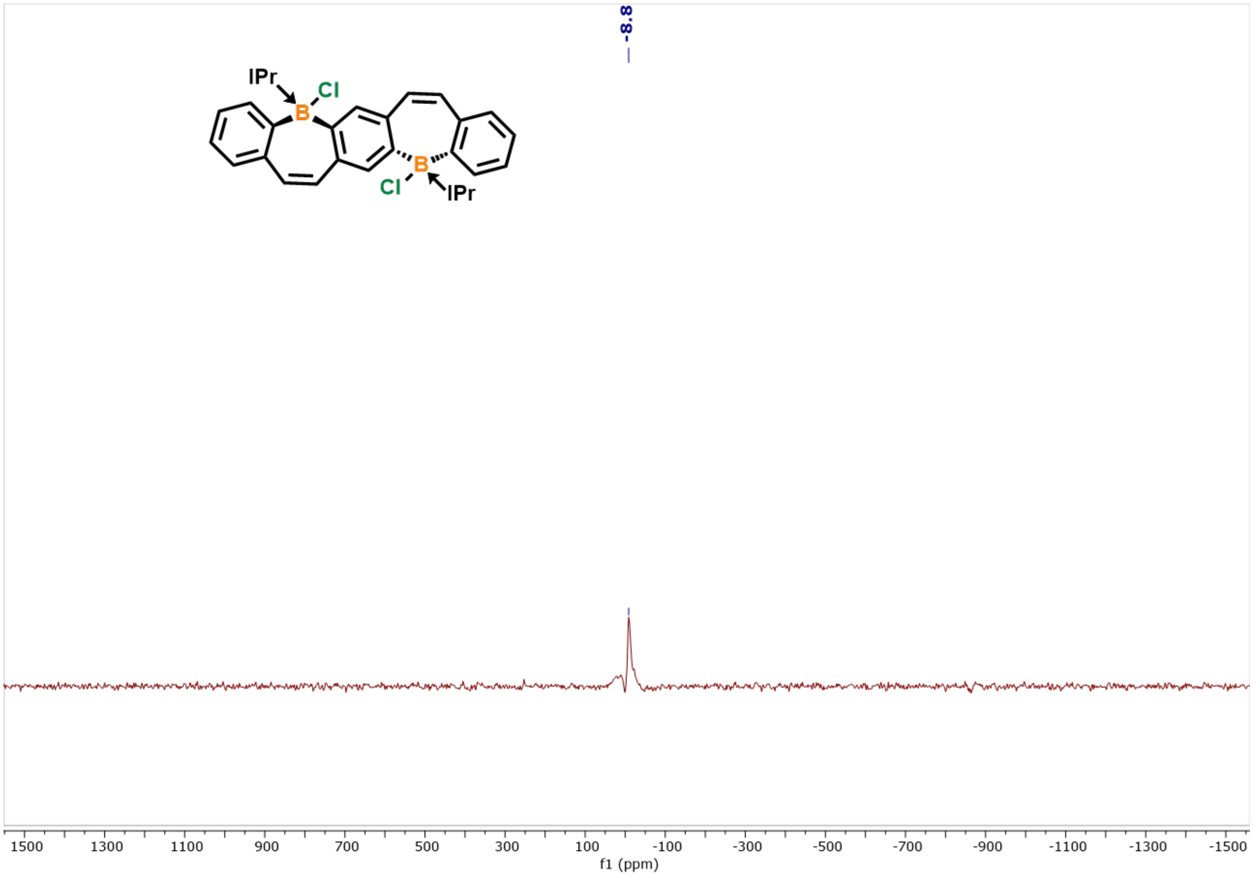

Figure S8. Solid-state ^{11}B NMR (MAS, 20 kHz) spectrum of **3-Cl**.

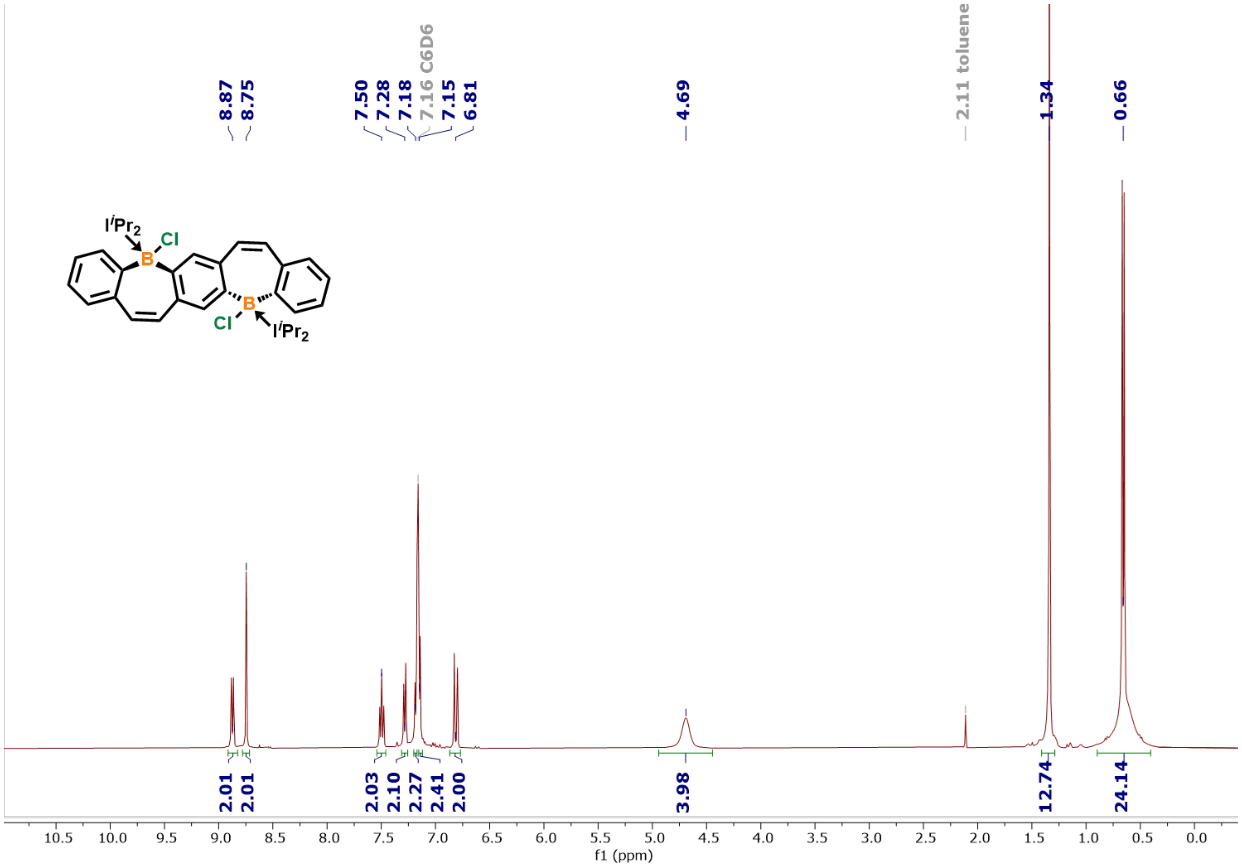

Figure S9. ^{1}H NMR (400 MHz, C_6D_6, 20 °C) of **4-Cl**.

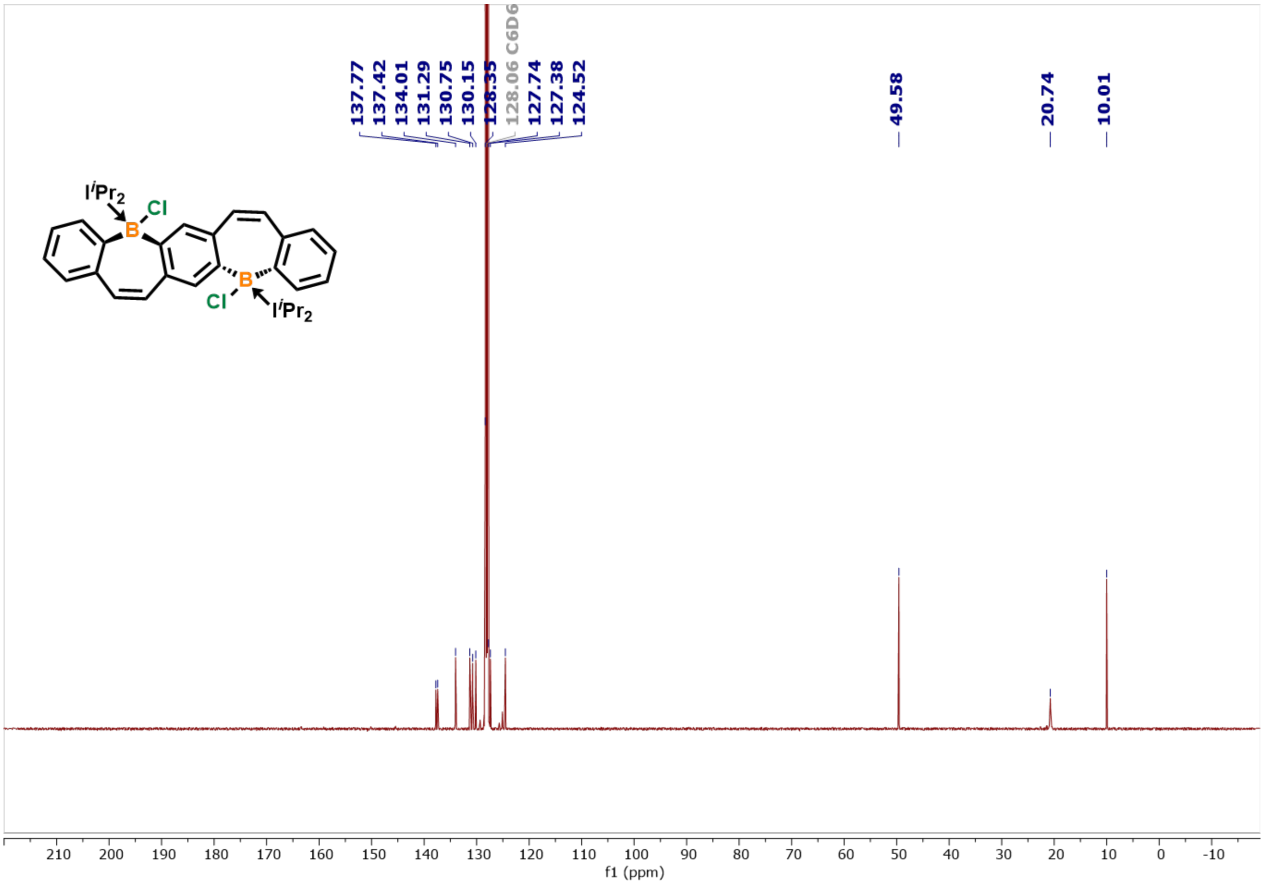

Figure S10. ^{13}C NMR (126 MHz, C_6D_6, 20 °C) spectrum of **4-Cl**.

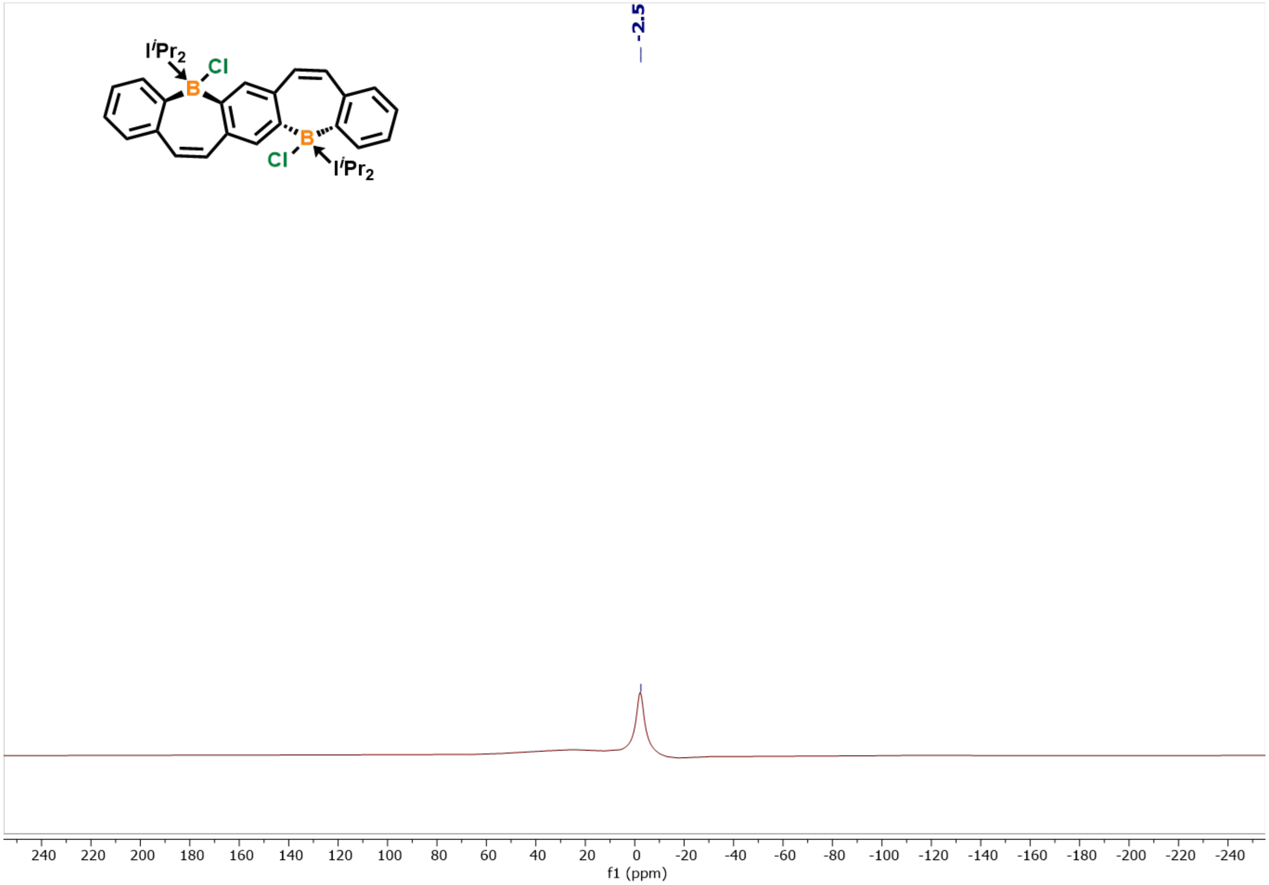

Figure S11. ^{11}B NMR (160 MHz, CD_2Cl_2, 20 °C) spectrum of **4-Cl**.

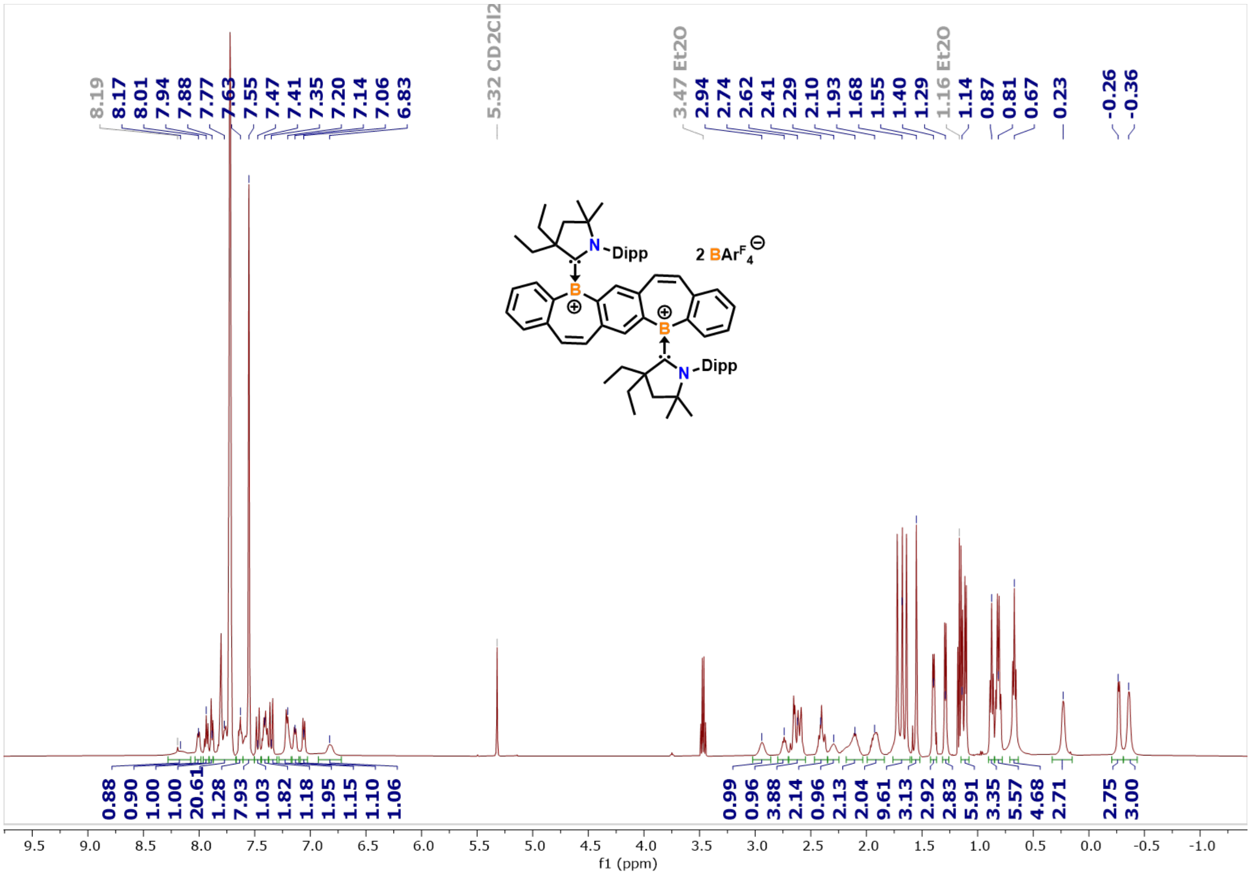

Figure S12. ^{1}H NMR (500 MHz, CD_2Cl_2, 20 °C) spectrum of **5**. There is a signal from a protonated CAAC impurity at 8.19 ppm but overlaps with the signal at 8.17 ppm from **5** making the quantification of protonated CAAC a challenge. Other signals corresponding to protonated CAAC were not able to be identified due to the complex splitting from the compound signals in the aliphatic region. Although attempts were made to obtain NMR using other deuterated solvents, NMR signals were best resolved in CD_2Cl_2 and data was obtained of concentrated samples protected from light. Residual diethyl ether is present from the reaction and labeled in the spectrum.

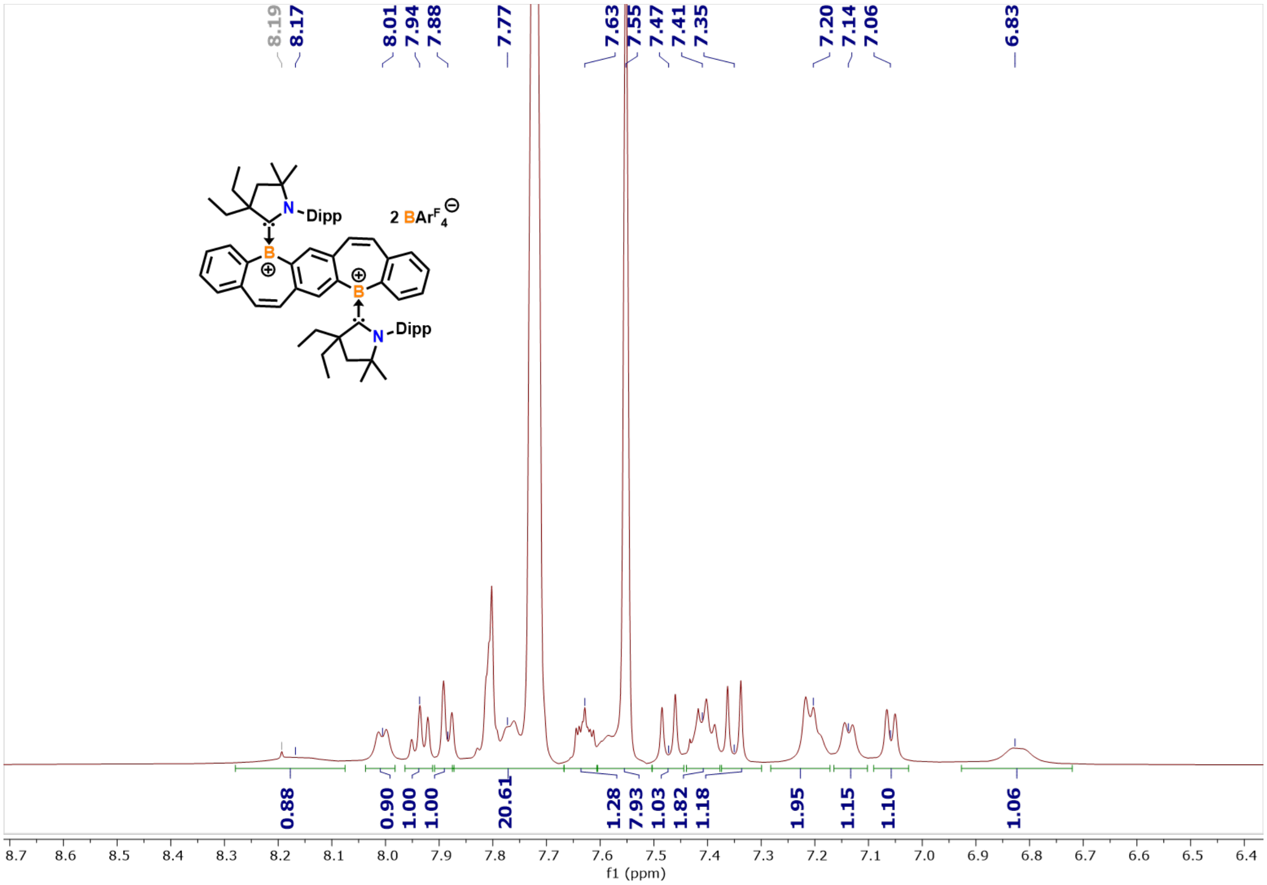

Figure S13. Zoomed in ^{1}H NMR (500 MHz, CD_2Cl_2, 20 °C) spectrum of **5** from 6.4-8.7 ppm.

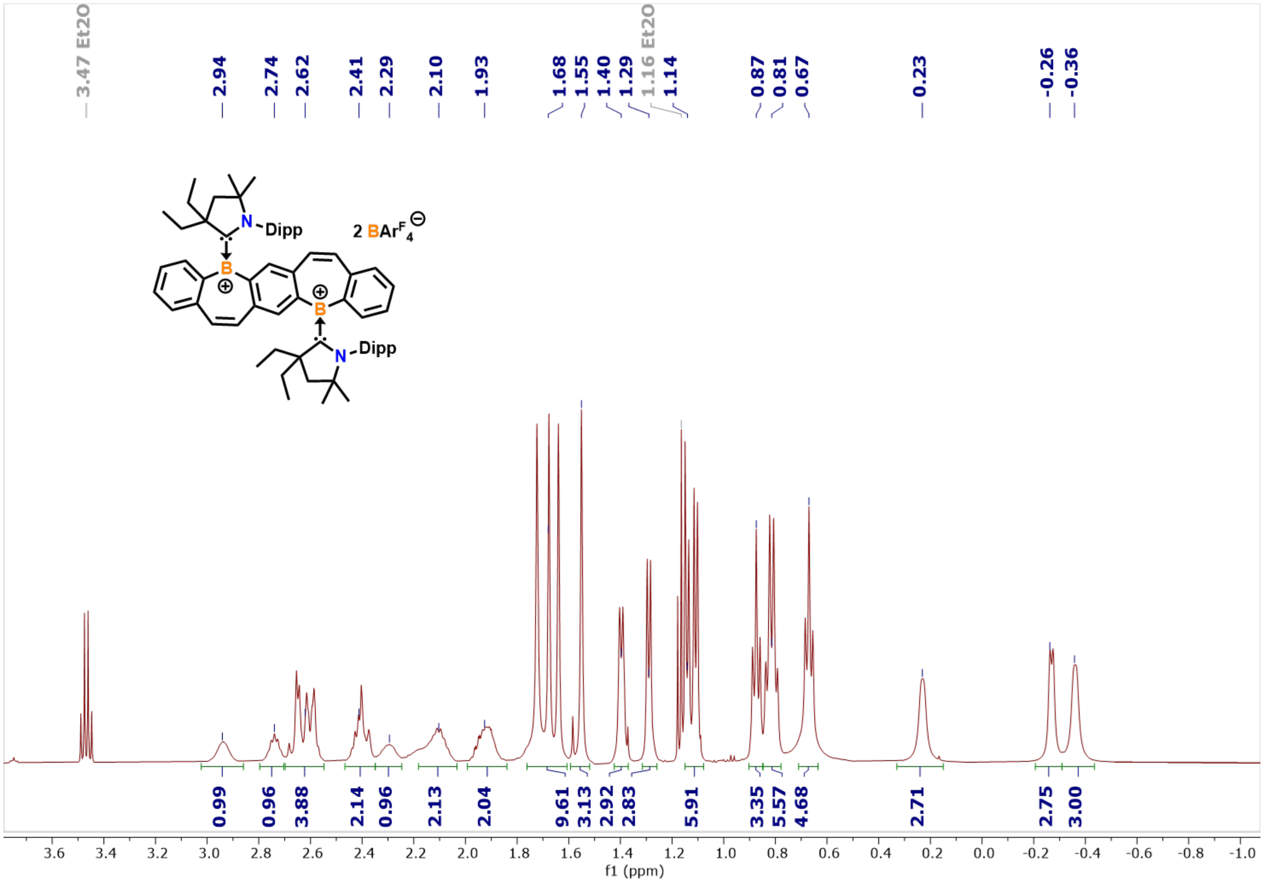

Figure S14. Zoomed in ^{1}H NMR (500 MHz, CD_2Cl_2, 20 °C) spectrum of **5** from -1.0-3.6 ppm.

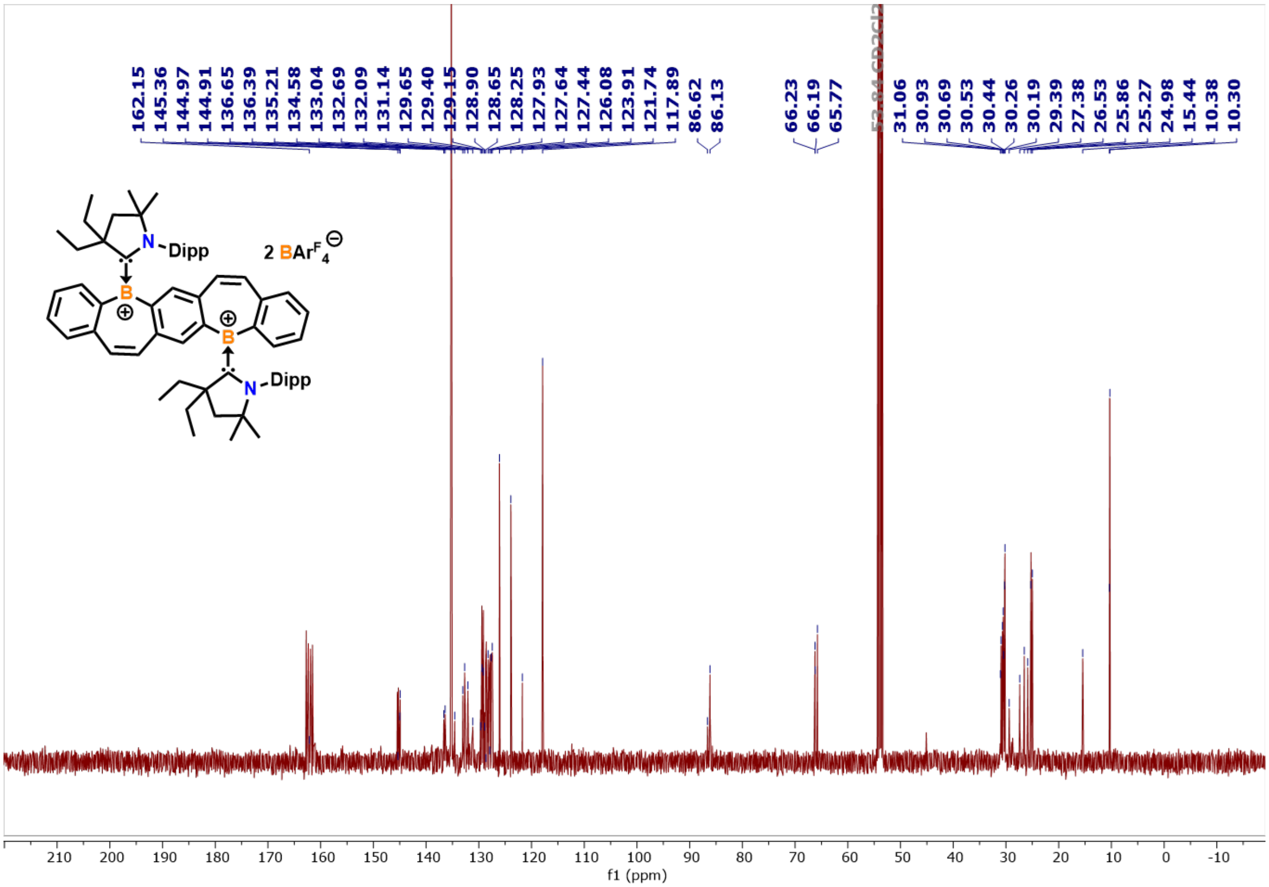

Figure S15. ^{13}C NMR (126 MHz, CD_2Cl_2, 20 °C) spectrum of **5**.

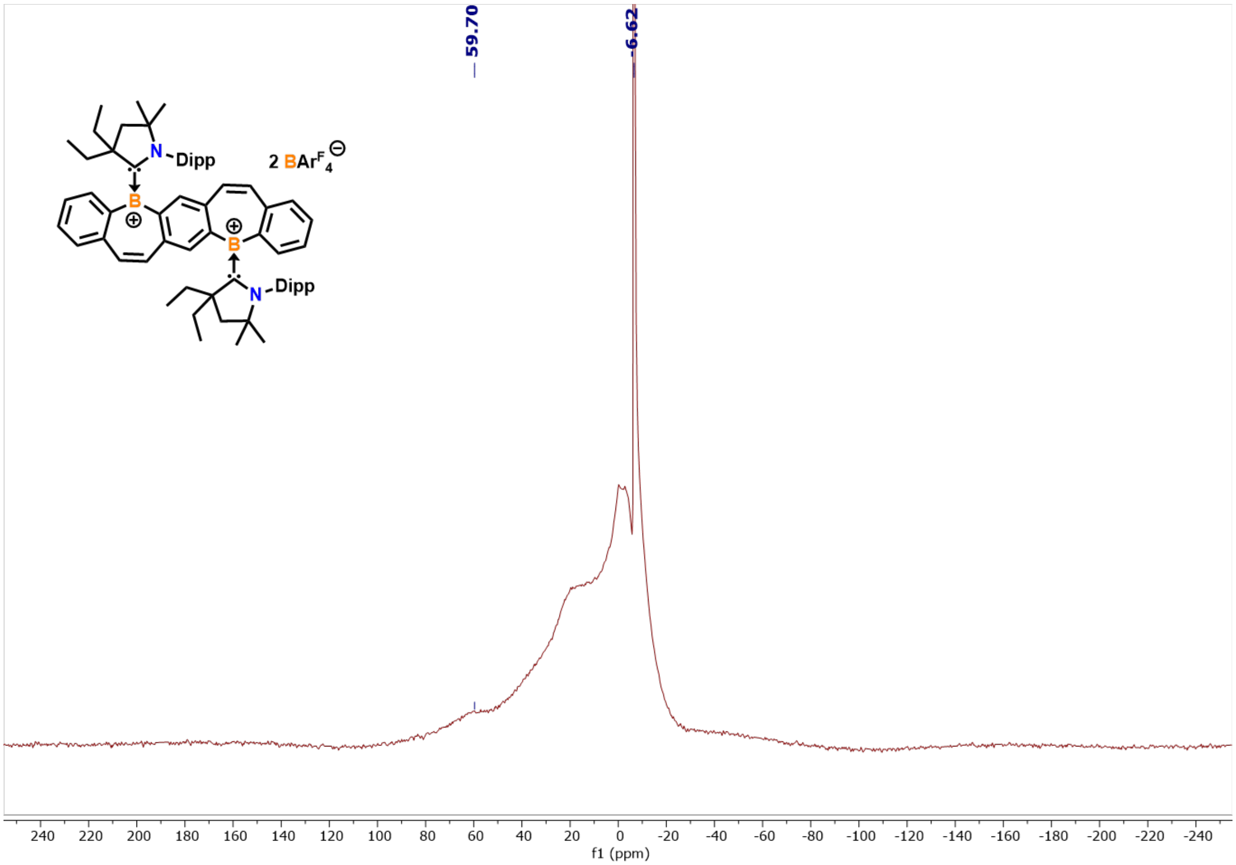

Figure S16. $^{11}B\{^1H\}$ NMR (160 MHz, CD_2Cl_2, 20 °C) spectrum of **5**. Borosilicate probe could not be fully processed out due to weak and broad signal at 59.7 ppm having a similar frequency.

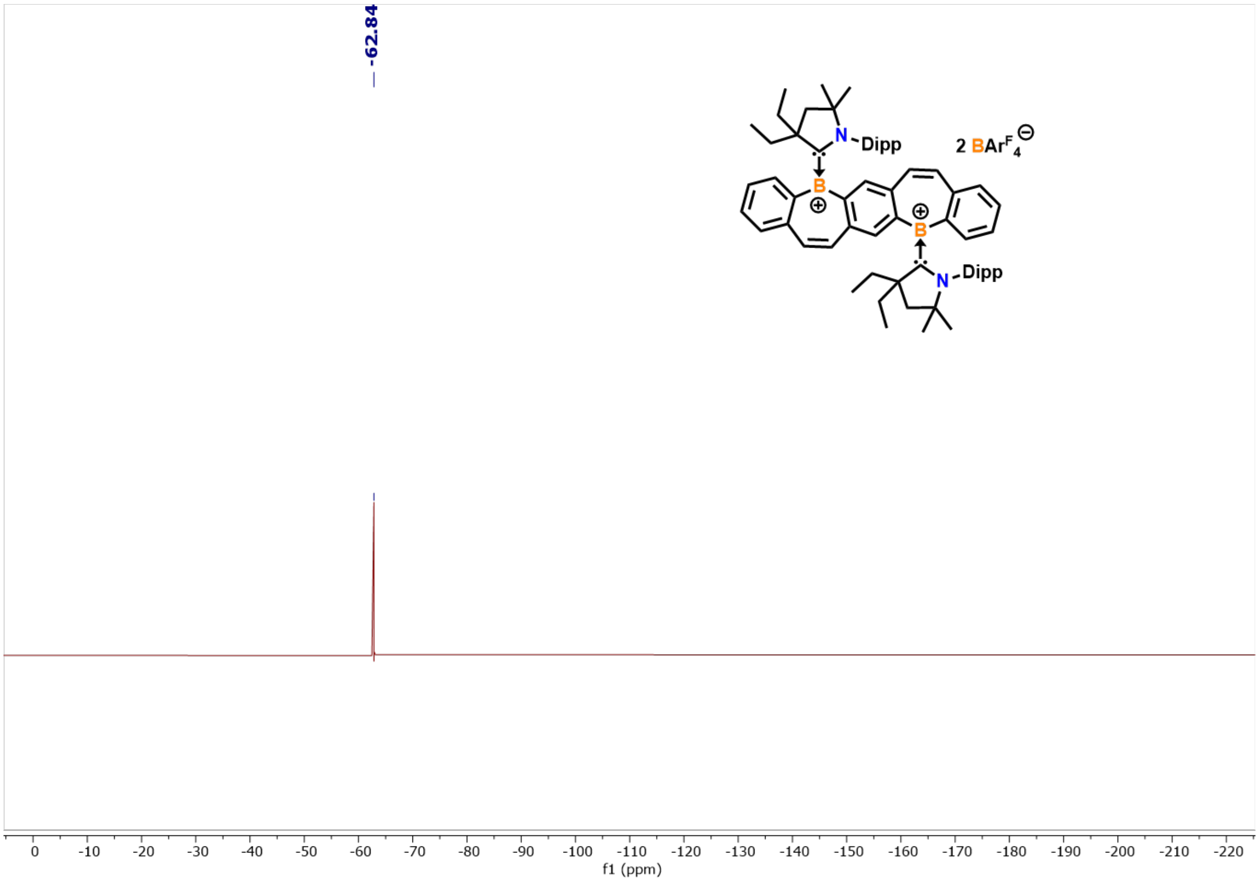

Figure S17. ^{19}F NMR (471 MHz, CD_2Cl_2, 20 °C) spectrum of **5**.

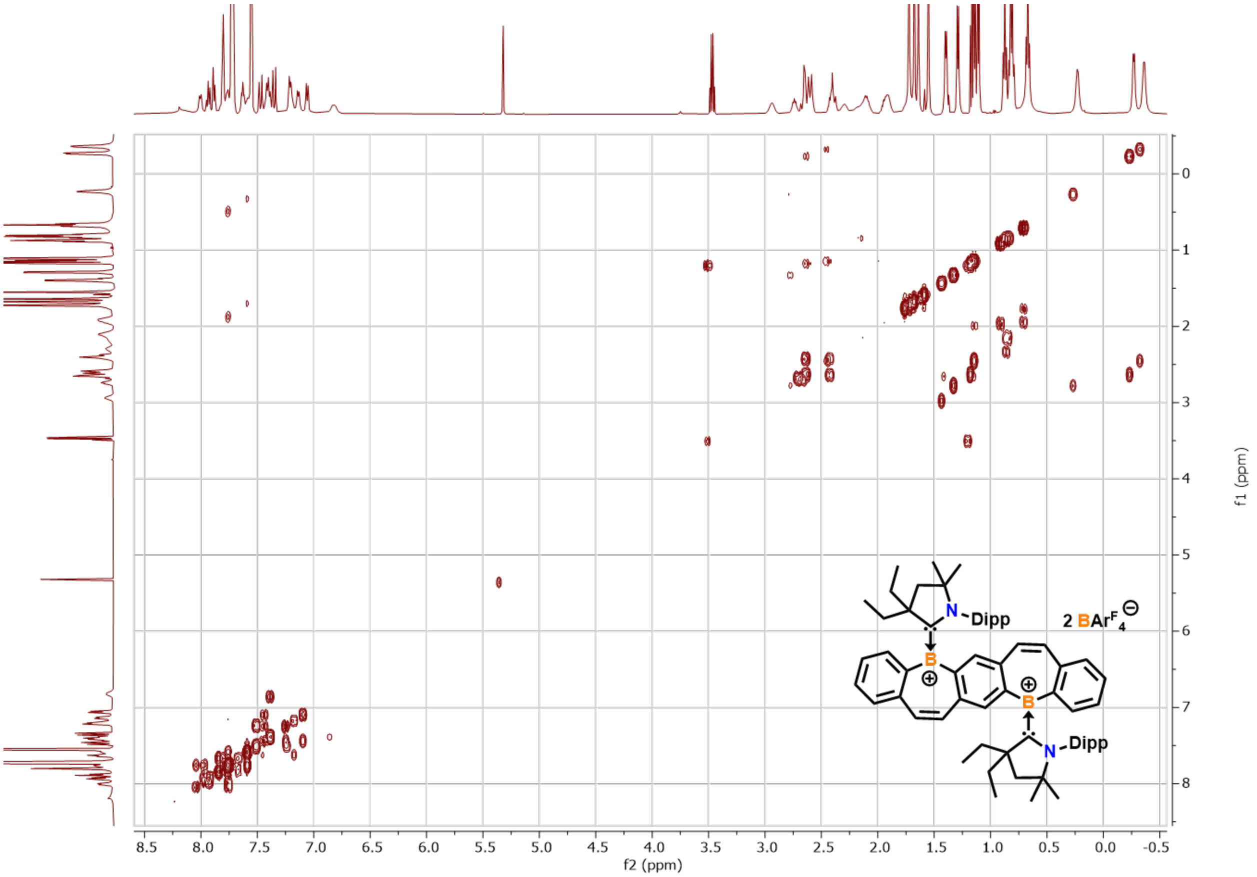

Figure S18. ^{1}H-^{1}H correlated spectroscopy [COSY] (CD_2Cl_2, 20 °C) spectrum of **5**.

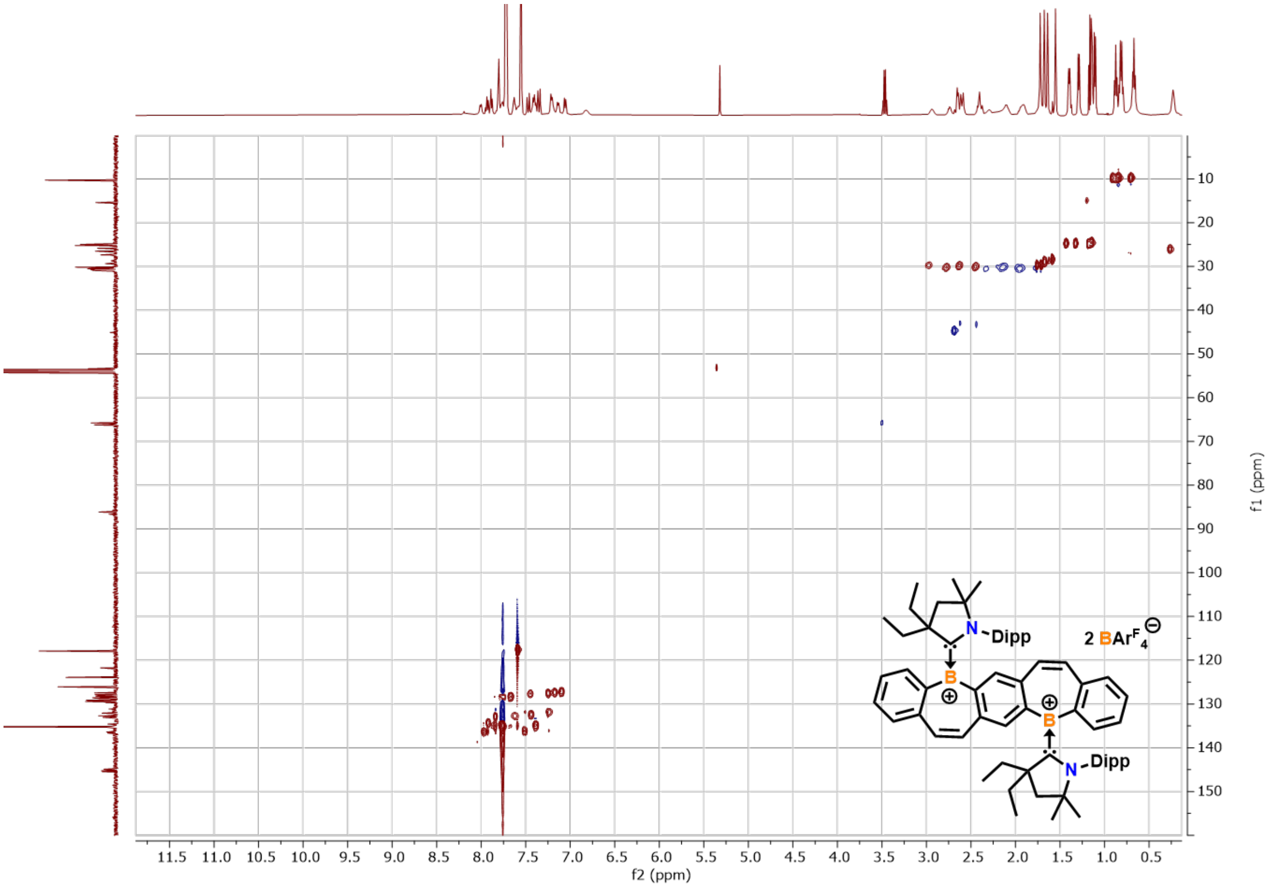

Figure S19. Heteronuclear single quantum coherence spectroscopy [HSQC] (CD_2Cl_2, 20 °C) spectrum of **5**.

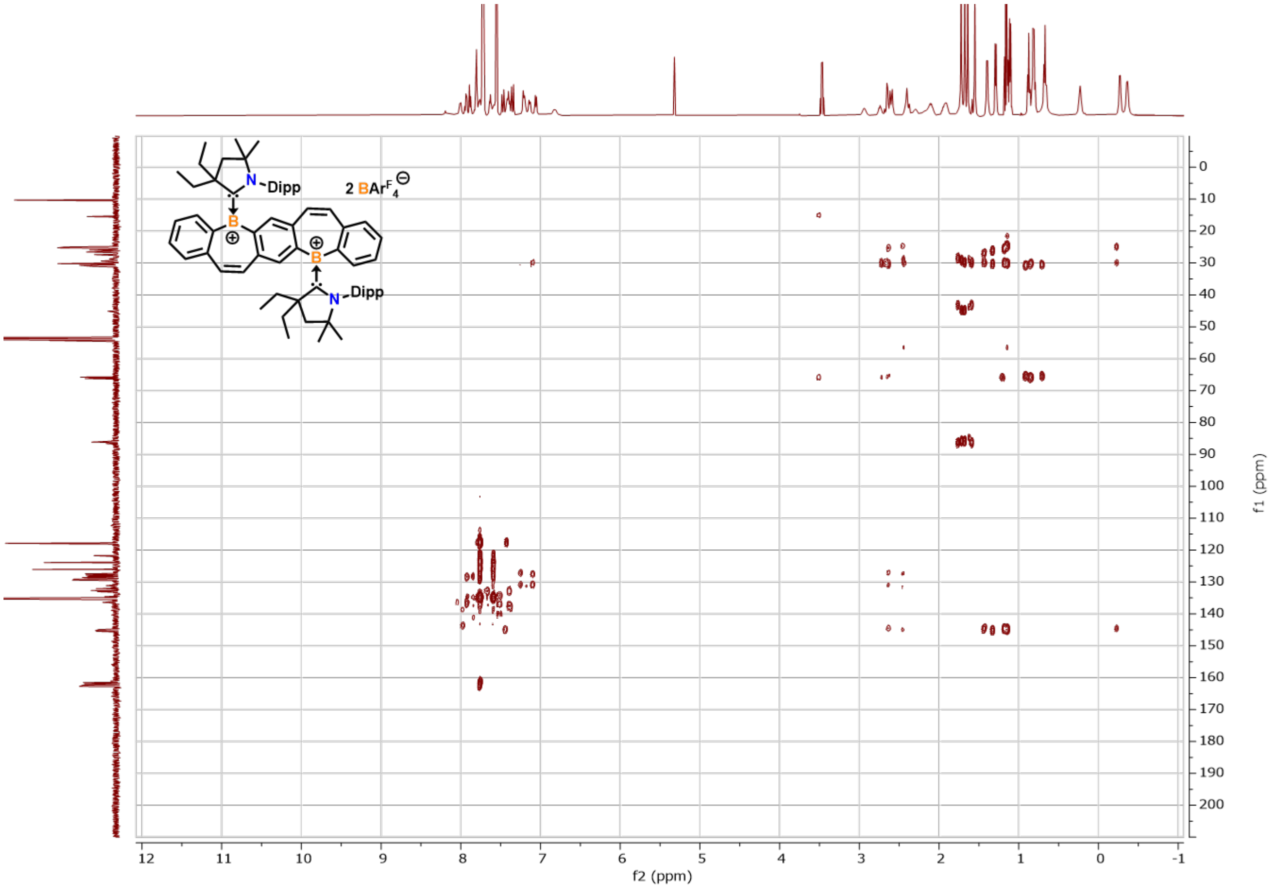

Figure S20. Heteronuclear multiple bond correlation spectroscopy [HMBC] (CD_2Cl_2, 20 °C) spectrum of **5**.

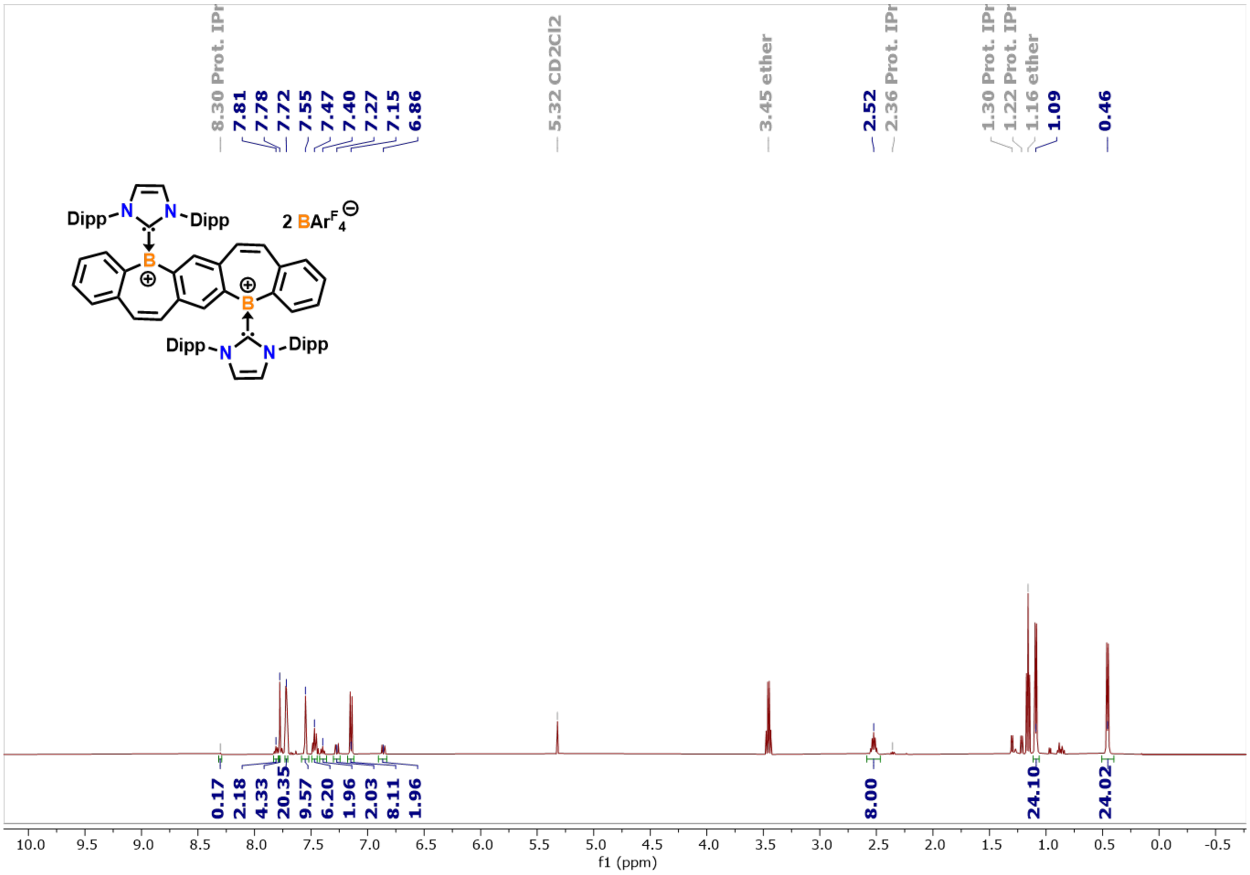

Figure S21. ^{1}H NMR (500 MHz, CD_2Cl_2, 20 °C) spectrum of **6**. There is a signal from a protonated IPr impurity at 8.30 ppm that integrates to 0.17. Other signals corresponding to protonated IPr are labeled in grey. Although attempts were made to obtain NMR using other deuterated solvents, NMR signals were best resolved in CD_2Cl_2 and data was obtained of concentrated samples protected from light. Residual diethyl ether is present from the reaction and labeled in the spectrum.

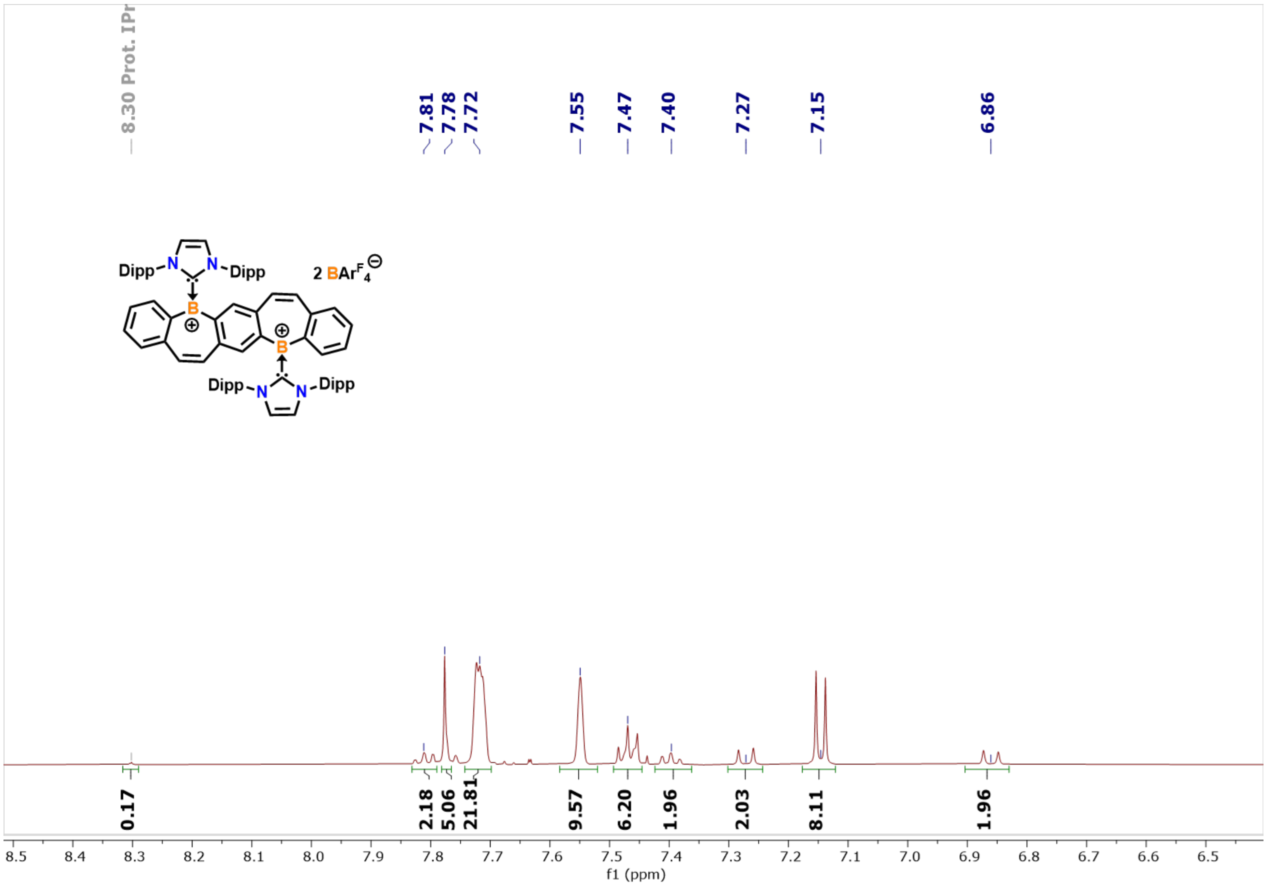

Figure S22. Zoomed in ^{1}H NMR (500 MHz, CD_2Cl_2, 20 °C) spectrum of **6** from 6.5-8.5 ppm.

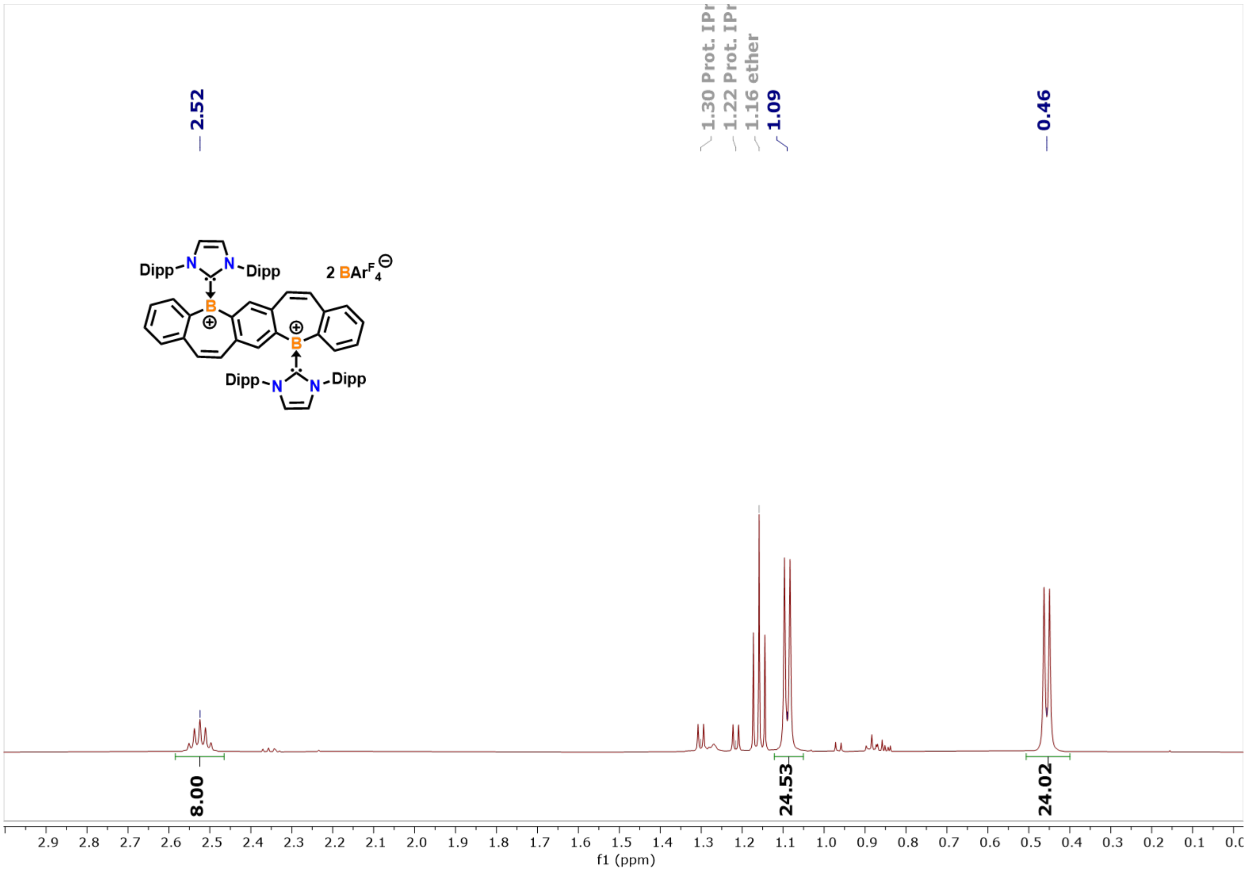

Figure S23. Zoomed in ^{1}H NMR (500 MHz, CD_2Cl_2, 20 °C) spectrum of **6** from 0.0-2.9 ppm.

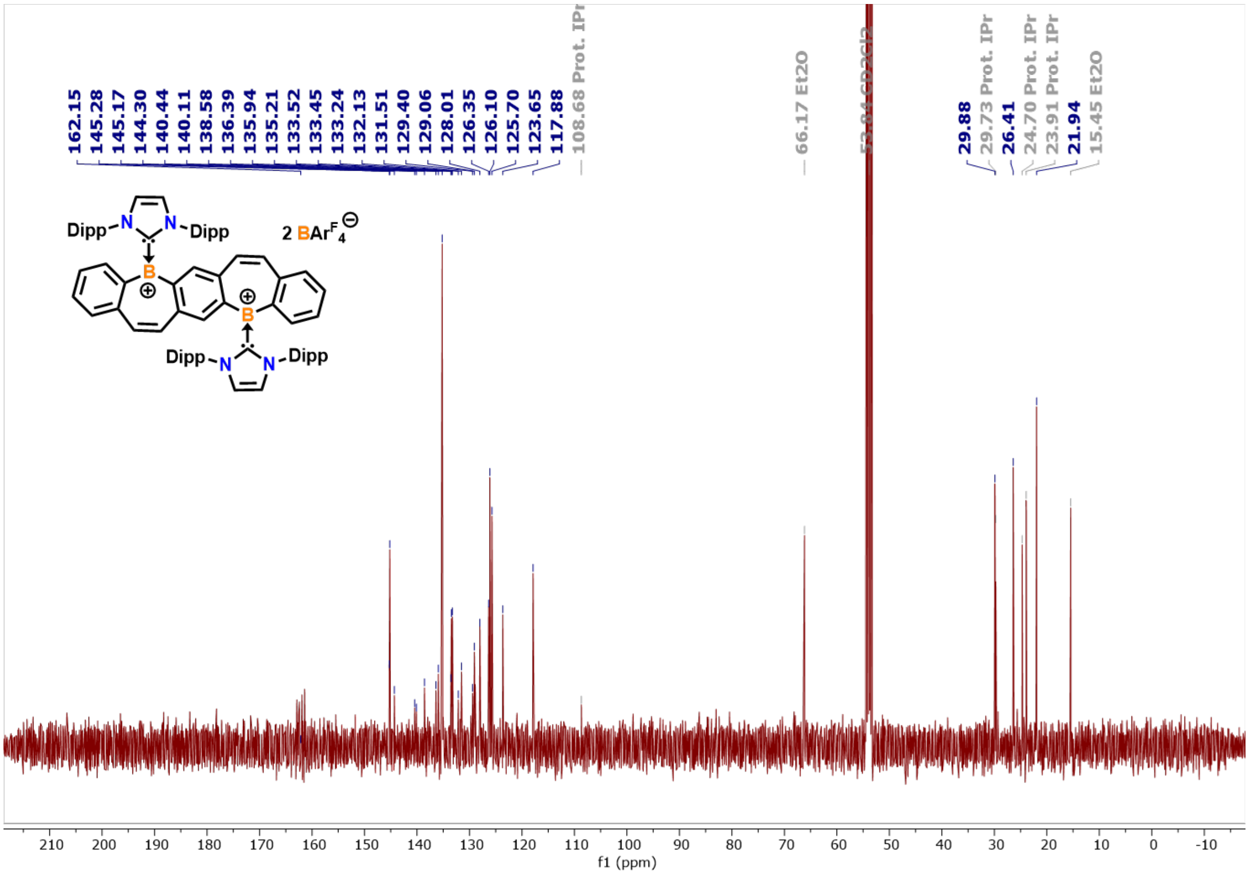

Figure S24. ^{13}C NMR (101 MHz, CD_2Cl_2) spectrum of **6**.

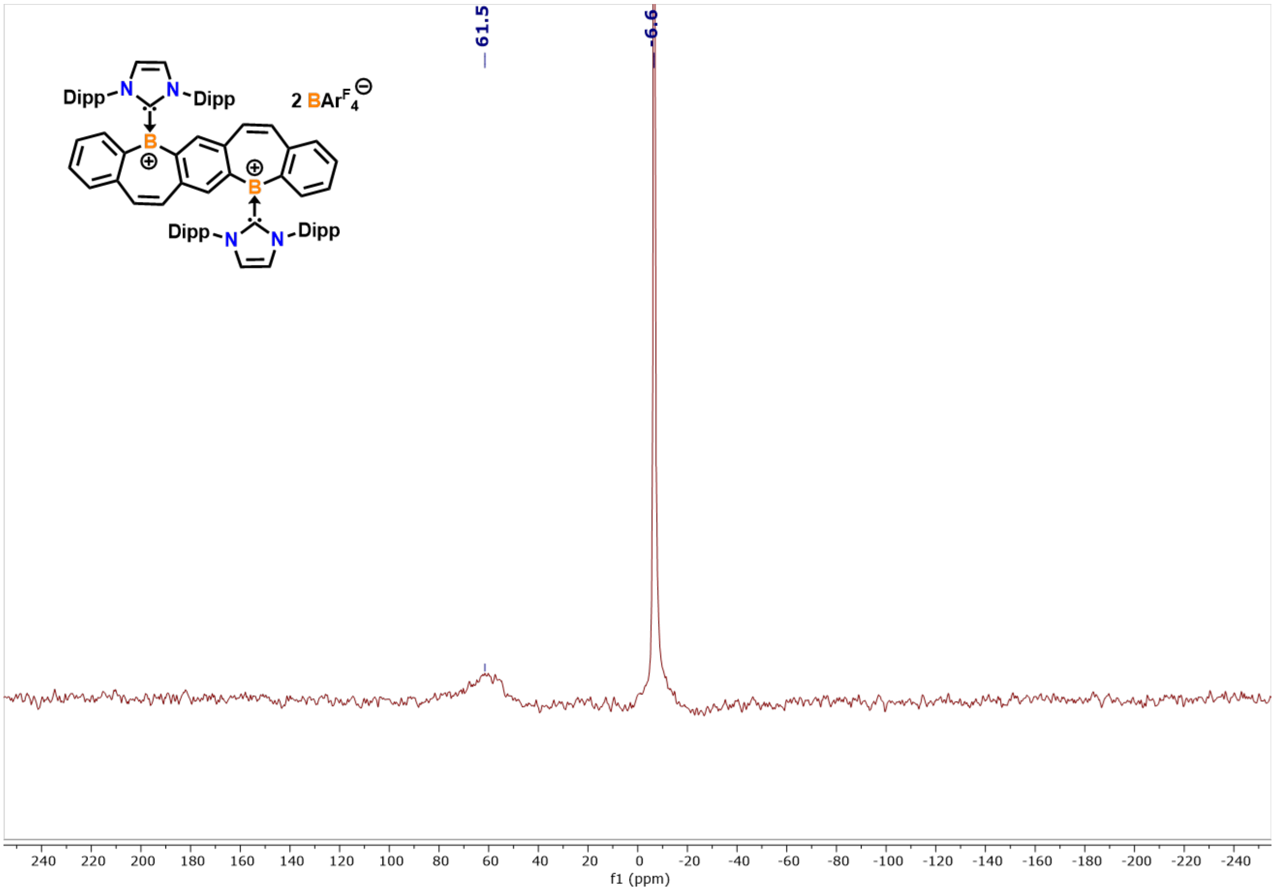

Figure S25. $^{11}B\{^{1}H\}$ NMR (161 MHz, CD_2Cl_2, 20 °C) spectrum of **6**.

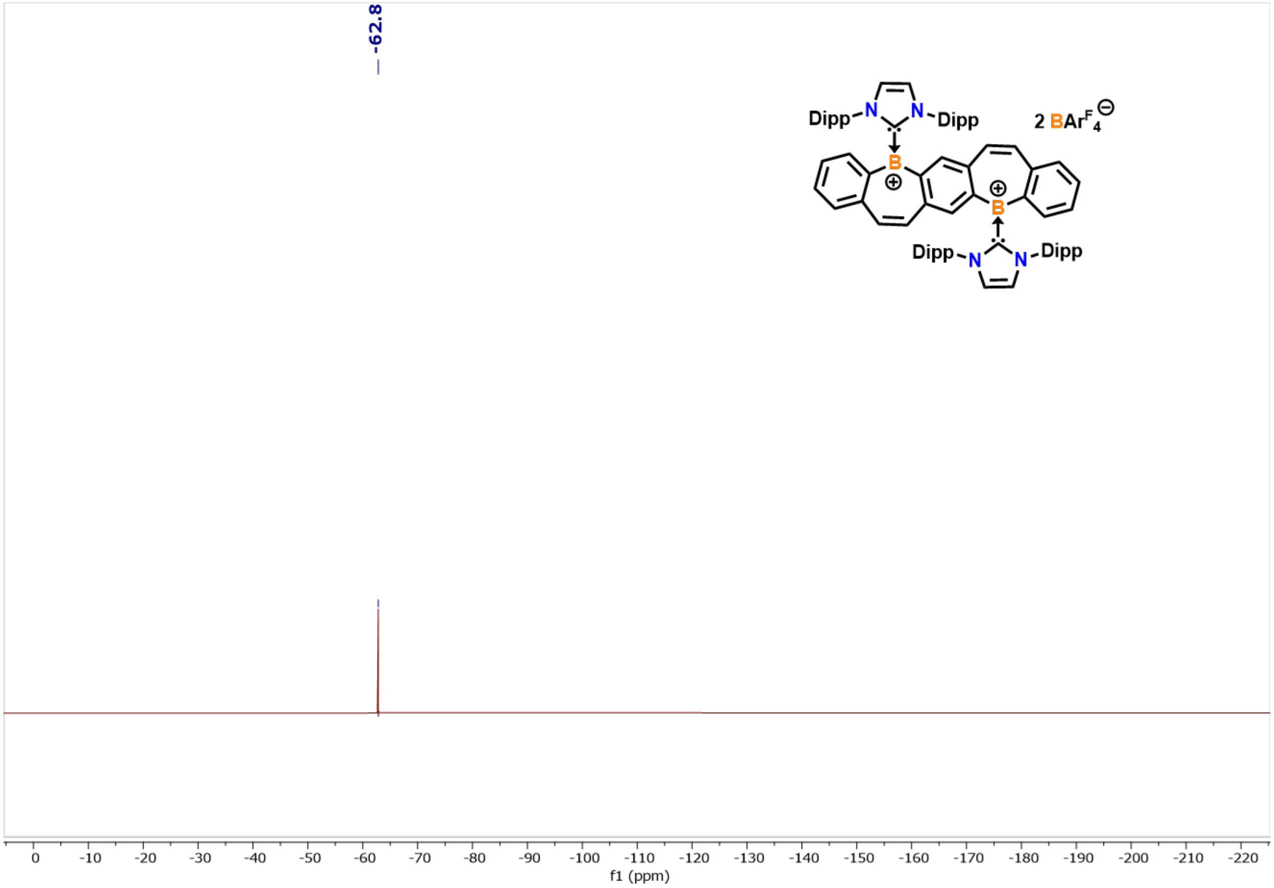

Figure S26. ^{19}F NMR (471 MHz, CD_2Cl_2, 20 °C) spectrum of **6**.

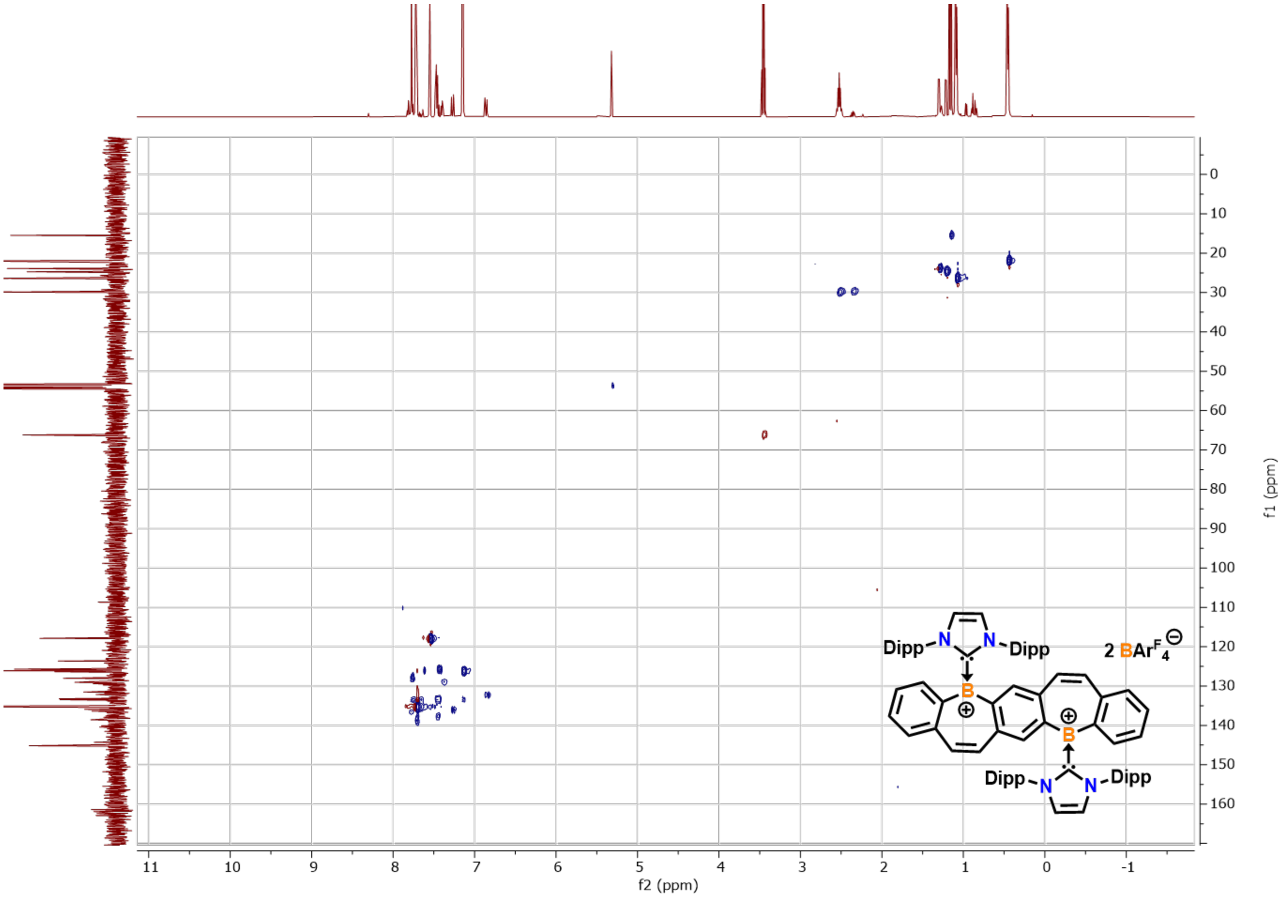

Figure S27. Heteronuclear single quantum coherence spectroscopy [HSQC] (CD_2Cl_2, 20 °C) spectrum of **6**.

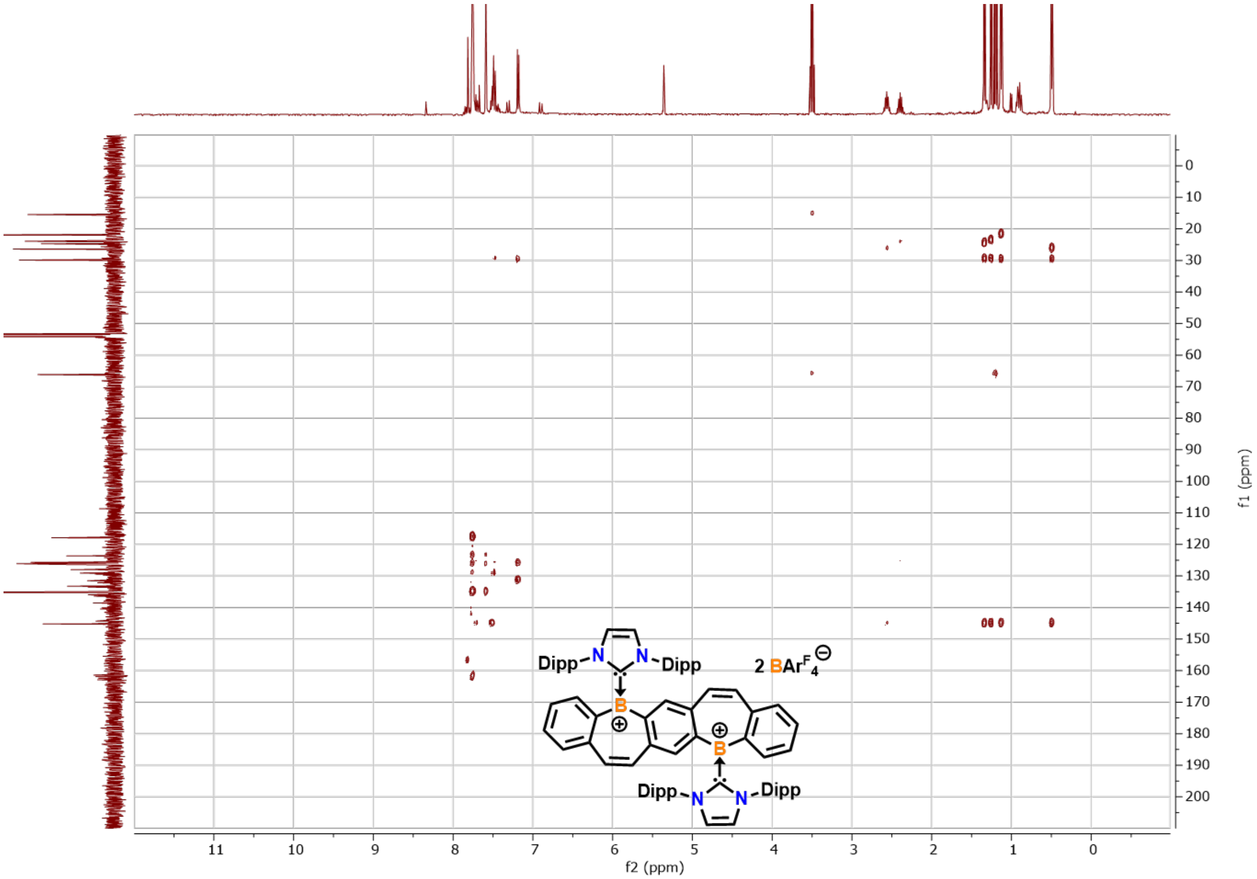

Figure S28. Heteronuclear multiple bond correlation spectroscopy [HMBC] (CD_2Cl_2, 20 °C) spectrum of **6**.

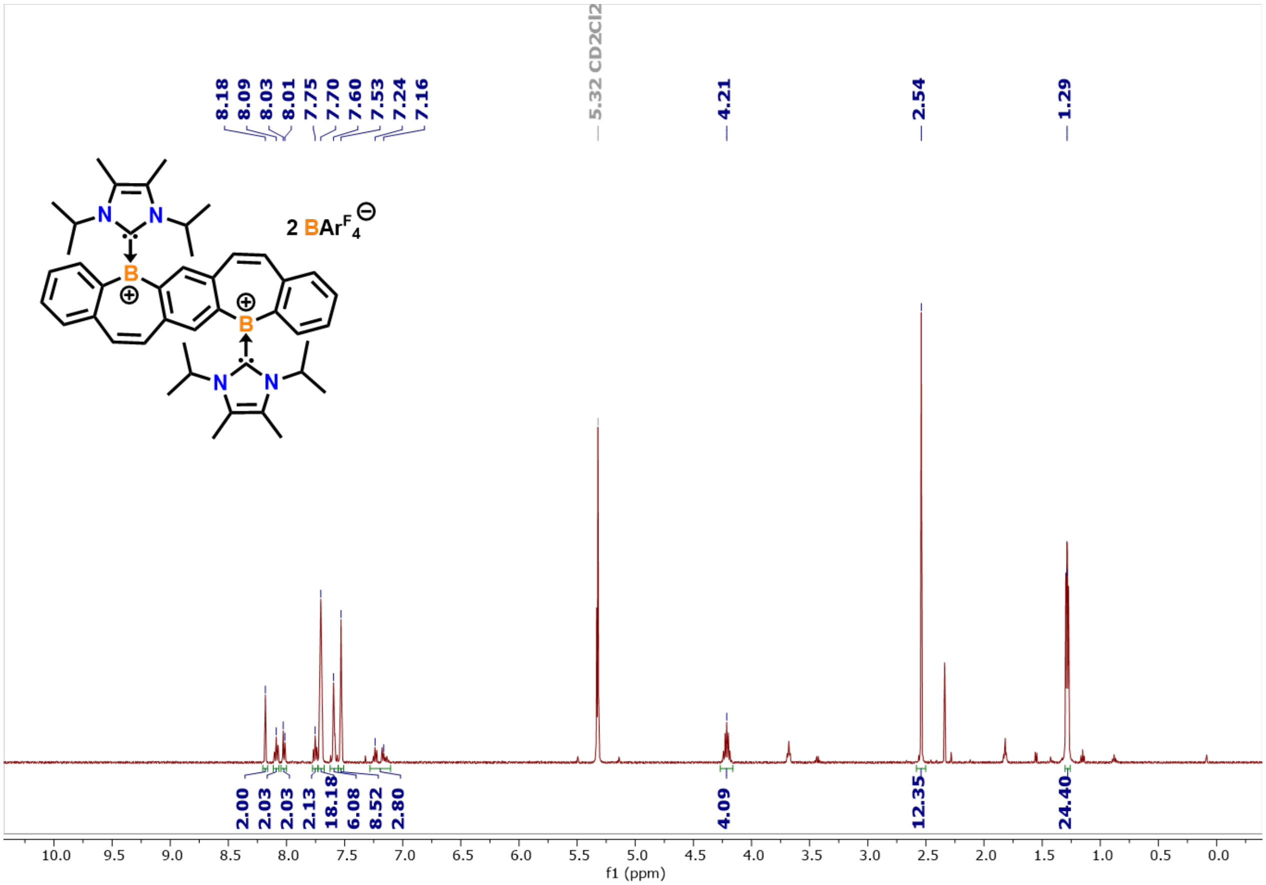

Figure S29. ^{1}H NMR (500 MHz, CD_2Cl_2, 20 °C) spectrum of **7**.

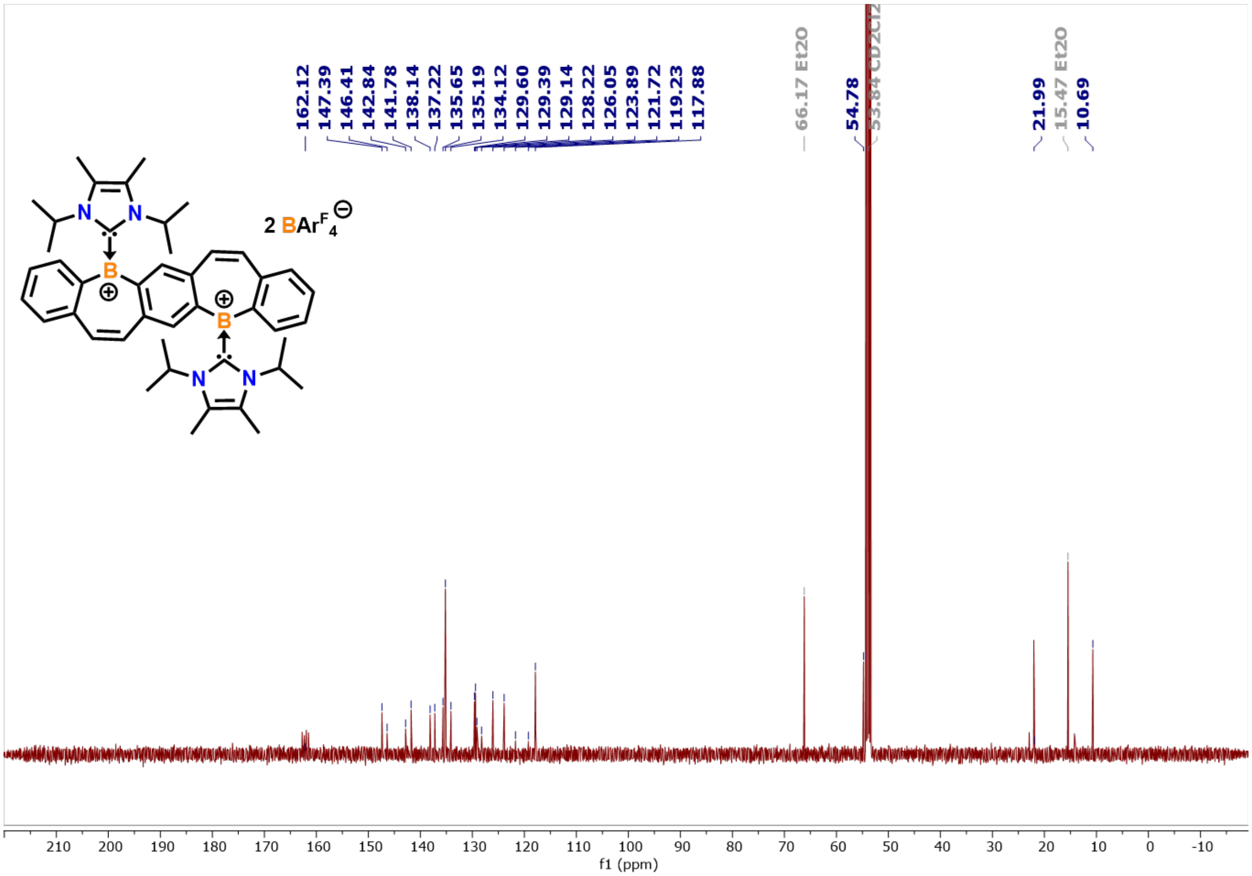

Figure S30. ^{13}C NMR (126 MHz, CD_2Cl_2, 20 °C) spectrum of **7**.

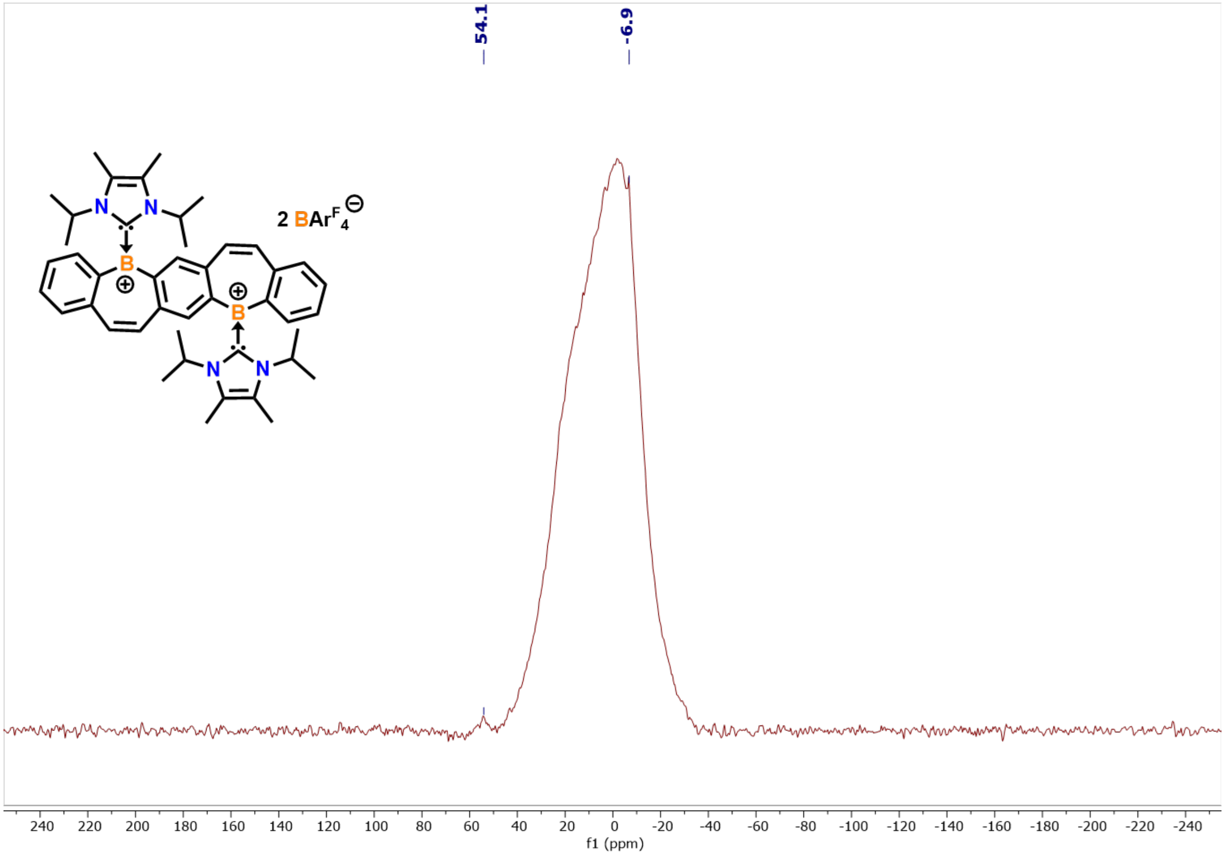

Figure S31. ^{11}B NMR (161 MHz, CD_2Cl_2, 20 °C) spectrum of **7**. The borosilicate probe could not be fully processed out without removing the weak and broad compound signal at 54.1 ppm due it having a similar frequency to the probe.

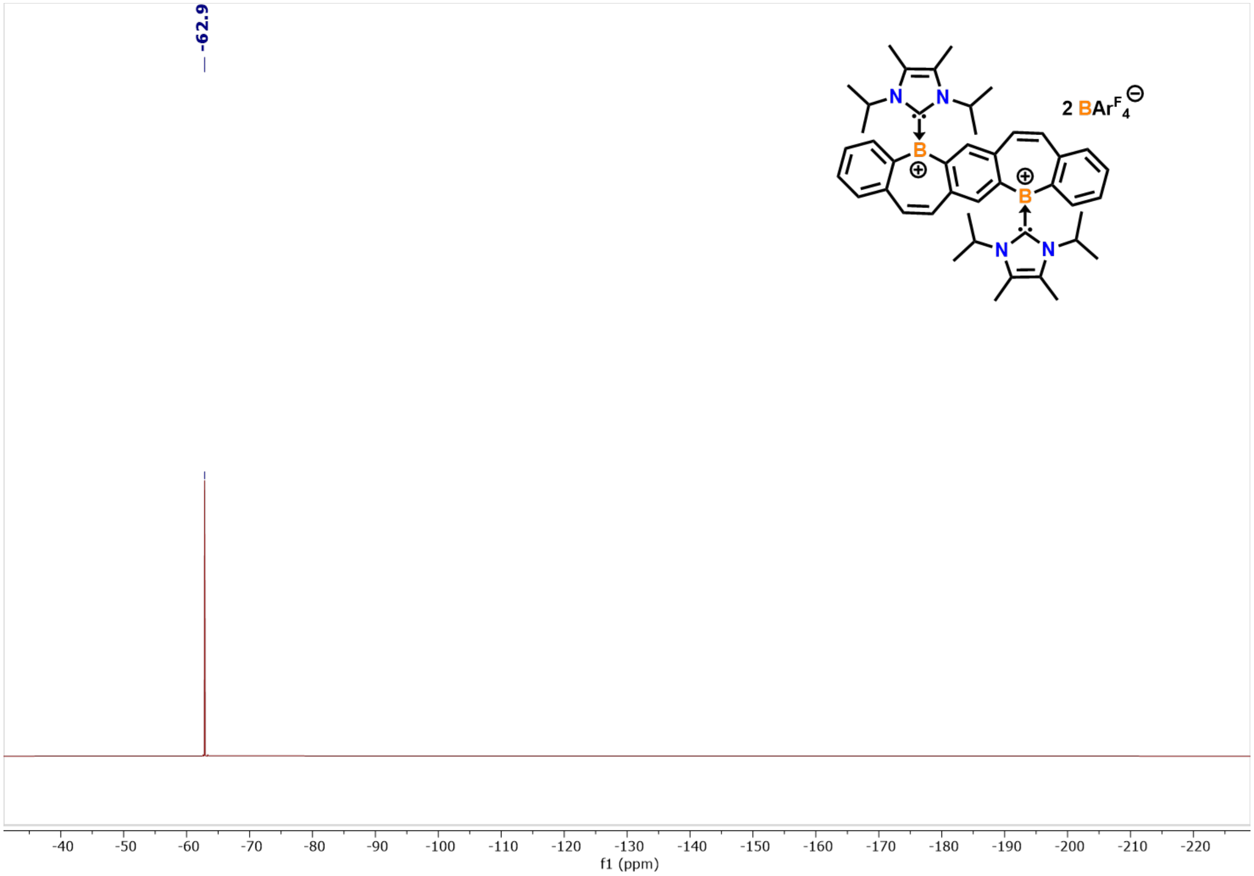

Figure S32. ^{19}F NMR (376 MHz, CD_2Cl_2, 20 °C) spectrum of **7**.

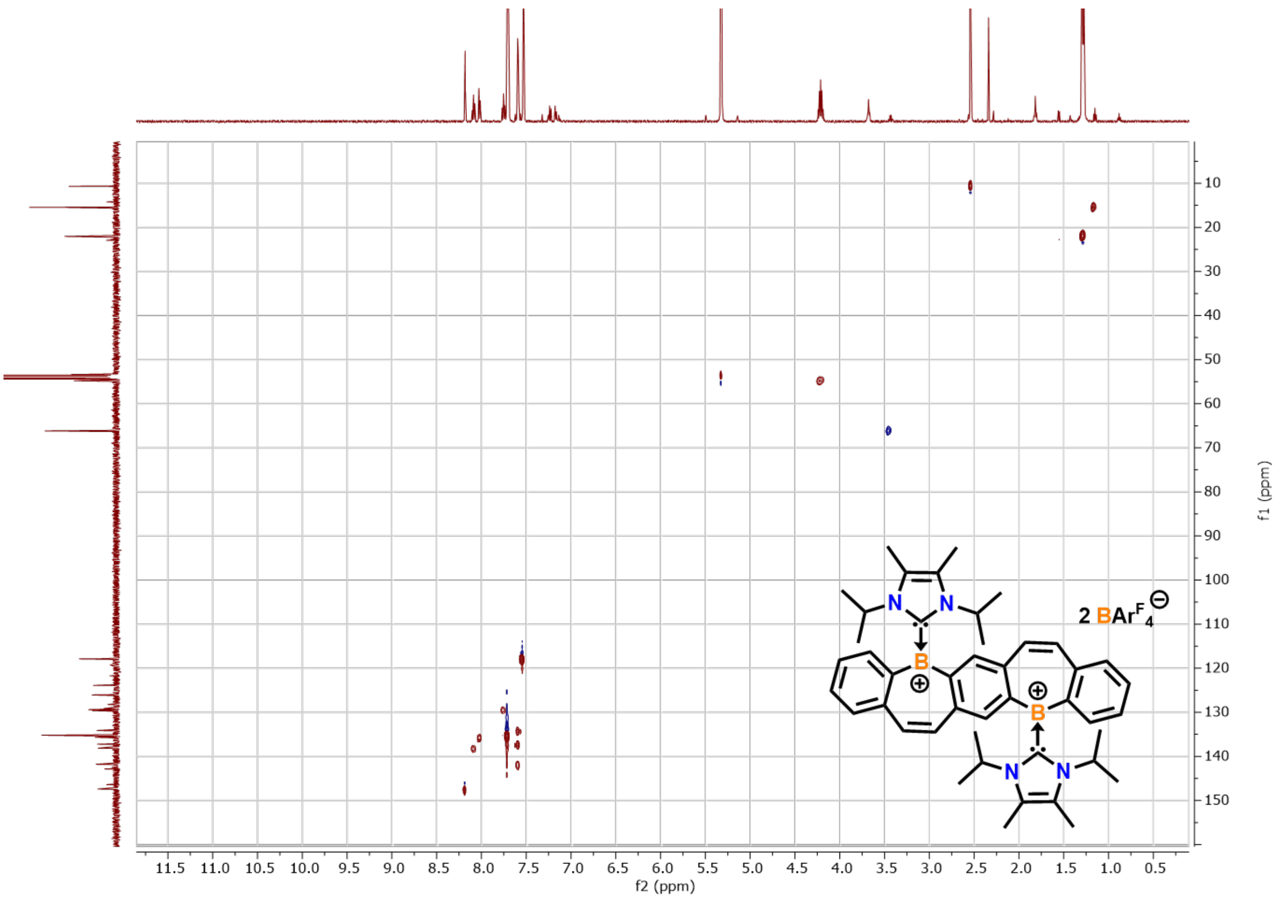

Figure S33. Heteronuclear single quantum coherence spectroscopy [HSQC] (CD_2Cl_2, 20 °C) spectrum of **7**.

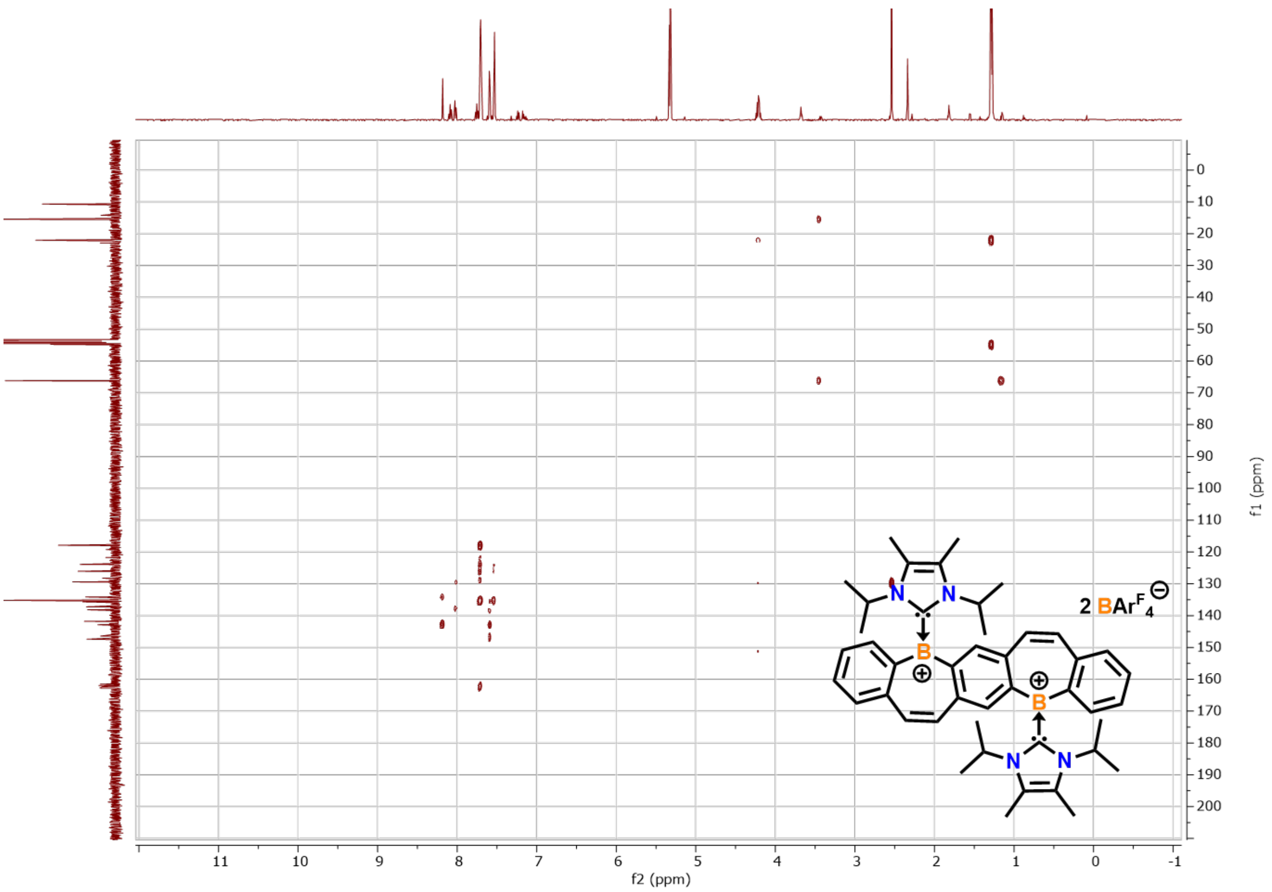

Figure S34. Heteronuclear multiple bond correlation spectroscopy [HMBC] (CD_2Cl_2, 20 °C) spectrum of **7**.

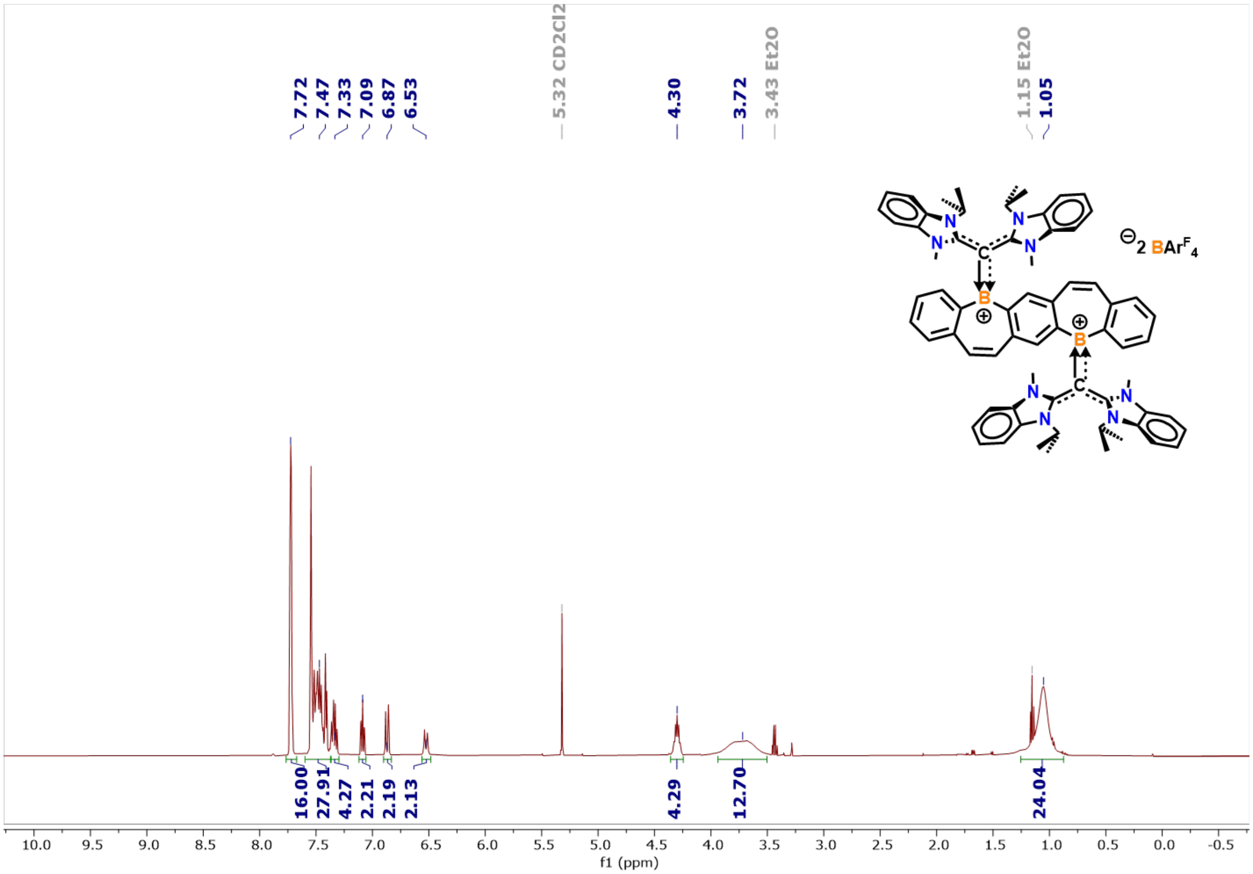

Figure S35. ^{1}H NMR (500 MHz, CD_2Cl_2, 20 °C) spectrum of **8**.

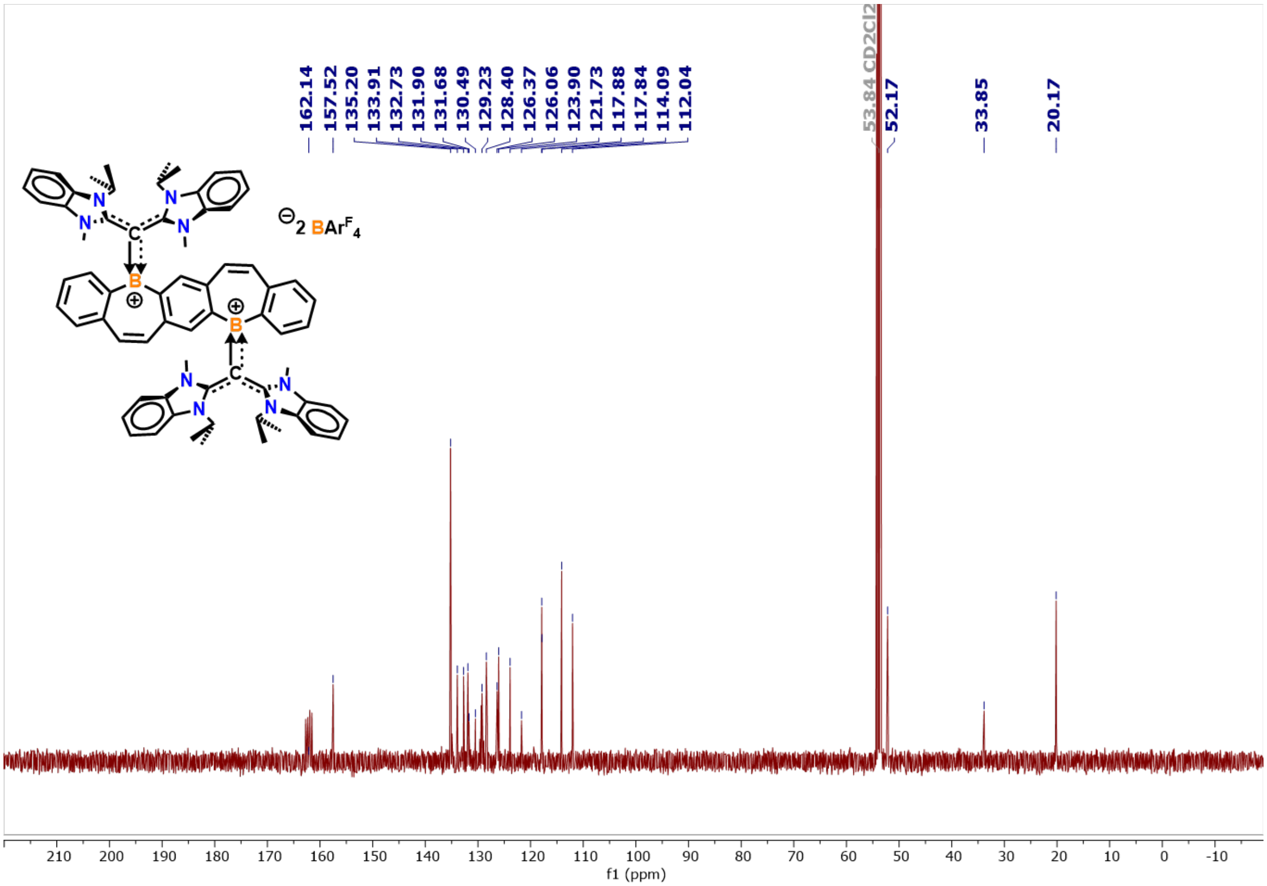

Figure S36. ^{13}C NMR (126 MHz, CD_2Cl_2, 20 °C) spectrum of **8**.

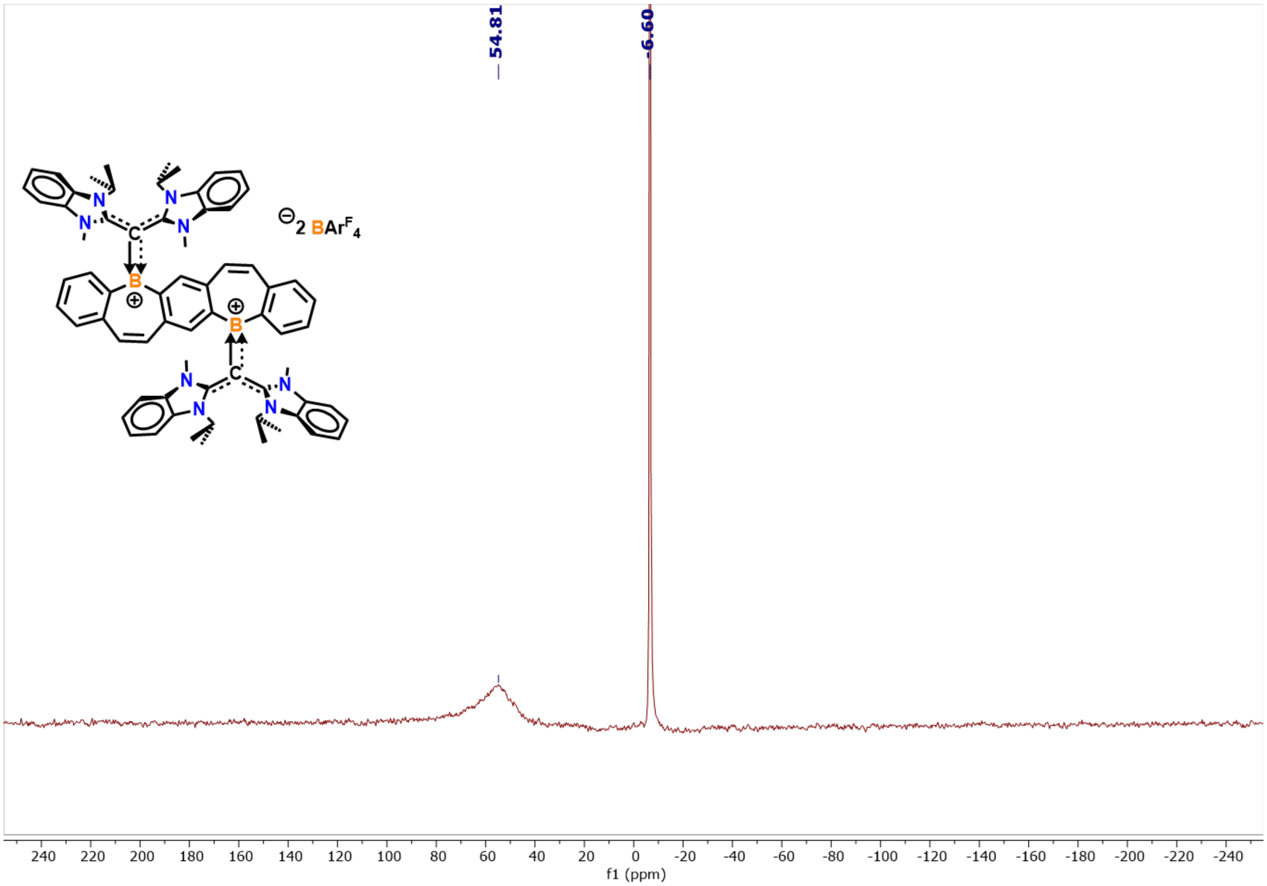

Figure S37. $^{11}B\{^1H\}$ NMR (161 MHz, CD_2Cl_2, 20 °C) spectrum of **8**.

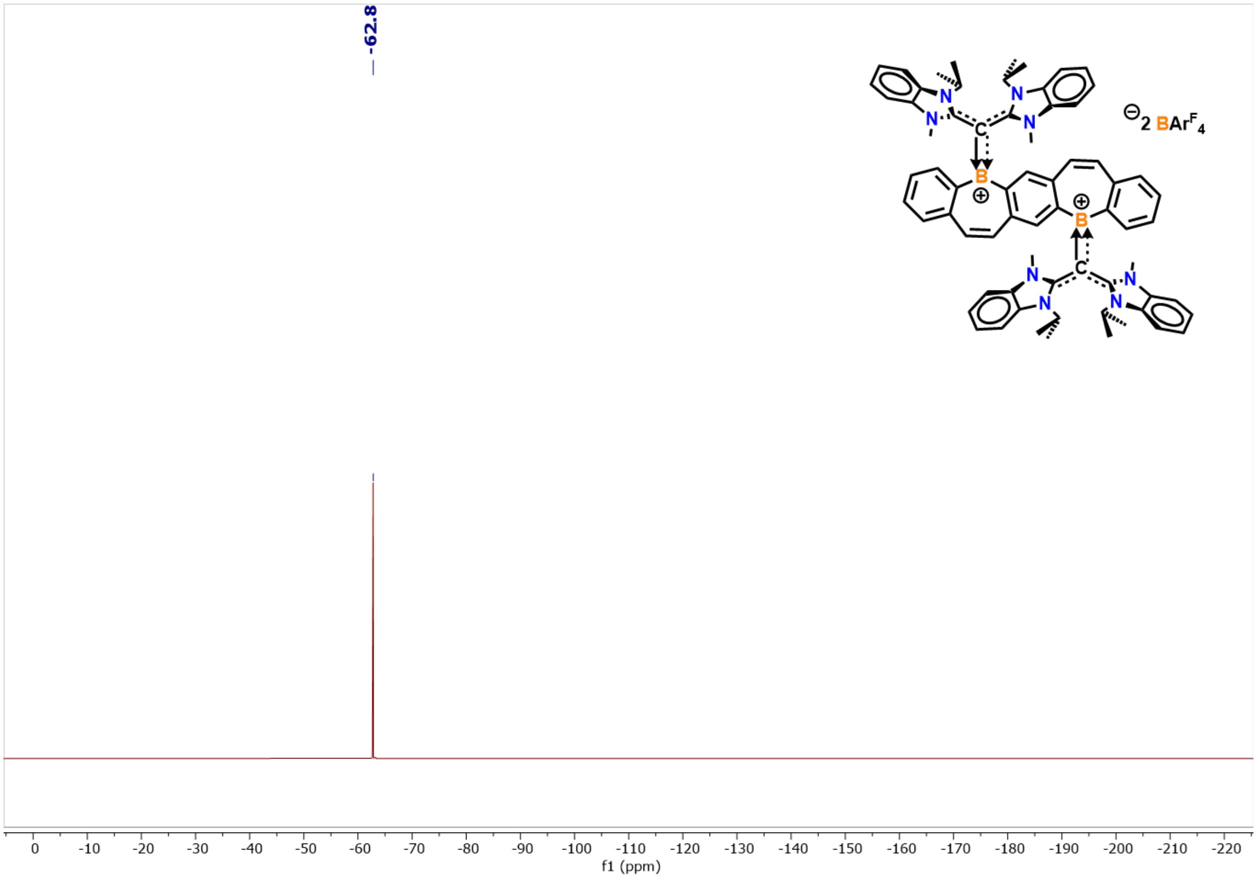

Figure S38. ^{19}F NMR (471 MHz, CD_2Cl_2, 20 °C) spectrum of **8**.

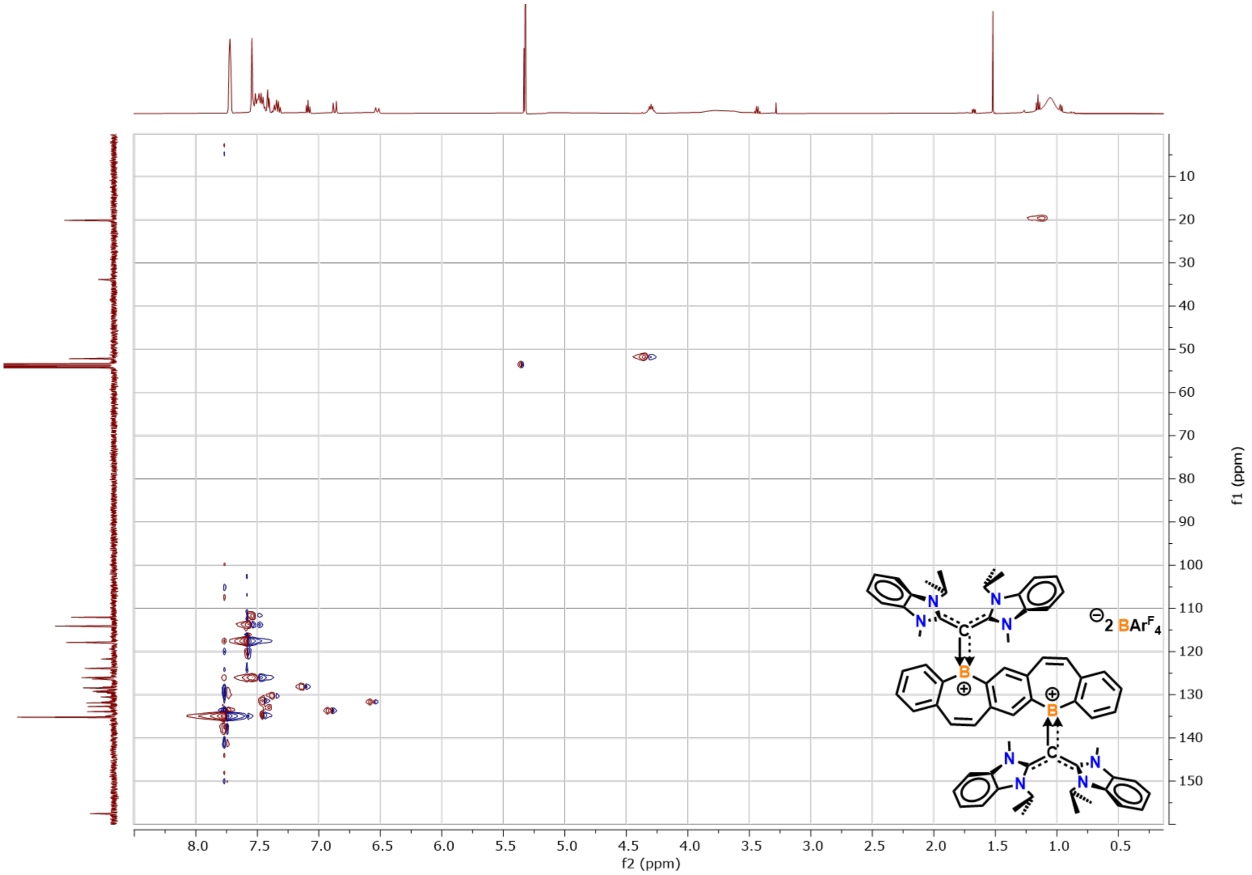

Figure S39. Heteronuclear single quantum coherence spectroscopy [HSQC] (CD_2Cl_2, 20 °C) spectrum of **8**.

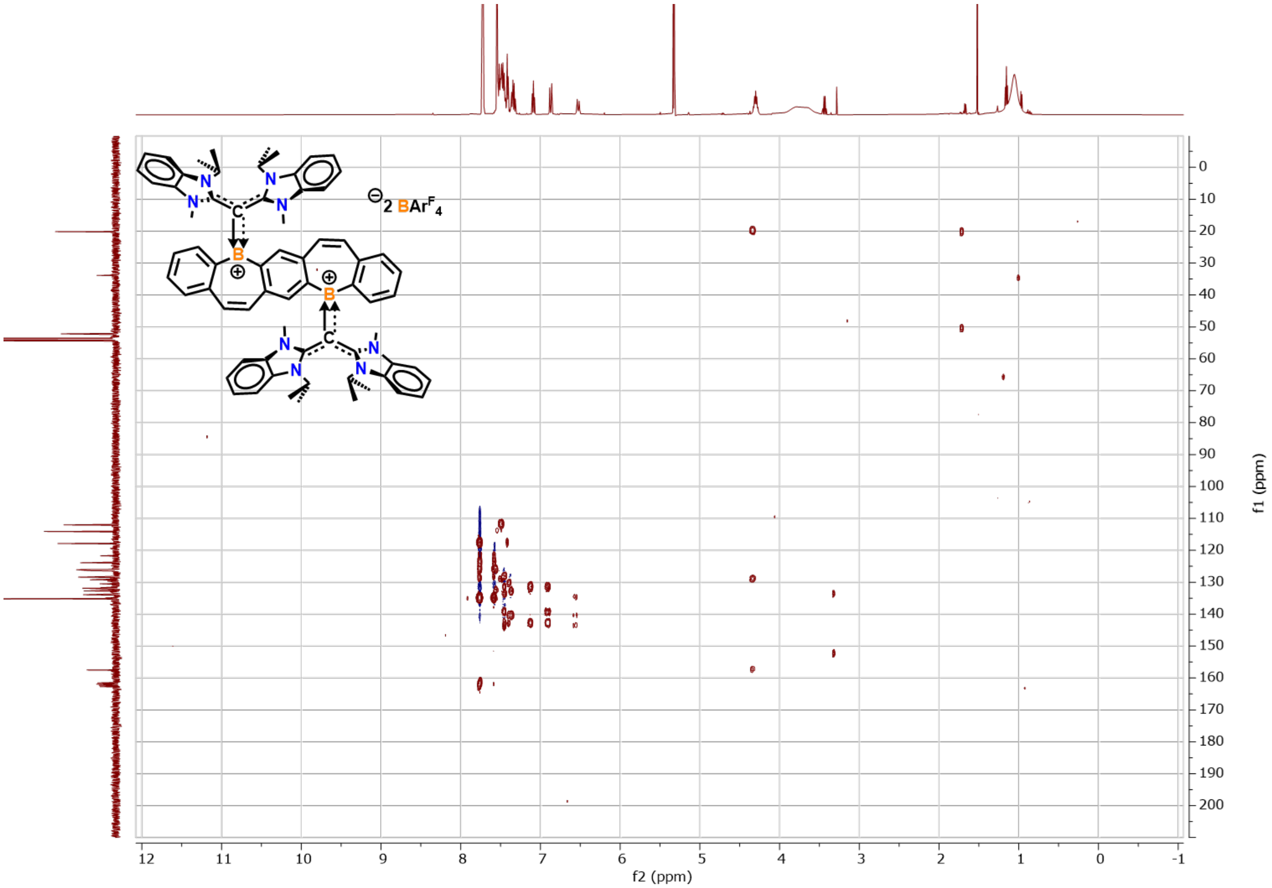

Figure S40. Heteronuclear multiple bond correlation spectroscopy [HMBC] (CD_2Cl_2) spectrum for **8**.

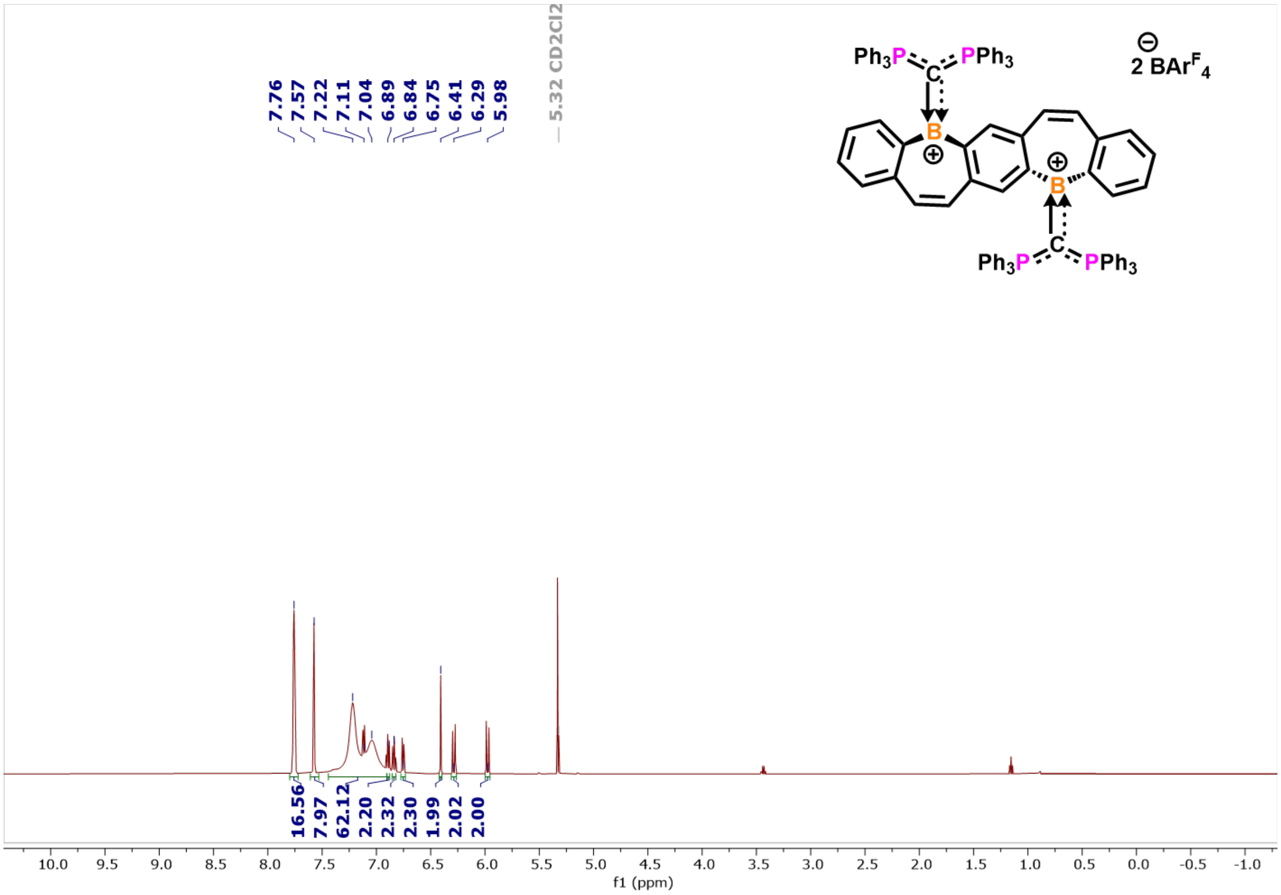

Figure S41. ^{1}H NMR (500 MHz, CD_2Cl_2, 20 °C) spectrum of **9**.

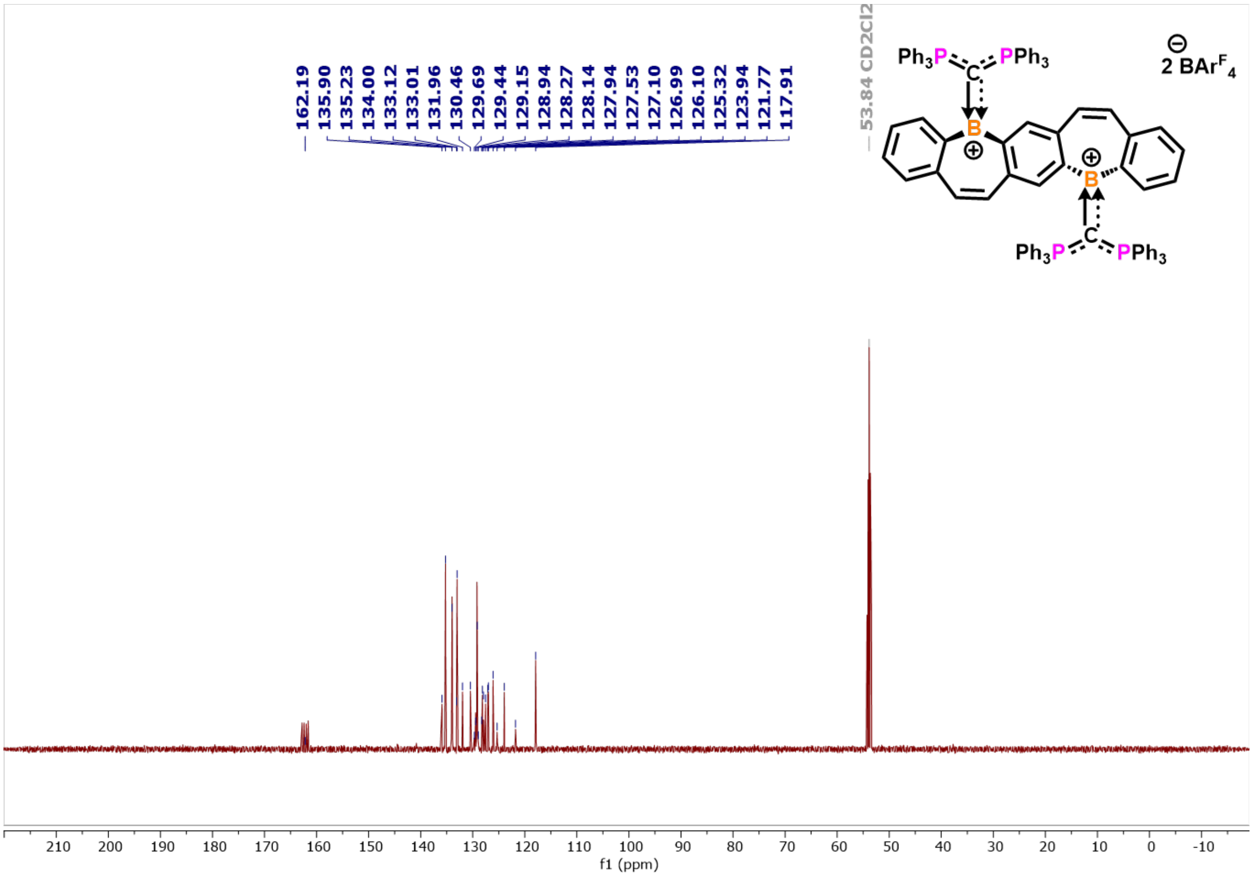

Figure S42. ^{13}C NMR (126 MHz, CD_2Cl_2, 20 °C) spectrum of **9**.

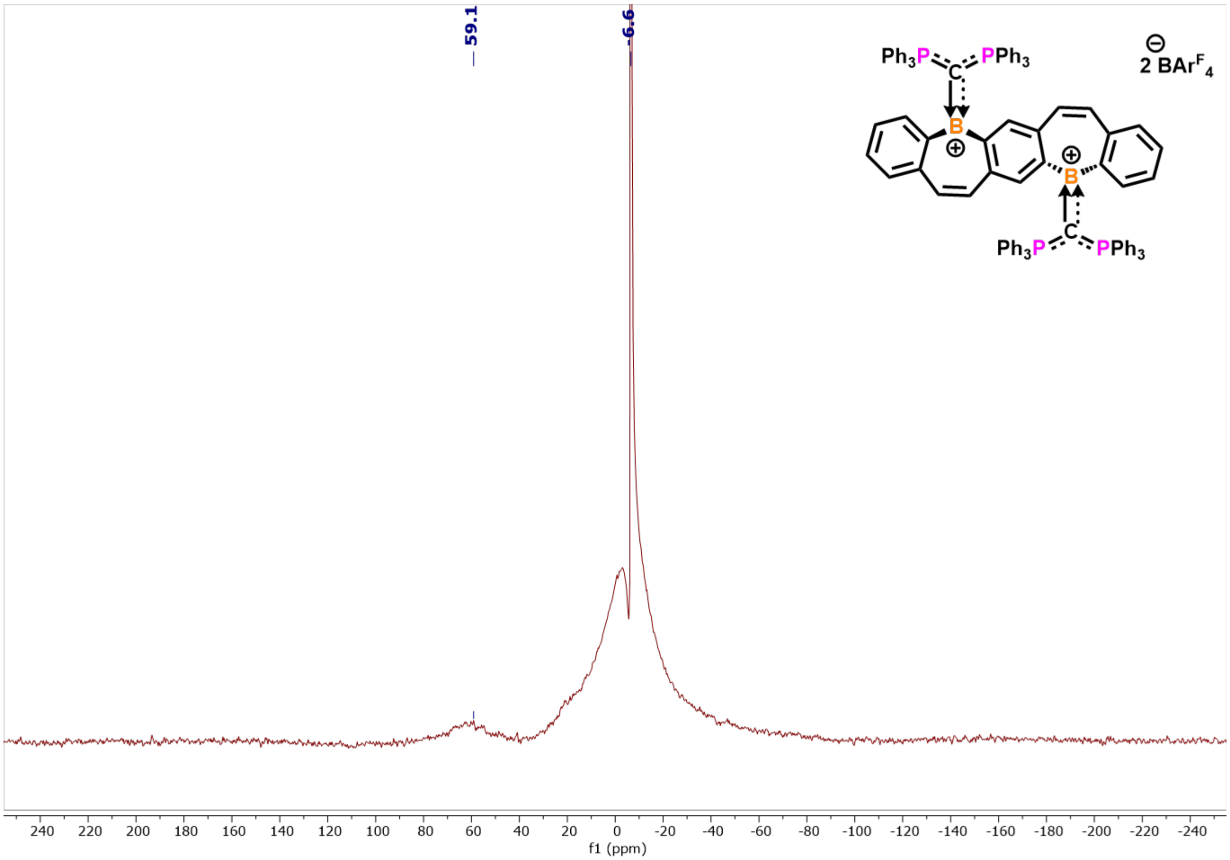

Figure S43. $^{11}B\{^{1}H\}$ NMR (161 MHz, CD_2Cl_2, 20 °C) spectrum of **9**. Borosilicate probe could not be fully processed out due to weak and broad signal at 59.1 ppm.

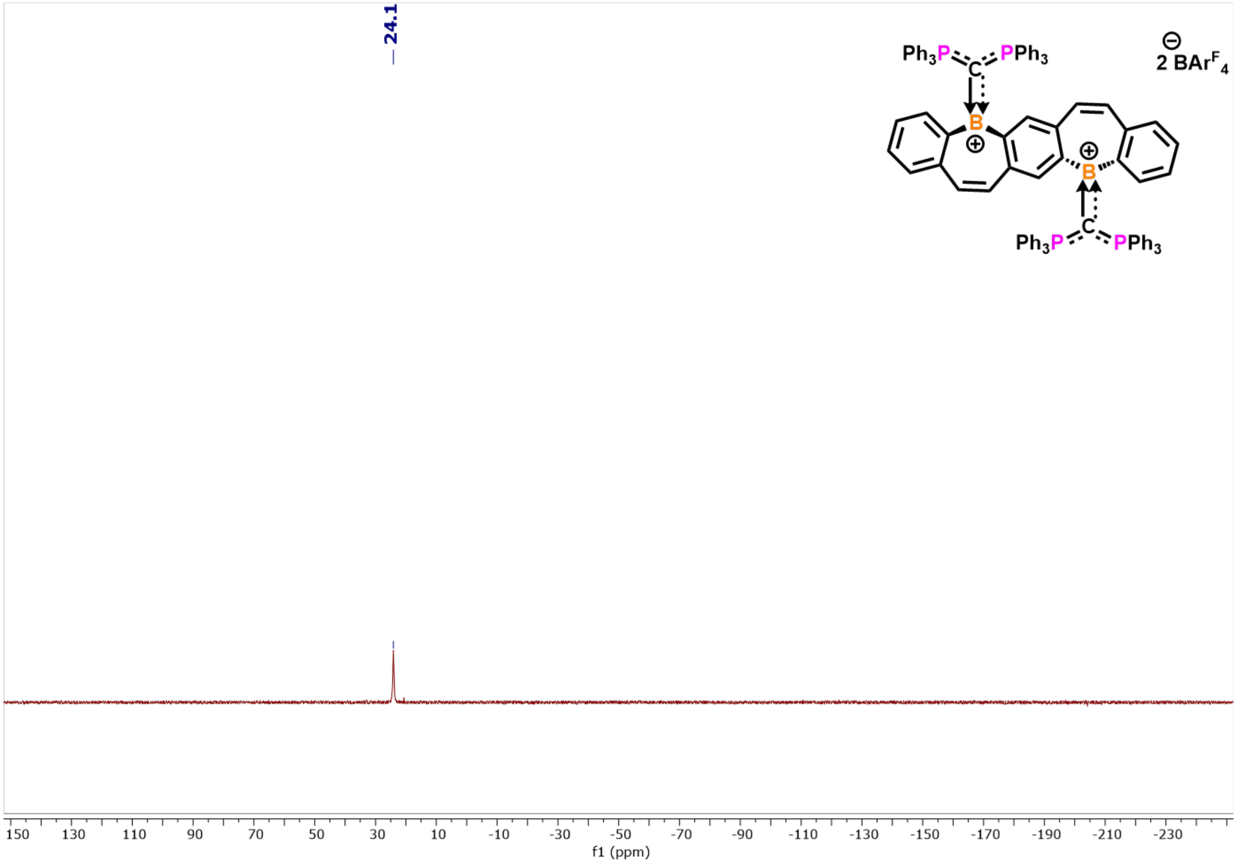

Figure S44. ^{31}P NMR (203 MHz, CD_2Cl_2, 20 °C) spectrum of **9**.

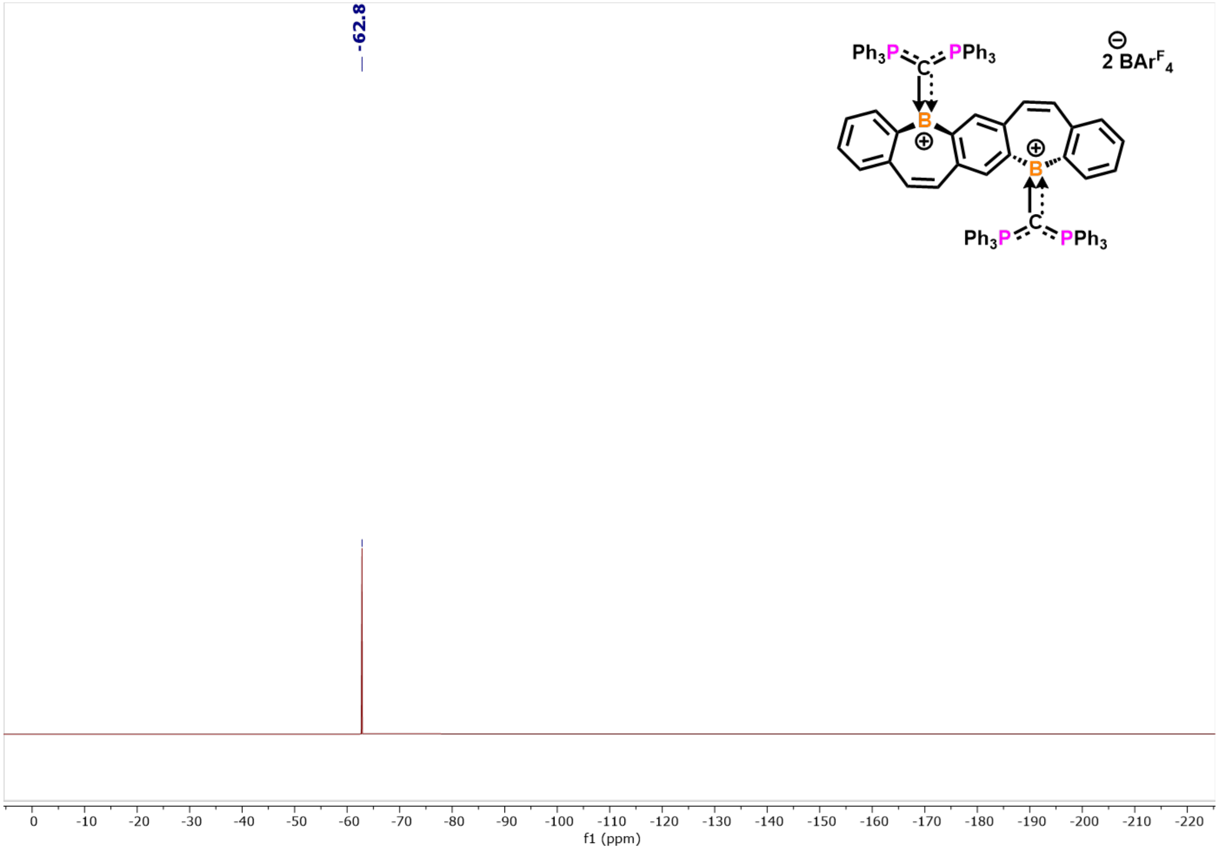

Figure S45. ^{19}F NMR (471 MHz, CD_2Cl_2, 20 °C) spectrum of **9**.

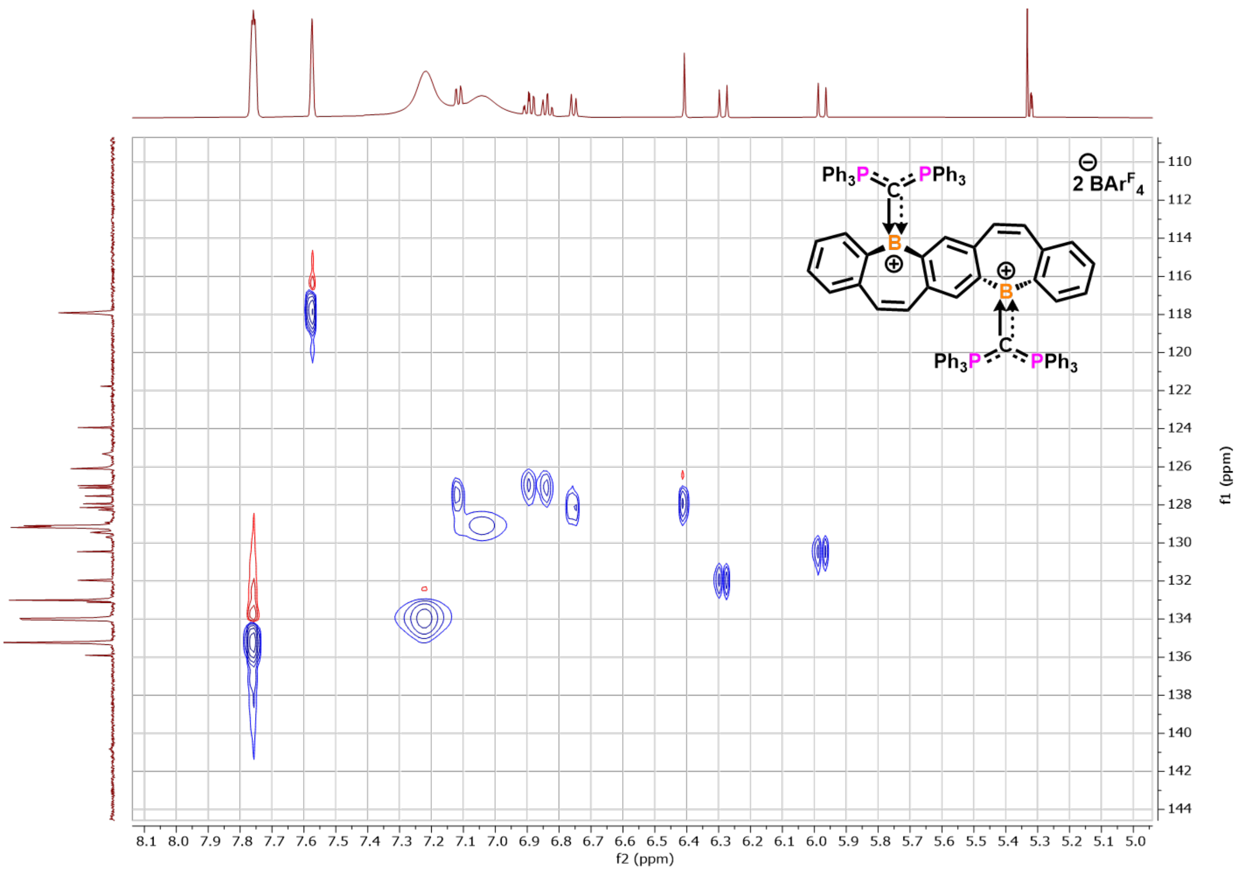

Figure S46. Heteronuclear single quantum coherence spectroscopy [HSQC] (CD_2Cl_2, 20 °C) spectrum of **9**.

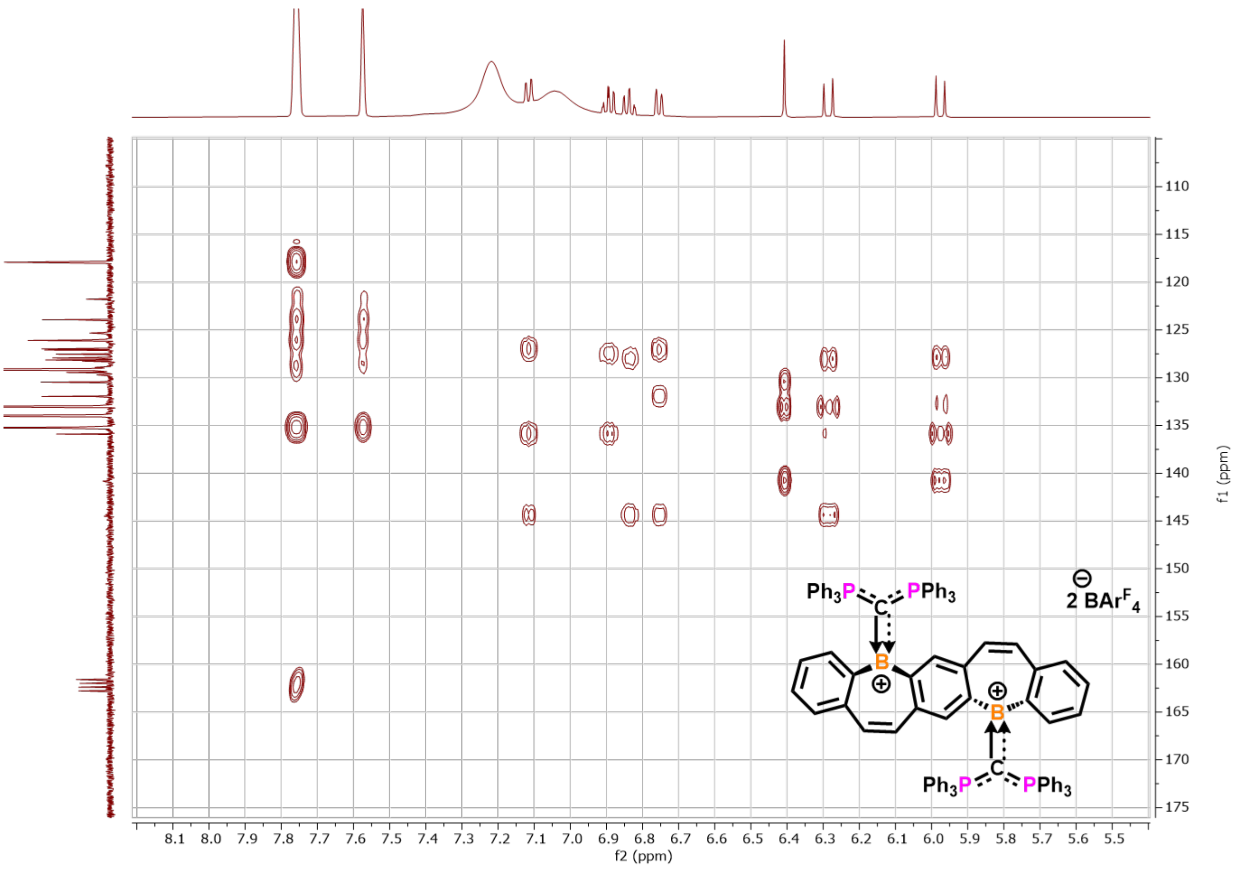

Figure S47. Heteronuclear multiple bond correlation spectroscopy [HMBC] (CD_2Cl_2) spectrum for **9**.

Photophysical Data

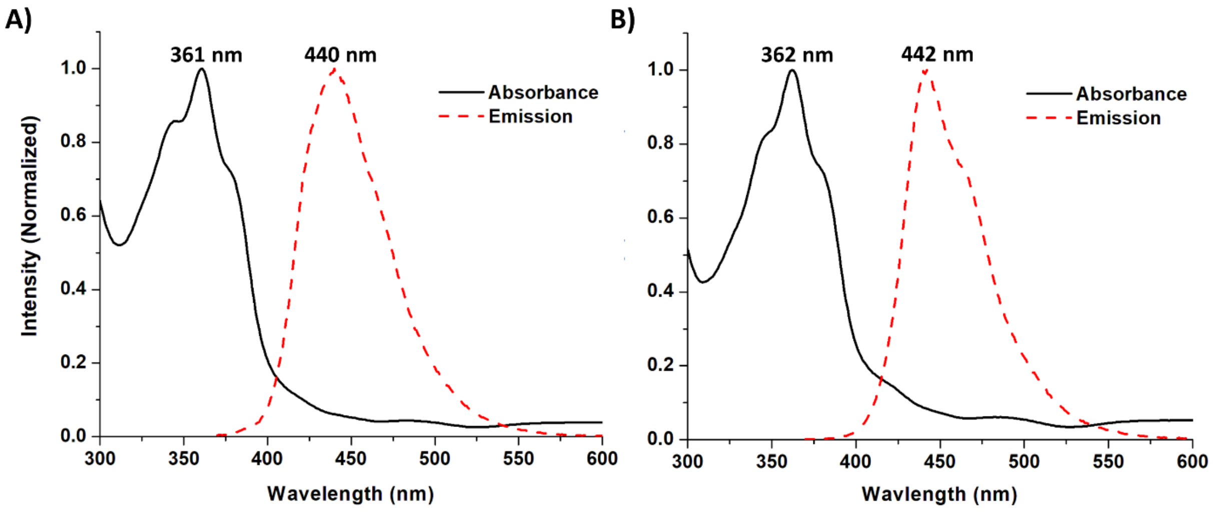

Figure S48. Normalized UV/vis (black trace) and fluorescence (red trace) spectra of **1-Br** (A) and **1-Cl** (B) in chlorobenzene. λ_{ex} = 360 nm.

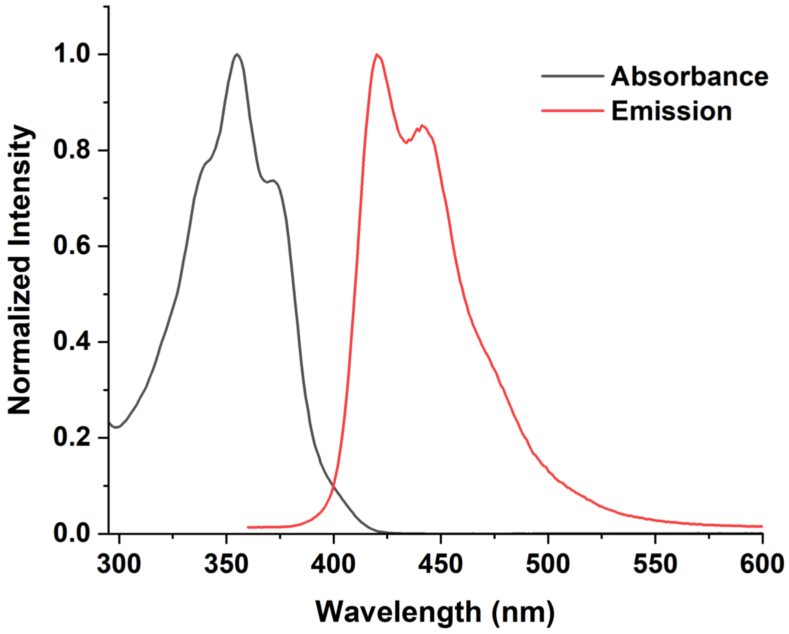

Figure S49. Dilute absorbance and fluorescence spectrum of decomposed **5** in o-DFB (λ_{ex} = 350 nm, [0.007 mM]). Note: The concentrated sample of **5** in o-DFB is reported in Figure 5B (emission peak at 561 nm).

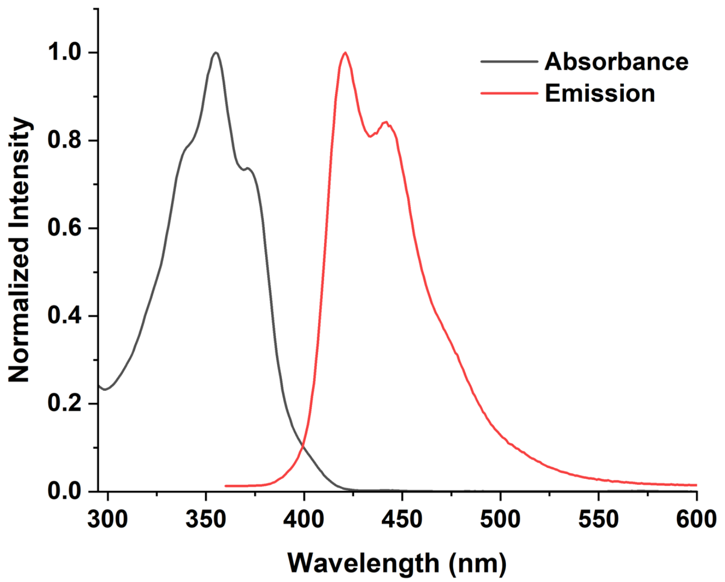

Figure S50. Dilute absorbance and fluorescence spectra of decomposed **6** in o-DFB (λ_{ex} = 350 nm, [0.005 mM]). Note: The concentrated sample of **6** in o-DFB is reported in Figure 5B (emission peak at 575 nm).

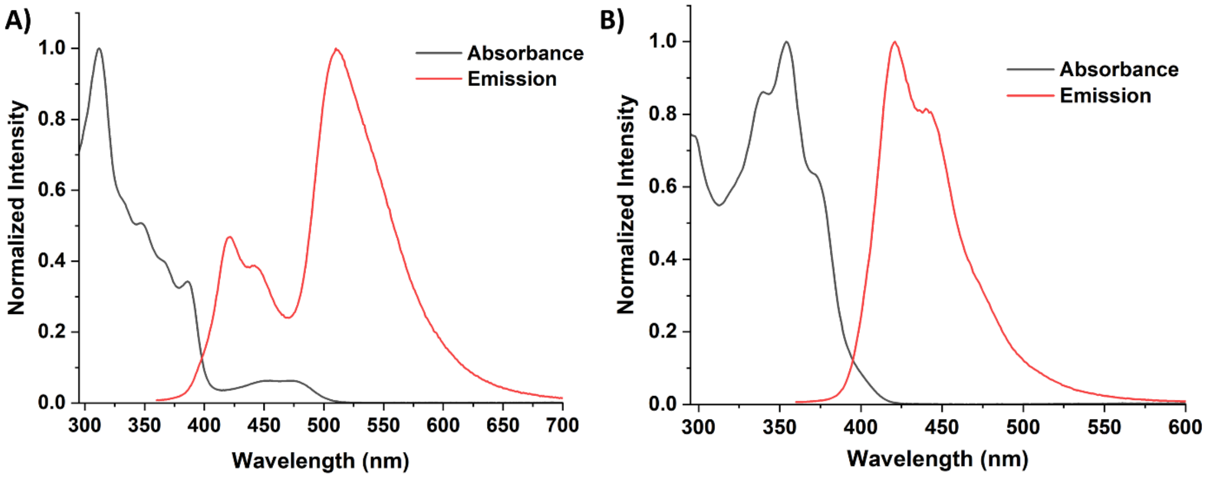

Figure S51. Dilute absorbance and fluorescence spectra of decomposed **7** in o-DFB (λ_{ex} = 350 nm, [0.001 mM]). A) Spectrum taken ten minutes after sample was prepared. B) Spectrum taken three hours after sample was prepared. Note: Compound **7** decomposes at lower concentrations and more slowly than **5** and **6**. Compound **7** is fully decomposed after three hours and looks similar to **5** and **6**.

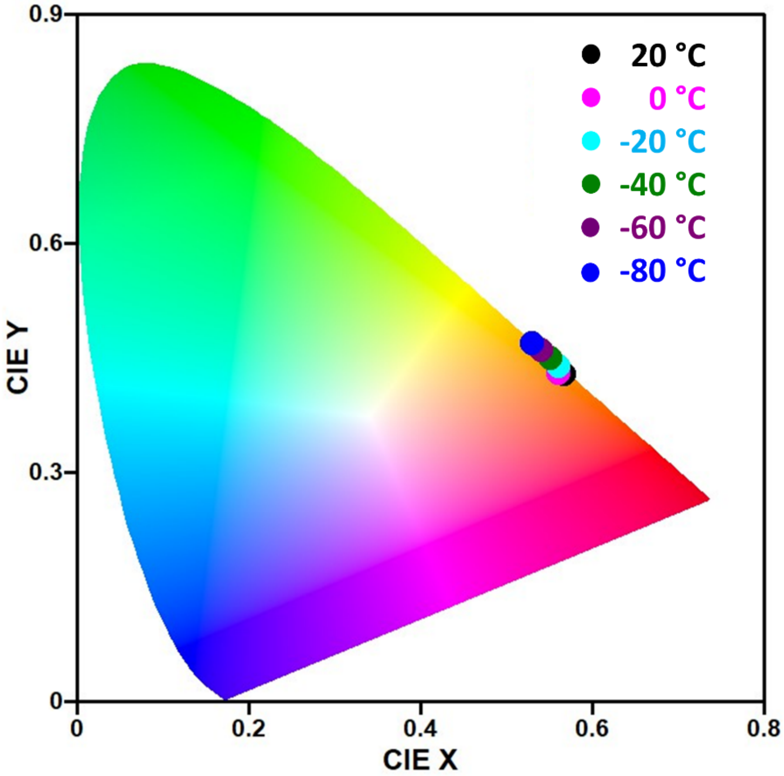

Figure S52. CIE (1931) chromaticity diagram of VT fluorescence of **8** in DCM (λ_{ex} = 480 nm, [0.01 mM]).

Thermogravimetric Analysis (TGA) Data

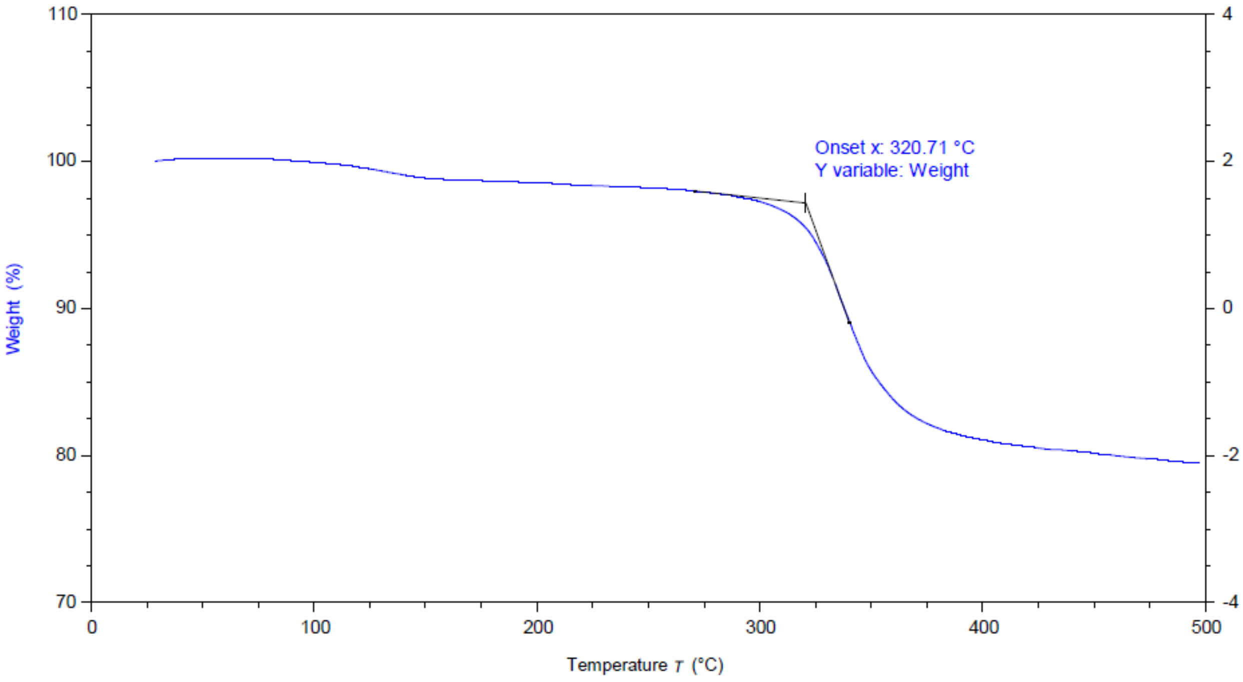

Figure S53. TGA spectrum of **1-Br**. *Small dip just above 100 °C is due to loss of toluene.

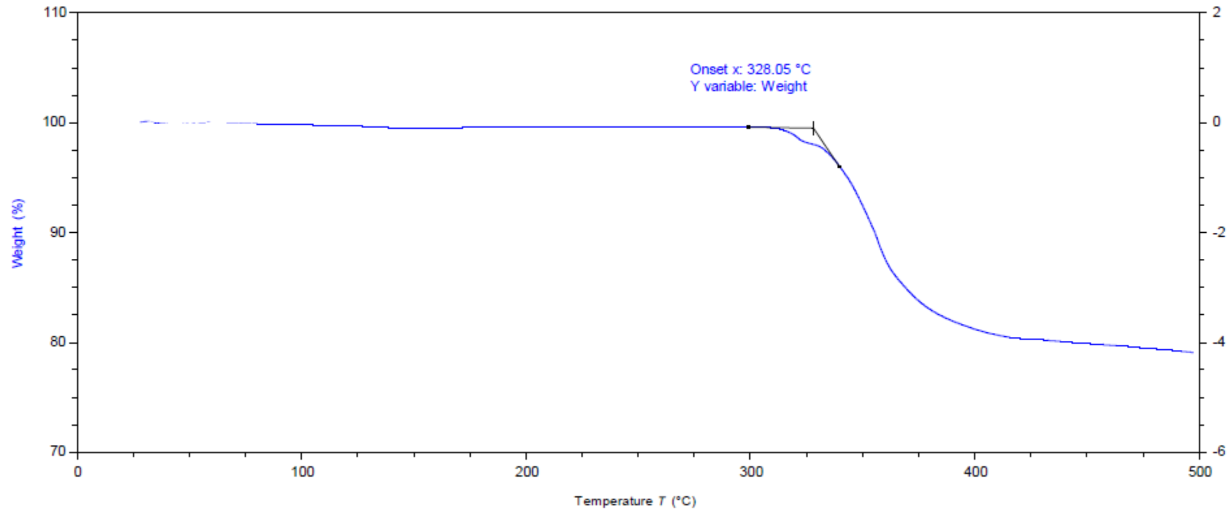

Figure S54. TGA spectrum of **1-Cl**.

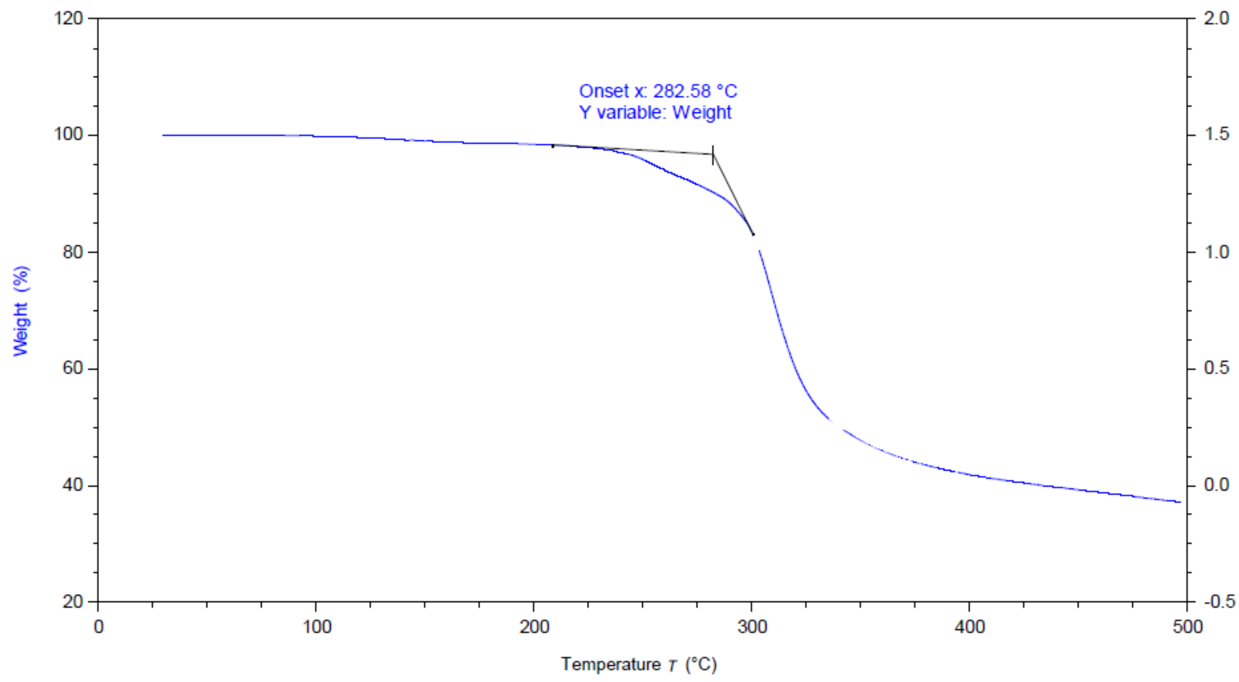

Figure S55. TGA spectrum of **5**.

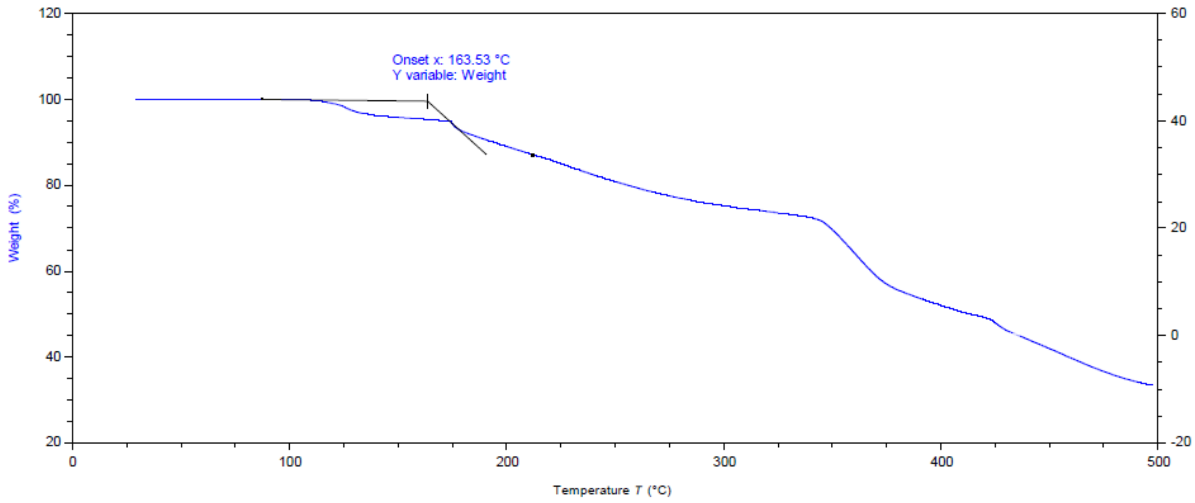

Figure S56. TGA spectrum of **6**.

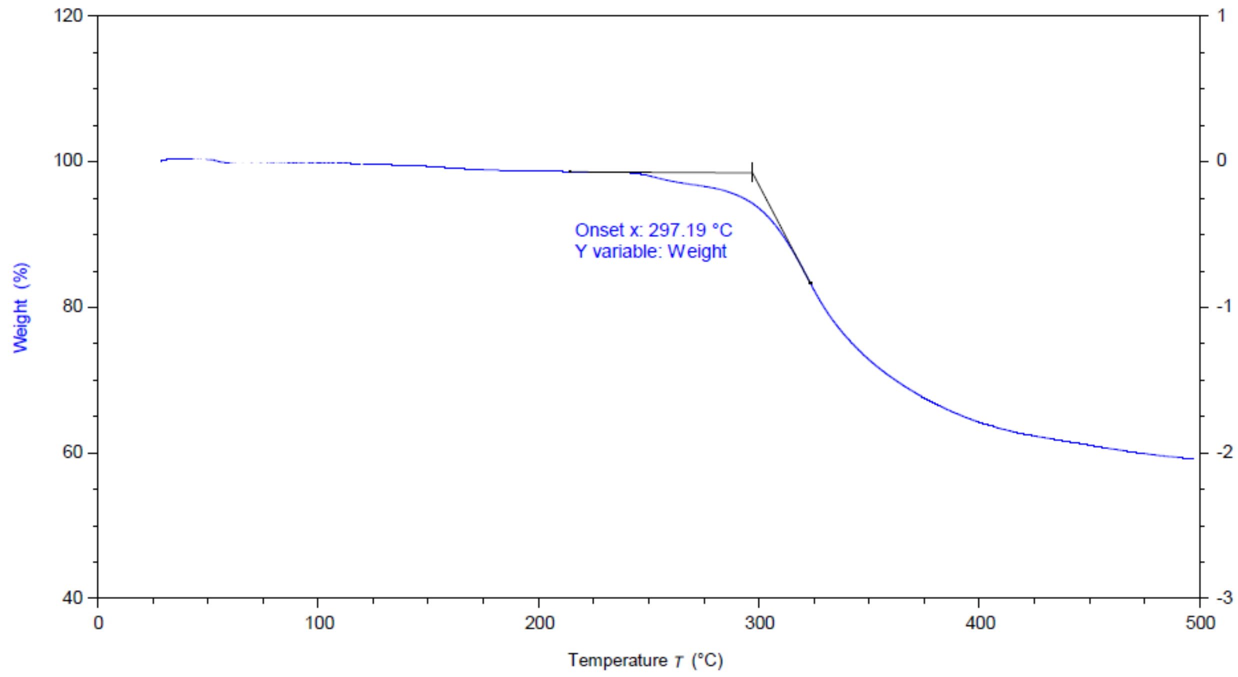

Figure S57. TGA spectrum of **7**.

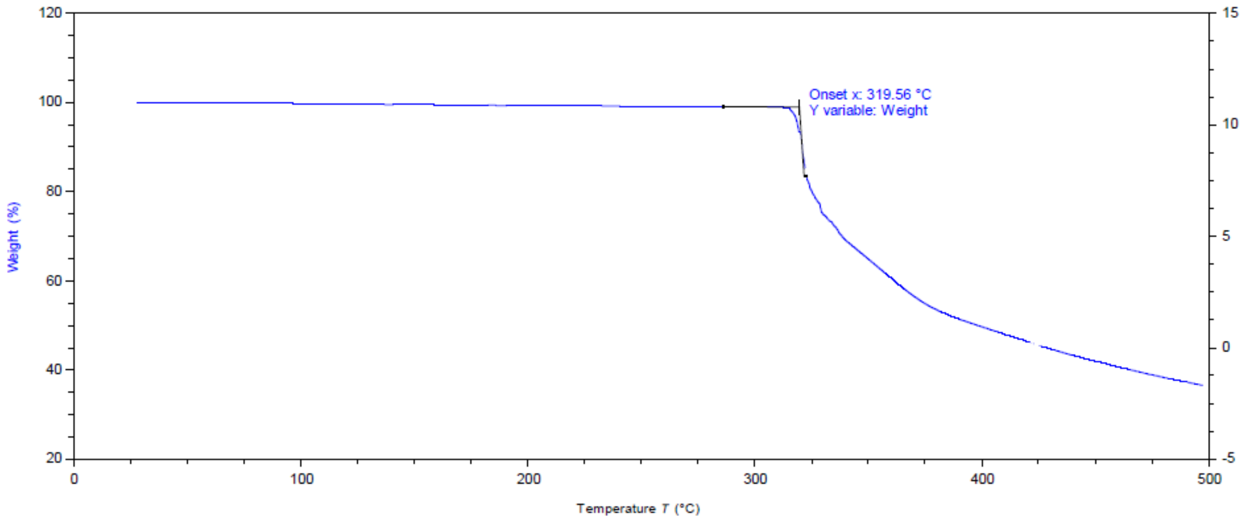

Figure S58. TGA spectrum of **8**.

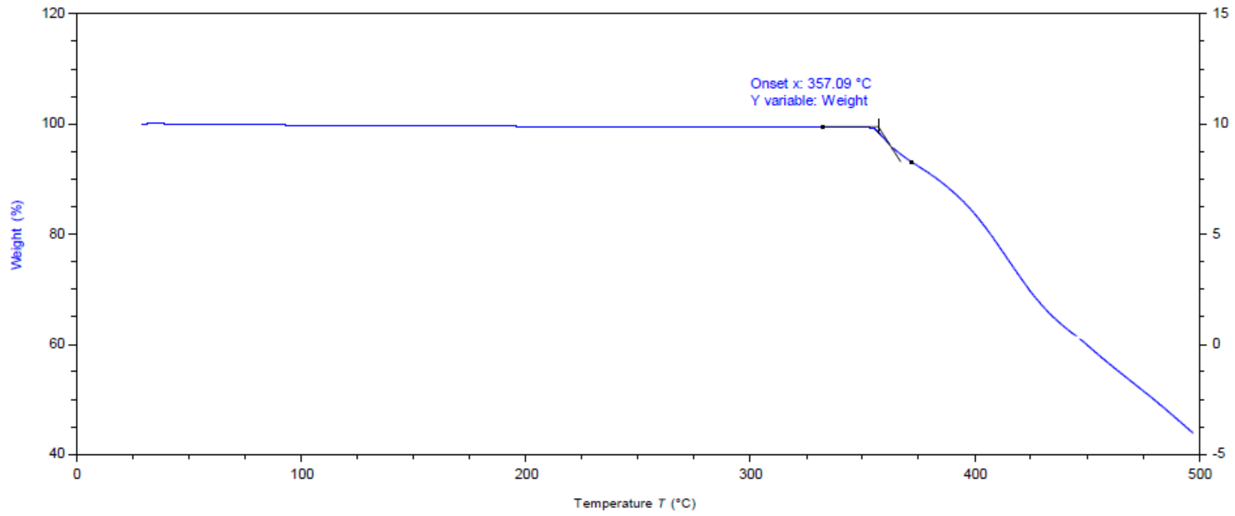

Figure S59. TGA spectrum of **9**.

X-ray Crystallography Details

Low temperature single crystal X-ray diffraction data for **1-Cl, 3-Cl, 4-Cl, 5** and **6** were collected on a Bruker Kappa APEXII CCD system equipped with a fine-focus sealed tube (Mo K_α, λ = 0.71073 Å) and a graphite monochromator. Low temperature single crystal X-ray diffraction data for **2-Cl**, **7**, **S5**, **S6**, and **S7** were collected on a Bruker D8 Venture PhotonIII Kappa four-circle diffractometer system equipped with dual Incoatec IμS 3.0 micro-focus sealed X-ray tubes (Cu Kα, λ = 1.54178 Å; Mo Kα, λ = 0.71073 Å) and HELIOS double bounce multilayer mirror monochromators. Low-temperature diffraction data for **8** and **9** were collected on a Bruker-AXS Kappa Duo diffractometer equipped with *IμS* micro-sources, coupled to a Photon 3 CPAD detector, performing ϕ-and ω-scans, and using Mo K_α radiation (λ = 0.71073 Å). Data reduction was carried out with the program SAINT[10] and semi-empirical absorption correction based on equivalents was performed with the program SADABS.[11] All structures were solved by dual-space methods using SHELXT[12] and refined against F^2 on all data by full-matrix least squares with SHELXL[13] following established refinement strategies.[14] Details about data quality and a summary of the residual values of the refinement are listed in Tables 1 and 2.

The structures **1-9** and **S5-S7** were solved and refined using the Bruker SHELXTL Software Package[12] within OLEX2 1.5.[15] The frames were integrated with the Bruker SAINT software package[10] using a narrow-frame algorithm. Data were corrected for absorption effects using the Multi-Scan method (SADABS).[11] Non-hydrogen atoms were refined anisotropically. Hydrogen atoms were placed in geometrically calculated positions with $U_{iso} = 1.2U_{equiv}$ of the parent atom ($1.5U_{equiv}$ for methyl). The relative occupancies of each position of the disordered sites were freely refined, unless the disorder was symmetry related. In those cases, the occupancy was fixed at 50%. Constraints and restraints were used on the anisotropic displacement parameters and bond lengths of most of the disordered atoms.

Compound **8** crystallizes in the triclinic centrosymmetric space group *P*-1 with one half molecule of **8**, one BAr^F_4 anion, and one molecule of diethyl ether in the asymmetric unit. The second half of **8** is generated by the crystallographic inversion center. The ether molecule was refined as completely disordered over two positions and six of the eight CF_3 groups on the BARF ion were also refined as disordered over two or, in one case, even three positions. All disorders were refined with the help of similarity restraints on 1-2 and 1-3 distances as well as similarity restraints on displacement parameters and advanced rigid-bond restraints.

Compound **9** crystallizes in the triclinic centrosymmetric space group *P*-1 with one half molecule of **9**, one BAr^F_4 anion, and 1.5 molecules of dichloromethane in the asymmetric unit. The second half of **9** is generated by the crystallographic inversion center. The half occupied solvent molecule resides near an inversion center and is, therefore, disordered about that symmetry element. In addition, two independent positions could be established for that CH_2Cl_2 molecule, resulting in an affective four-component disorder (although only two of them are crystallographically unique). Furthermore, four of the eight CF_3 groups on the BAr^F_4 ion were refined as disordered over two

positions. All disorders were refined with the help of similarity restraints on 1-2 and 1-3 distances as well as similarity restraints on displacement parameters and advanced rigid-bond restraints.

Table S1: Crystallographic data for compounds 1-4 (Cl), 5-6

	**1-Cl**	**2-Cl**	**3-Cl**	**4-Cl**	**5**	**6**
CCDC number	2344133	2344134	2344135	2344136	2344137	2344138
Formula	$C_{22}H_{14}B_2Cl_2$	$C_{41}H_{58}BClNO_2$	$C_{159}H_{180}B_4Cl_4N_8$	$C_{44}H_{54}B_2Cl_2N_4$	$C_{65}H_{54}B_2F_{24}N$	$C_{142}H_{114}B_4Cl_4F_{48}N_4$
FW (g/mol)	370.85	643.14	2388.14	731.43	1326.99	2973.88
Temp (K)	100.01	100.0	99.99	99.98	100.03	100(2)
λ (Å)	0.71073	0.71073	1.54178	0.71073	0.71073	0.71073
Size (mm)	0.373 × 0.053 × 0.052	0.453 × 0.426 × 0.202	0.145 × 0.076 × 0.046	0.611 × 0.226 × 0.198	0.3 × 0.28 × 0.12	0.127 x 0.196 x 0.337
Crystal habit	orange needle	yellow block	colorless rod	colorless block	orange plate	red block
Crystal system	monoclinic	monoclinic	triclinic	monoclinic	triclinic	monoclinic
Space group	$P2_1/c$	C2/c	P-1	$P2_1/n$	P-1	$P\ 2_1/n$
a (Å)	13.331(4)	24.7297(12)	10.1202(5)	11.2159(11)	12.5802(15)	13.601(3)
b(Å)	3.9432(11)	18.7599(8)	17.0454(8)	11.9173(12)	13.1420(16)	23.955(6)
c (Å)	16.631(5)	15.6595(7)	19.6957(11)	15.0102(16)	19.744(2)	21.060(4)
α (°)	90	90	95.587(4)	90	85.418(2)	90
β (°)	108.334(7)	97.779(2)	96.221(4)	90.326(3)	79.658(2)	96.006(6)
γ (°)	90	90	90.244(4)	90	73.127(2)	90
Volume ($Å^3$)	829.9(4)	7198.0(6)	3361.1(3)	2006.3(4)	3071.6(6)	6824.(2)
Z	2	8	1	2	2	2
Density (g/cm^3)	1.484	1.187	1.180	1.211	1.435	1.447
μ (mm^{-1})	0.393	0.142	1.218	0.198	0.134	0.205
F(000)	380.0	2792.0	1278.0	780.0	1354.0	3028
θ range (°)	1.609 to 25.357	1.972 to 33.788	2.267 to 68.602	2.182 to 31.015	1.62 to 33.751	1.70 to 25.39
Index ranges	$-16 \le h \le 16$<br>$-4 \le k \le 4$<br>$-20 \le l \le 20$	$-38 \le h \le 38$<br>$-28 \le k \le 29$<br>$-21 \le l \le 24$	$-12 \le h \le 11$<br>$-20 \le k \le 20$<br>$-23 \le l \le 23$	$-16 \le h \le 16$<br>$-17 \le k \le 16$<br>$-21 \le l \le 21$	$-19 \le h \le 19$<br>$-20 \le k \le 20$<br>$-30 \le l \le 30$	$-16 \le h \le 16$<br>$-28 \le k \le 28$<br>$-25 \le l \le 25$
Reflns collected	8643	83023	49634	27359	119222	77462
Independent reflns	1523 [R_{int} = 0.1091]	14377 [R_{int} = 0.0582]	12273 [R_{int} = 0.1088]	6397 [R_{int} = 0.0350]	24545 [R_{int} = 0.0452]	12495 [R_{int} = 0.1018]
Data / restraints /parameters	1523/0/118	14377/0/444	12273/0/877	6397/0/241	24545/187/938	12495/75/985
GOF on F^2	1.018	1.050	1.008	1.028	1.021	1.008
R_1 (I>2σ(I))	0.0501	0.0611	0.0561	0.0386	0.0516	0.0640
wR_2 (all data)	0.1385	0.1918	0.1507	0.1038	0.1440	0.1430

Table S2: Crystallographic data for compounds 7-9, S5-S7

	**7**	**8**	**9**	**S5**	**S6**	**S7**
CCDC number	2344139	2344145	2344146	2344140	2344141	2344142
Formula	$C_{110}H_{82}B_4Cl_4F_{48}N_4$	$C_{140}H_{114}B_4F_8N_8O_2$	$C_{166}H_{102}B_4Cl_4F_{48}P_4$	$C_{72}H_{88}B_2Cl_4F_{12}N_4O$	$C_{84}H_{94}B_2Cl_8F_{12}N_6O_8S_4$	$C_{50}H_{58}B_2Cl_4F_{12}N_8O_8S_4$
FW (g/mol)	2555.88	2895.63	3317.585	1657.12	1976.41	1418.70
Temp (K)	100.(2)	100(2)	100.15	100.00	100(2)	100.00
λ (Å)	0.71073	0.71073	0.71073	1.54178	0.71073	0.71073
Size (mm)	0.455 × 0.304 × 0.283	0.320 x 0.100 x 0.065	0.665 × 0.445 × 0.195	0.172 × 0.06 × 0.029	0.126 x 0.190 x 0.236	0.504 × 0.235 × 0.18
Crystal habit	orange block	orange plate	colorless plate	pink needle	orange block	orange block
Crystal system	triclinic	triclinic	triclinic	monoclinic	monoclinic	triclinic
Space group	P-1	P-1	P-1	$P2_1/c$	$P\ 2_1/n$	P-1
a (Å)	12.3947(8)	11.0428(5)	13.2079(5)	14.9640(6)	16.7454(5)	8.9168(4)
b(Å)	12.5964(8)	16.5514(8)	15.4895(6)	11.9337(5)	27.4896(9)	9.3168(4)
c (Å)	19.1553(12)	19.2623(8)	19.1541(6)	21.9364(9)	21.7003(6)	18.6398(7)
α (°)	79.135(2)	77.797(2)	80.403(1)	90	90	96.7530(10)
β (°)	72.923(2)	84.084(2)	84.517(1)	101.645(3)	112.1310(10)	92.3660(10)
γ (°)	89.308(2)	73.633(2)	72.088(1)	90	90	95.8310(10)
Volume (Å^3)	2804.6(3)	3298.1(3)	3672.4(2)	3836.7(3)	9253.2(5)	1527.60(11)
Z	1	1	1	2	4	1
Density (g/cm^3)	1.513	1.458	1.500	1.434	1.419	1.542
μ (mm^{-1})	0.235	0.133	0.240	3.157	0.414	0.425
F(000)	1290.0	1478	1680.5	1724.0	4087	728.0
θ range (°)	2.17 to 29.15	1.924 to 30.521	1.88 to 31.52	3.015 to 68.244	1.94 to 26.40	2.214 to 27.109
Index ranges	$-16 \le h \le 16$ $-17 \le k \le 17$ $-26 \le l \le 25$	$-15 \le h \le 15$ $-23 \le k \le 23$ $-22 \le l \le 27$	$-19 \le h \le 19$ $-22 \le k \le 22$ $-28 \le l \le 26$	$-18 \le h \le 18$, $-14 \le k \le 13$, $-23 \le l \le 26$	$-20 \le h \le 20$, $-34 \le k \le 34$, $-27 \le l \le 27$	$-11 \le h \le 11$, $-11 \le k \le 11$, $-23 \le l \le 22$
Reflns collected	85598	56332	222447	29511	155109	40018
Independent reflns	15059 [R_{int} = 0.0394]	19780 [R_{int} = 0.0627]	24400 [R_{int} = 0.0360]	6972 [R_{int} = 0.1116]	18917 [R_{int} = 0.0899]	6729 [R_{int} = 0.0315]
Data / restraints /parameters	15059/123/995	19780 / 3917 / 1180	24400/204/1167	6972/0/486	18917 / 340 / 1426	6729/0/489
GOF on F^2	1.035	1.021	1.042	1.023	1.030	2.583
R_1 ($I>2\sigma(I)$)	0.0529	0.0636	0.0473	0.0625	0.0677	0.0809
wR_2 (all data)	0.1492	0.1491	0.1342	0.1706	0.1908	0.2909

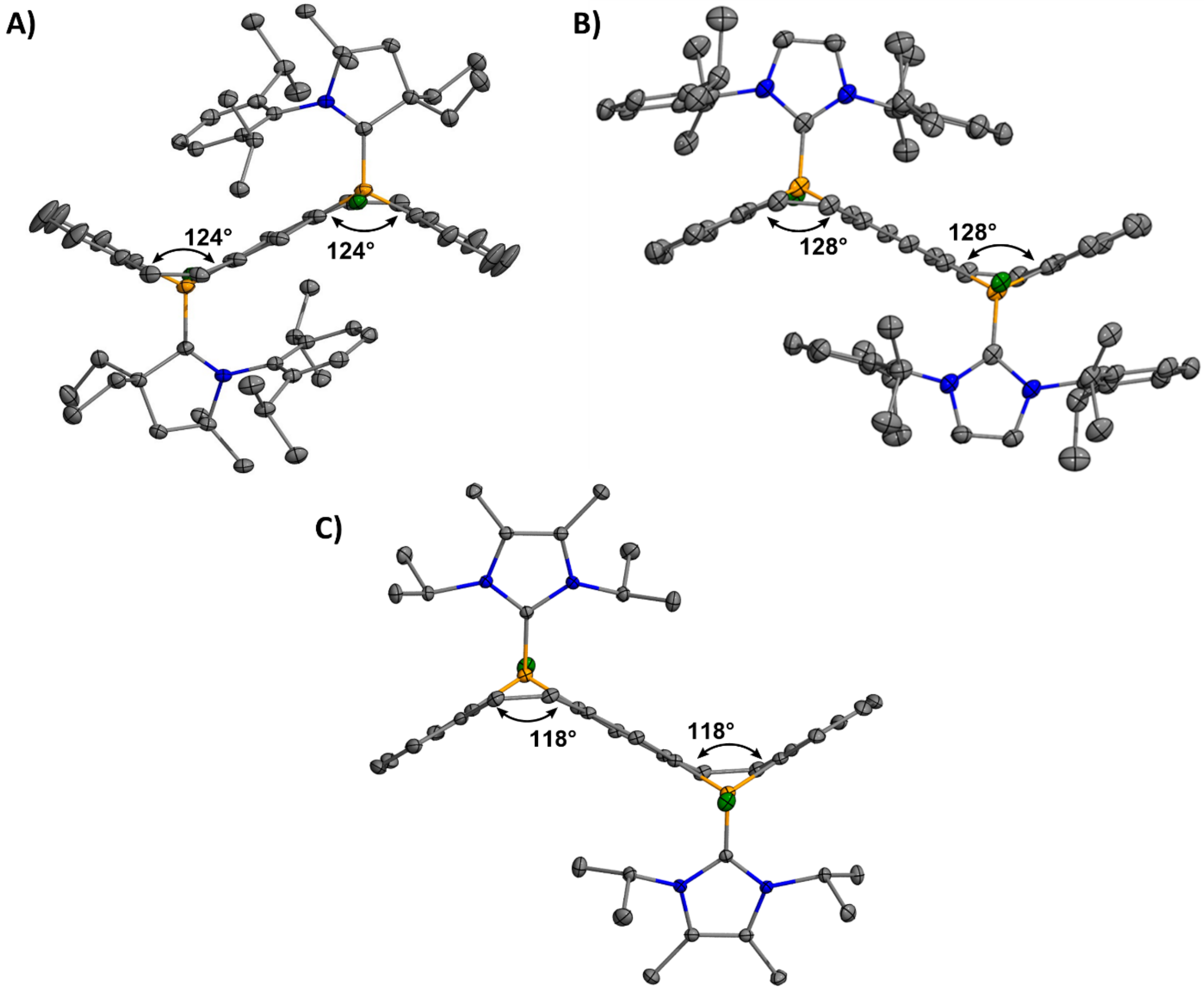

Figure S60. Side view of tetracoordinate FBP adducts **2-Cl** (A), **3-Cl** (B), and **4-Cl** (C), displaying distortion from planarity.

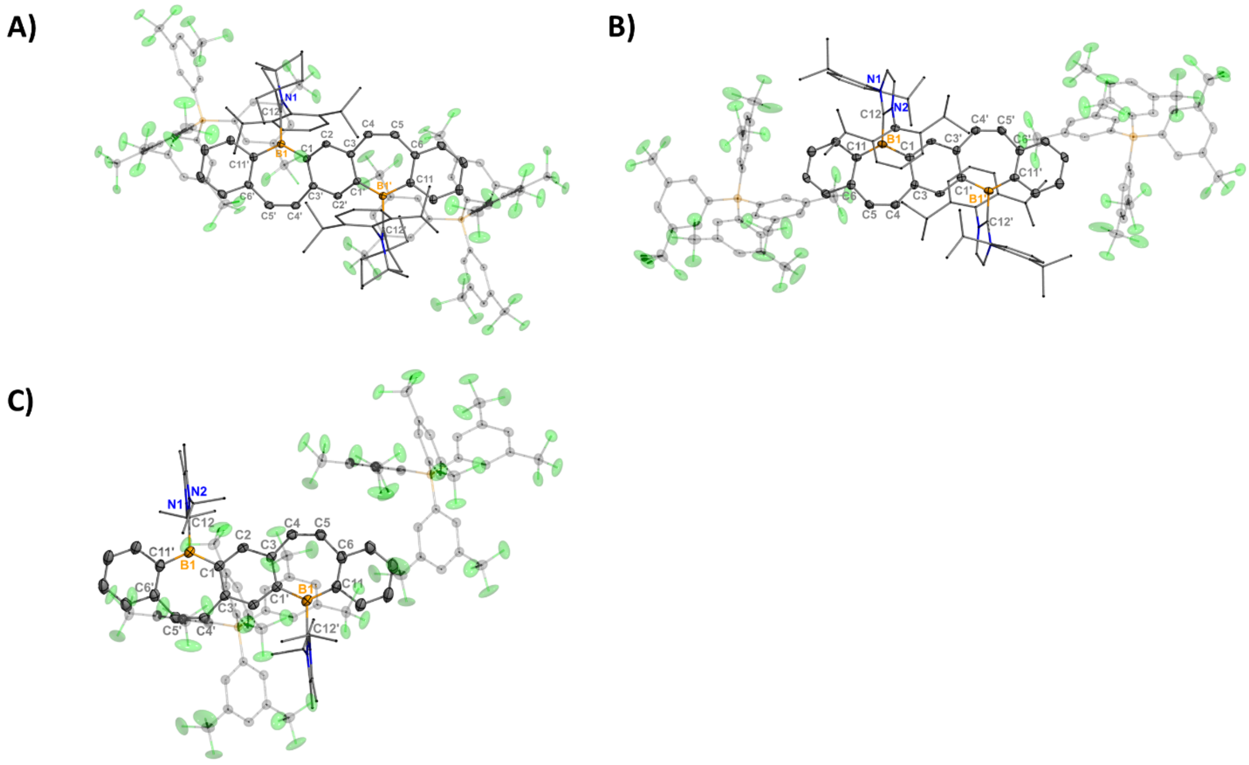

Figure S61. Molecular structures of **5** (A), **6** (B), and **7** (C) with their counteranions shown. Structures also shown in Figure 2. (Thermal ellipsoids are shown at 50% probability; H atoms and co-crystallized solvent were omitted for clarity.)

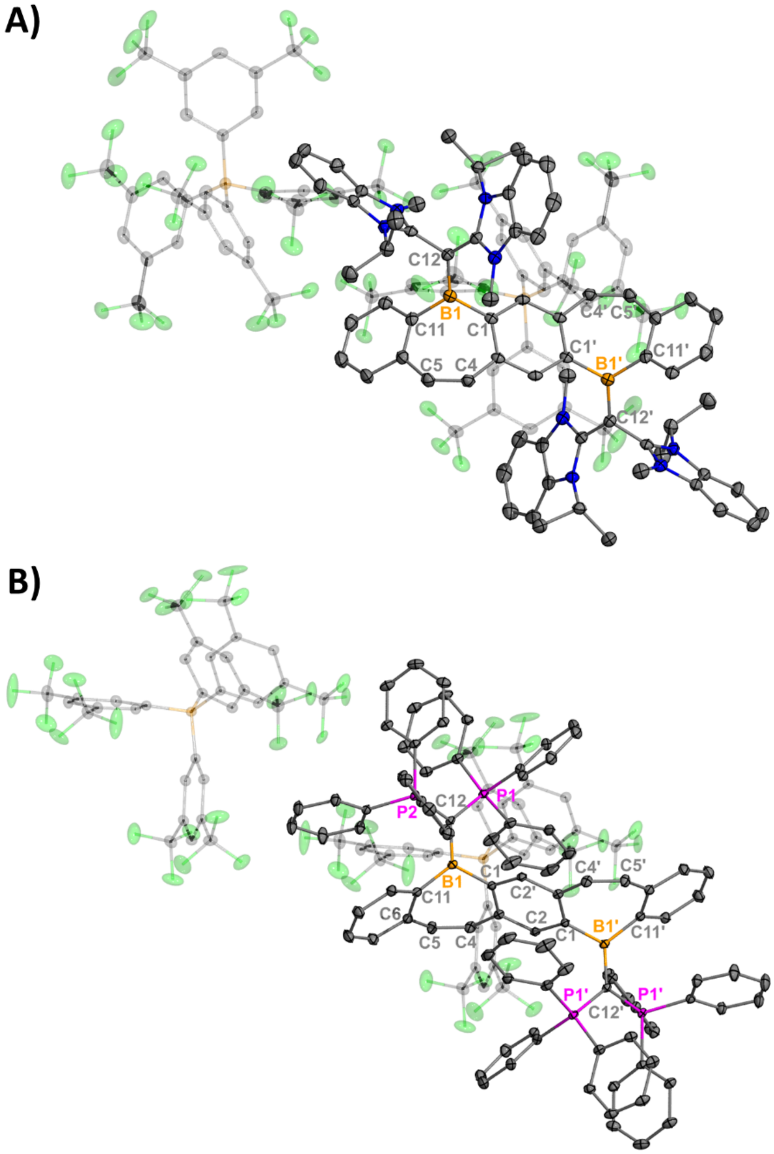

Figure S62. Molecular structures of **8** (A) and **9** (B) with their counteranions shown. Structures also shown in Figure 3. (Thermal ellipsoids are shown at 50% probability; H atoms and co-crystallized solvent were omitted for clarity.

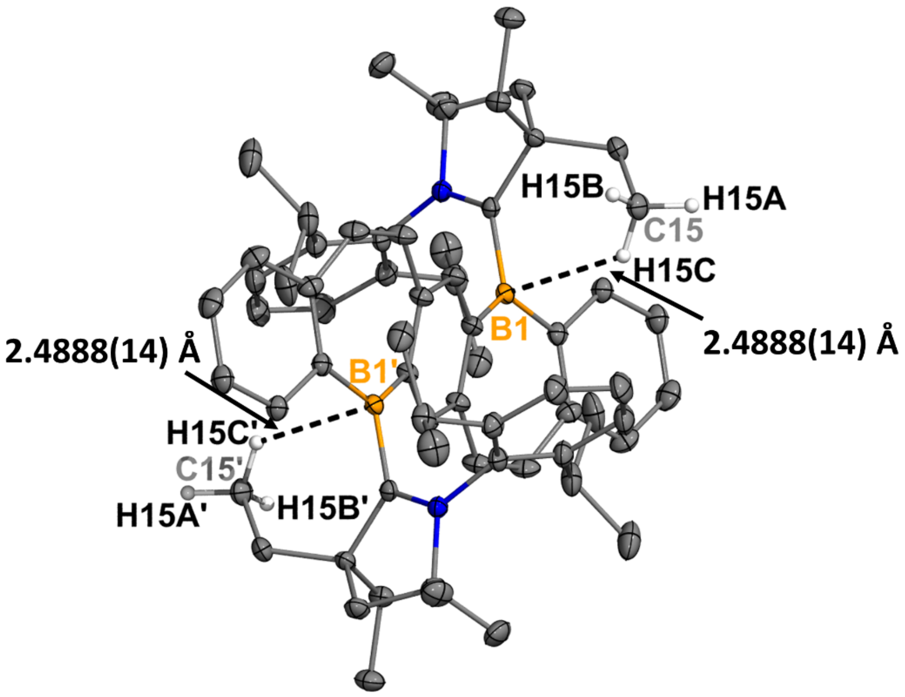

Figure S63. Molecular structure of **5** depicting close contacts between H15C–B1.

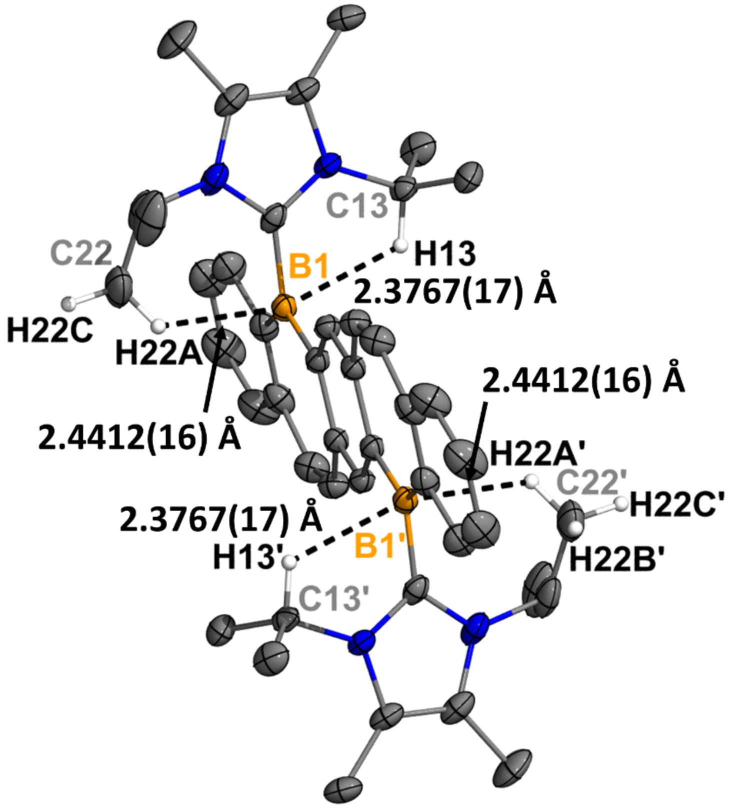

Figure S64. Molecular structure of **7** depicting close contacts between H13–B1 and H22A–B1.

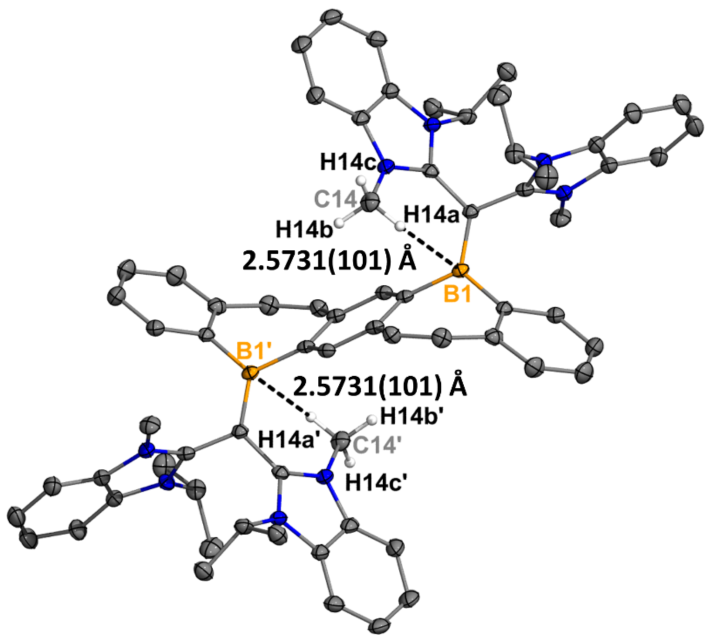

Figure S65. Molecular structure of **8** depicting close contacts between H14a–B1.

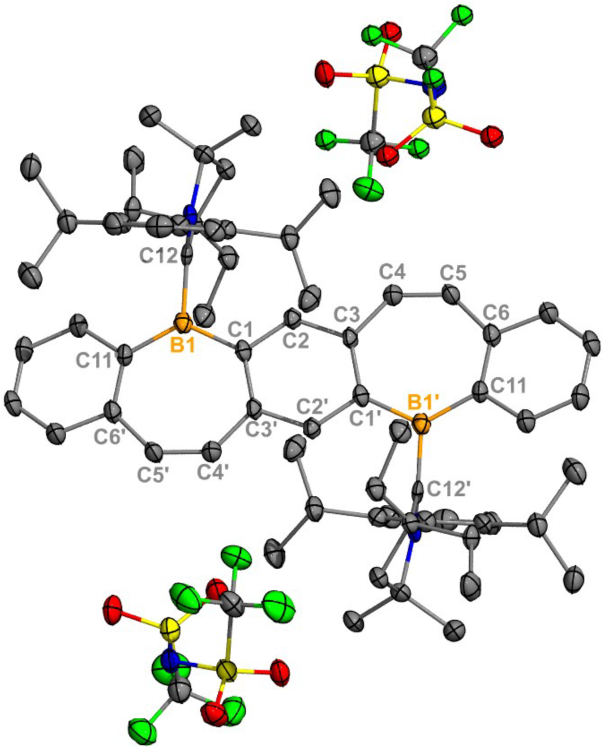

Figure S66. Molecular structure of **S5**. Isolated through the reaction of **2-Cl** with $AgNTf_2$ in Et_2O. Crystals grown from a DCM/Et_2O layer at room temperature. (Thermal ellipsoids at 50% probability; H atoms and co-crystallized DCM molecule omitted for clarity.) Selected bond lengths [Å]: B1–C1 1.555(5), B1–C12 1.611(6), B1–C11' 1.552(5), C12–N1 1.320(5), C4–C5 1.345(5), N1–C12–B1–C1 84.6(4).

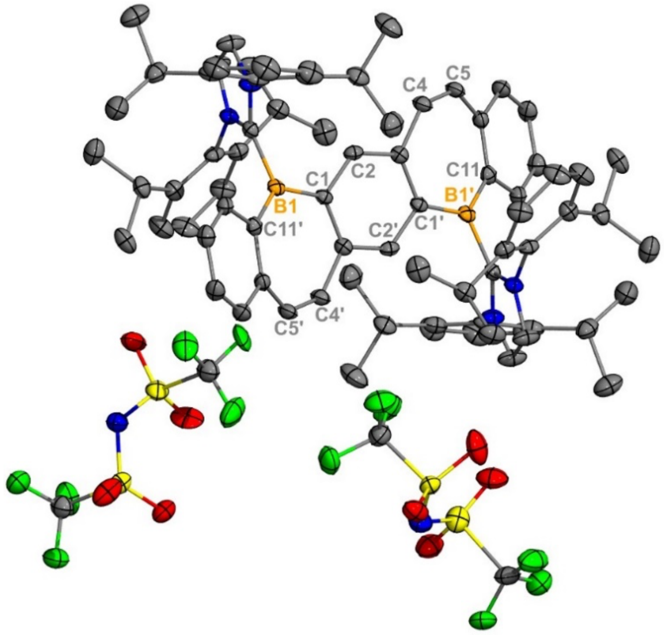

Figure S67. Molecular structure of **S6**. Isolated through the reaction of **3-Cl** with $AgNTf_2$ in Et_2O. Crystals grown from a DCM/Et_2O layer at room temperature. (Thermal ellipsoids at 50% probability; H atoms and co-crystallized DCM molecules omitted for clarity; only one of two crystallographically independent molecules shown.) Selected bond lengths [Å]: B1–C1 1.546(4), B1–C12 1.616(4), B1–C11' 1.539(4), C12–N1 1.362(4), C4–C5 1.338(4), N1–C12–B1–C1 106.1(4).

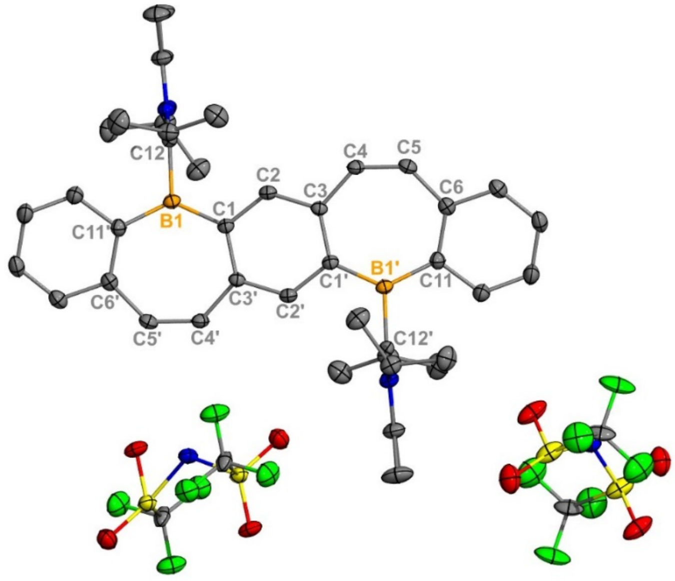

Figure S68. Molecular structure of **S7**. Isolated through the reaction of **4-Cl** with $AgNTf_2$ in Et_2O. Crystals grown from a DCM/Et_2O layer at room temperature. (Thermal ellipsoids at 50% probability; H atoms and co-crystallized DCM molecule omitted for clarity.) Selected bond lengths [Å]: B1–C1 1.545 (3), B1–C12 1.607(3), B1–C11' 1.531(4), C12–N1 1.341(3), C4–C5 1.366(4), N1–C12–B1–C1 87.3(3).

Theoretical Calculations

All calculations were performed using Orca 5.0.4, unless noted. Geometry optimizations employed the B3LYP density functional, Grimme's D3 dispersion correction with Becke-Johnson damping, and the def2-TZVP basis set, denoted as B3LYP-D3(BJ)/def2-TZVP.[16-18] All optimizations were conducted in solvent via the conductor-like polarizable continuum model (CPCM) with parameters for either Et_2O or *o*-DFB.[19] The resolution of identity approximation was applied to both Coulomb and Hartree-Fock exchange integrals with a 590-point integration grid. To ensure optimized geometries represented a minimum on the potential energy surface, analytical harmonic frequency calculations were performed. ADF 2024.1 was employed to perform the energy decomposition analysis (EDA) at the B3LYP-D3(BJ)/TZ2P level of theory.[20] NICS(1)$_{ZZ}$ values were calculated at the B3LYP-D3(BJ)/6-311+G(d)/B3LYP-D3(BJ)/def2-TZVP level of theory. AICD plots were generated using AICD 3.0.4.[21] Vertical excitations were calculated using TD-DFT at the CAM-B3LYP-D3(BJ)/def2-TZVP//B3LYP-D3(BJ)/def2-TZVP.[22]

HOMO-1 HOMO LUMO

5

6

7

8

9

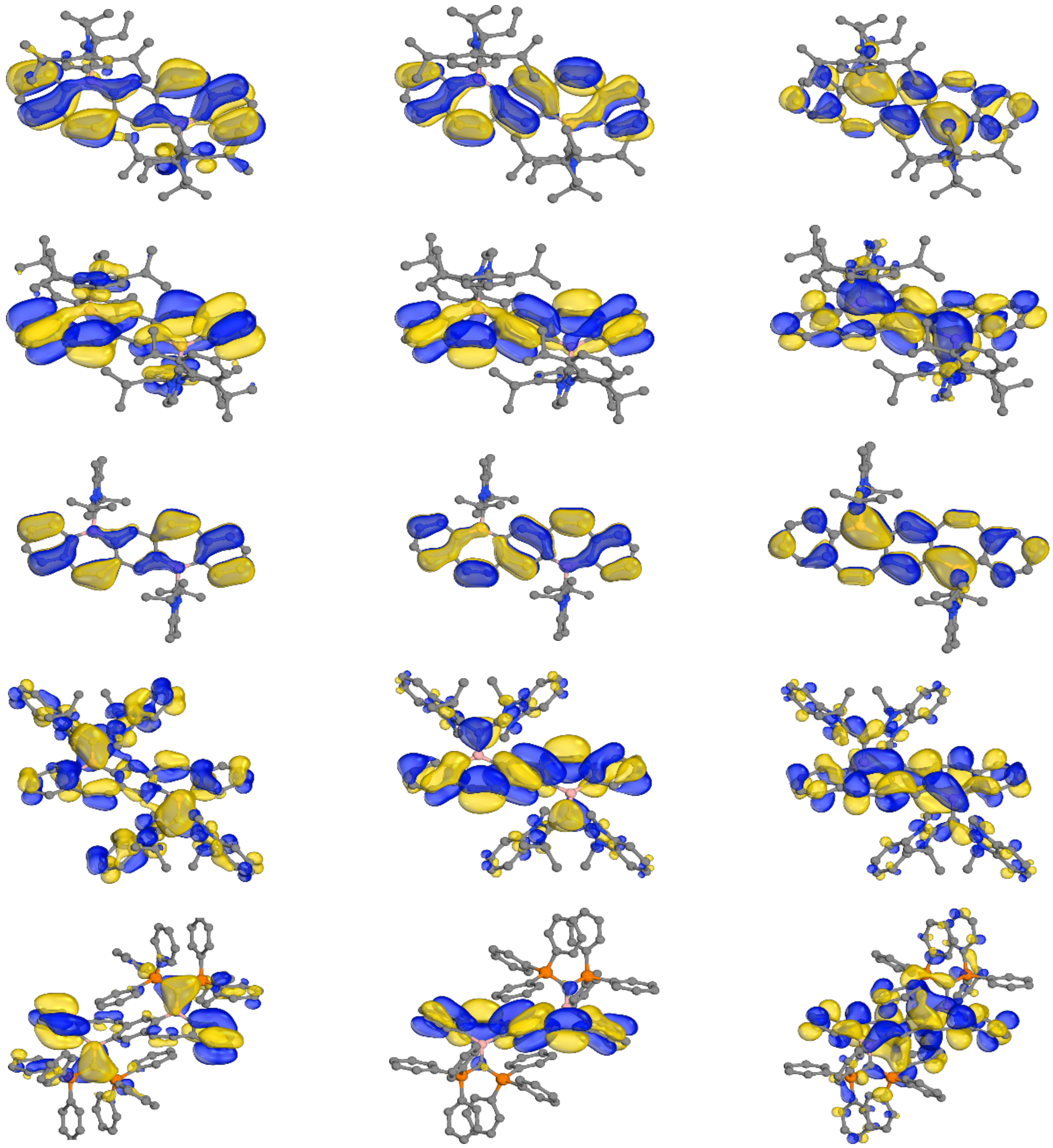

Figure S69. Frontier molecular orbitals of compound **5–9** at the B3LYP-D3(BJ)/def2-TZVP (CPCM, Et_2O)

Table S3: EDA-NOCV Results for **8** and **9** at the B3LYP-D3(BJ)/TZ2P level of theory (kcal mol^{-1})

	Compound **8**<br>2[CDC] (Singlet) + $[FBP]^{2+}$ (Singlet)	Compound **9**<br>2[CDP] (Singlet) + $[FBP]^{2+}$ (Singlet)
ΔE_{int}	-327.0	-401.1
ΔE_{Pauli}	+539.5	+630.3
ΔE_{elstat}	-347.4 (38%)	-403.4 (39%)
ΔE_{orb}	-523.4 (57%)	-548.7 (53%)
ΔE_{disp}	-40.8 (4%)	-79.4 (8%)
$\Delta E_{orb(1)\ (+,-)}$	-168.2 (32%)	-172.7 (31%)
$\Delta E_{orb(2)\ (+,+)}$	-173.2 (33%)	-175.1 (32%)
$\Delta E_{orb(3)\ (+,-)}$	-49.7 (9%)	-41.1 (7%)
$\Delta E_{orb(4)\ (+,+)}$	-43.4 (8%)	-38.8 (7%)
$\Delta E_{orb(rest)}$	-88.8 (17%)	-121.1 (22%)

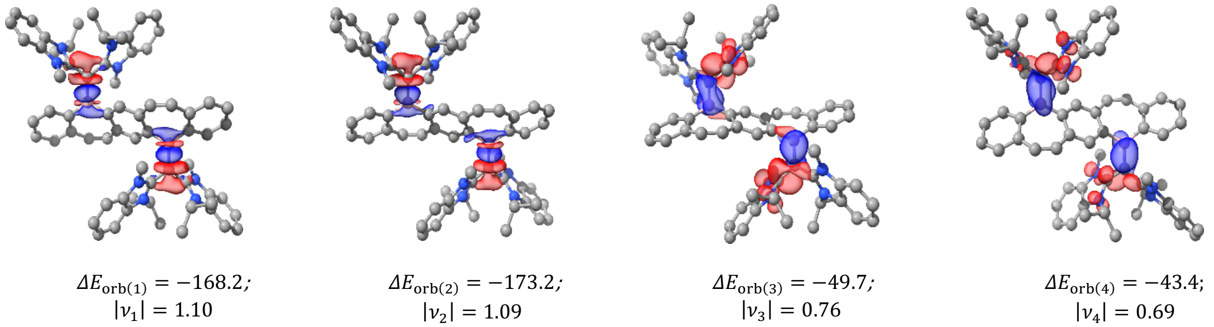

Figure S70. Deformation densities $\Delta\rho_{(1)}$–$\Delta\rho_{(4)}$ associated with the pairwise orbital interactions $\Delta E_{(1)}$–$\Delta E_{(4)}$ for compound **8**. Eigenvalues v_i quantify the amount of transferred electron density (red – charge depletion; blue – charge accumulation).

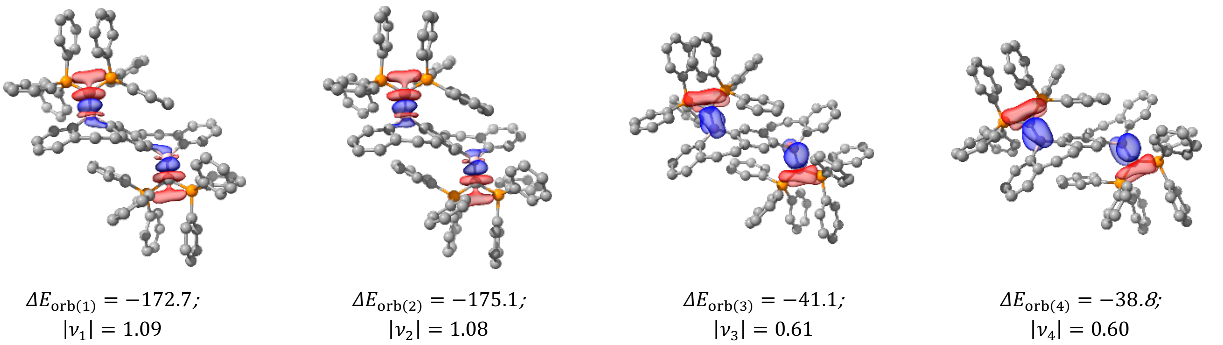

Figure S71. Deformation densities $\Delta\rho_{(1)}$–$\Delta\rho_{(4)}$ associated with the pairwise orbital interactions $\Delta E_{(1)}$–$\Delta E_{(4)}$ for compound **9**. Eigenvalues v_i quantify the amount of transferred electron density (red – charge depletion; blue – charge accumulation).

Table S4: NICS(1)$_{ZZ}$ results for carbene-stabilized mono- and diborepinium (B3LYP-D3(BJ)/6-311+G(d,p)).

	Outer annulated phenyl rings	Inner annulated phenyl rings	Borepinium
Monomeric ^{iPr}NHC-borepinium	-27.6	NA	-9.2
**5**	-26.2	-27.9	-7.4
**6**	-26.4	-28.1	-7.5
**7**	-28.0	-28.5	-9.6

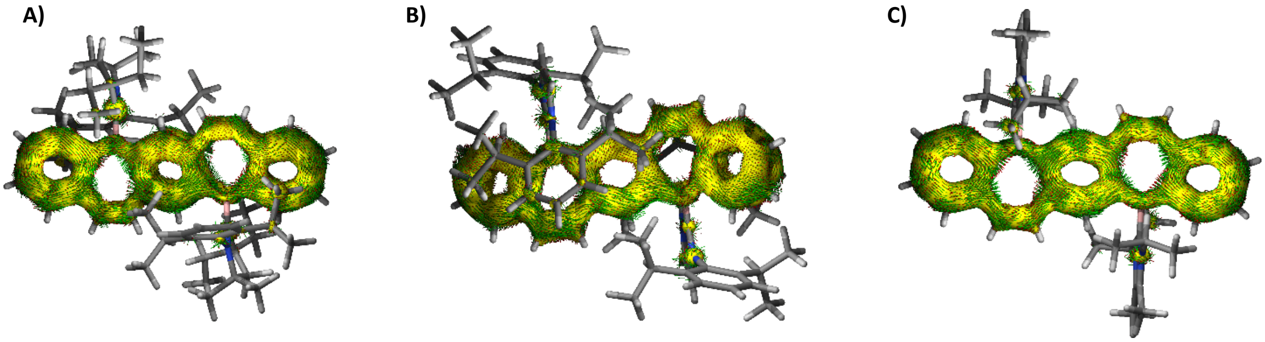

Figure S72. AICD isosurface (0.04) of the π system of **5** (A), **6** (B), **7** (C) at the B3LYP-D3(BJ)/6-311+G(d) level of theory.

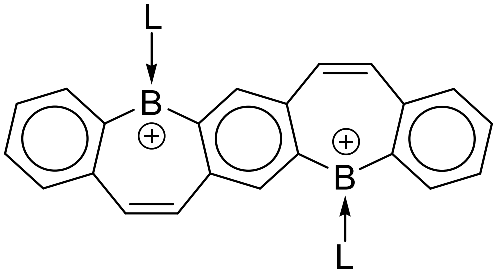

5: L = ^{Et}CAAC
6: L = IPr
7: L = I^{i}Pr

Figure S73. Clar structures of **5**, **6**, and **7**. Circles represent aromatic sextets according to Clar's rule.

Cartesian coordinates

Monomeric iPrNHC-borepinium

B	1.608539595602	2.534956142083	-0.043378323223
C	-1.542485736096	2.419667370396	-0.016506380207
C	6.277746259463	1.314514131442	-2.666402965623
C	0.711466476351	1.243827057216	-0.007410041005
C	-0.711466476351	1.222771394915	0.007410041076
C	2.770981943625	1.737376376310	-2.203497942823
C	5.040009297396	0.650841516253	-2.064565145734
C	1.381107370418	-0.001293072938	-0.000000000281
C	0.440351315901	2.363304479154	-2.956681147470
C	-1.387546116253	-0.019044826210	-0.015818595439
C	0.703360824298	-1.219448379764	0.016393801030
C	-0.696922078462	-1.226812173219	-0.000575205381
C	5.148709456883	-0.870815360066	-1.941861780525
C	3.402669781887	0.536191650074	-4.018239765147
C	2.052913023565	0.903783104109	-4.175447890799
C	4.066529303595	-0.186876249265	-5.014592176291
C	1.315738231421	0.551295400658	-5.307546236003
C	3.332763026939	-0.537679263529	-6.151410800990
C	1.980368825449	-0.178039060072	-6.296416284765
C	2.815756863982	2.492593592945	-0.984393529823
C	-1.259025813537	3.704939546900	0.309151550024
C	4.205880970413	4.584061040305	-4.141912484861
C	1.265334600780	3.805191297426	0.824965466658
C	-0.061120062975	4.337216866269	0.860776407217
C	3.991185270973	3.326314905529	-0.835911362237
C	2.265284415768	4.546736666428	1.492314195720
C	3.540404095942	4.828476437651	-2.789214658549
C	-0.287479175667	5.593176782996	1.476557322288
C	4.951910947325	2.253659172614	1.227229668424
C	2.016722482953	5.757613670370	2.136119227196
C	0.726906048665	6.298955977976	2.111059357318
C	3.124476453209	6.278047629744	-2.538427543341
C	5.577836281031	4.880723345396	-1.227605821996
C	5.914079100202	4.189024672836	-0.046691301441
C	6.405218862538	5.896645887280	-1.720830471041
C	7.067427065778	4.480957176553	0.685302297001
C	7.558721908693	6.189816114529	-0.991750727173
C	7.886390859090	5.496099287835	0.190532688605
H	4.437558628128	3.517212172988	-4.277478030175
H	7.160528559957	1.067986203110	-2.057734890810
H	0.532186258243	3.172376040861	-2.222566608193
H	4.894882147290	1.035327229584	-1.052017298551
H	-2.583900022001	2.230090865002	-0.298218515774
H	2.473383855381	-0.007703099800	-0.006474636464
H	0.194041884017	2.789339795041	-3.939367831966
H	6.466511753135	0.969002129579	-3.693687817866
H	-2.480822451507	-0.021842159516	-0.035904408577
H	5.967108211526	-1.113492117931	-1.247444539276
H	-0.363597884369	1.689587561515	-2.633107388785
H	1.261243128941	-2.159457090410	0.028469326177
H	-1.246005606128	-2.171941773874	-0.004059603445
H	4.215688239424	-1.292602114733	-1.538498542573
H	5.367472462476	-1.358137445540	-2.901829318874
H	5.114716172174	-0.467188595741	-4.924385967845
H	0.267547253307	0.832882793312	-5.415808364651
H	3.824486565496	-1.104203379800	-6.945261603892
H	1.439793254295	-0.471594527770	-7.198937651322
H	6.168078233617	2.408235566648	-2.688035140557
H	3.518397771394	4.891010325146	-4.944131410392
H	-2.110206621471	4.390554403728	0.241637635264
H	2.633495821587	4.216649391797	-2.734507748752
H	4.095520571848	1.580538469938	1.112410012270
H	3.283051363324	4.169872736798	1.524401556679
H	5.136119094979	5.160177368292	-4.255434219452
H	-1.301015891752	6.002911444539	1.465632796541
H	5.889036053532	1.680803981980	1.171346132787
H	2.381530532532	6.572310329044	-3.294768603591
H	4.893520410705	2.754478924606	2.204519467916
H	2.830786006558	6.283477718749	2.641580013695
H	0.514873402138	7.258718046802	2.588801833670
H	2.664316289417	6.377930515155	-1.543597100847
H	3.969902037482	6.977442452882	-2.606694084060
H	6.171550748020	6.441462511595	-2.633939395088
H	7.315928141171	3.939258568281	1.598898554220
H	8.224724056739	6.977750970098	-1.350053905817
H	8.799848643331	5.757562543071	0.728993466857
N	3.808258259872	1.042384993960	-2.780320770396
N	1.702501511829	1.652966970908	-3.056812020583
N	4.377466058617	4.326153692180	-1.677798754252
N	4.920977815784	3.238184900694	0.157090437645

5

B	1.588959134780	-2.529368839775	0.108514912664
B	0.073310737651	-7.997544535867	2.040444841650
C	0.728517155954	-0.121559522764	-3.191490616404
C	0.713372276817	-1.256943730431	0.023088260073
C	1.129782690868	-3.954890411360	0.504705095369
C	0.709725129627	1.217802547359	0.006431502381
C	2.199239295708	-11.839347873555	1.957379268891
C	1.881765128641	-6.177032558905	1.373896508141
C	2.328033480751	-9.389521378763	1.901860919290
C	1.372989489520	0.000000000000	-0.000000000013
C	2.111310426734	-4.868329184417	0.912417299031
C	2.935923657013	-10.667947419901	1.857371090017
C	2.605360728683	-6.153947570718	-1.904220727221
C	2.347243481682	-2.144068350502	-4.782301702695
C	2.575804296637	-3.454392927678	-5.183405668955
C	2.261840567667	-0.264041475442	-3.163702134771
C	3.072383974934	-6.945914181574	1.651214975625
C	3.255575203857	-8.278776002600	1.833485987706
C	2.642012921024	-1.704531976166	-3.483877951993
C	3.058252302976	-4.371814117364	-4.259012225057
C	2.586934349363	-1.616951076274	3.090395618254
C	3.360206921507	-4.006402483140	-2.938846933539
C	3.184974154442	-2.644608012641	-2.566273343497
C	2.877534404092	0.764763571357	-4.128157343987
C	3.167100411972	-2.243948378358	-0.102163149230
C	3.767672049230	-5.145621690199	-2.010190368334
C	3.623224750291	-0.988855565655	2.160060023452
C	4.179305863659	-1.861885466032	1.006573938755
C	5.033967995899	-5.891294970839	-2.463680281104
C	4.802030084789	-3.163456348967	1.623609508646
C	5.178342630290	-1.648996578090	-1.242678954908
C	5.211537895137	-1.073313444226	0.176308686142
C	5.398361992650	-0.562679854159	-2.287715576653
C	6.180493211918	-2.775488769423	-1.495304359347
C	6.074914350121	-2.946559532481	2.442009808851
C	1.478343077479	-10.340948379878	5.263205054020
C	0.906664432371	-9.297893987592	2.077561192942
C	0.529030770788	-6.628565258290	1.474631168503
C	0.814409989269	-11.766532526149	2.158876475444
C	-0.691048161291	1.231923656643	-0.006432517808
C	-0.218627808885	-4.430812872339	0.548598778759
C	-0.722066749261	-1.222891541493	0.004508853225
C	0.206604723662	-10.522106792684	2.235116502922
C	-0.451649331759	-5.733628798518	1.028286605964
C	-1.382971985412	0.030109067921	-0.027596097858
C	-0.268115793887	-4.164378391897	3.818039259290
C	0.483155977264	-8.002446611934	6.904953473532
C	0.475313990171	-6.644486195065	7.198319695639
C	-0.001764438700	-10.019800422944	5.542732150342
C	-1.406326046915	-3.690045219433	0.196202999024

C	-1.611329669496	-2.365166186073	-0.018855423229
C	-0.167732505723	-8.521123460630	5.776658263693
C	-0.153817782891	-5.765322750924	6.326522557820
C	-1.549388818756	-9.051569215021	-0.548741672624
C	-0.817381926356	-6.209753546607	5.173063440012
C	-0.850660015280	-7.611081101056	4.922702049468
C	-0.472245999378	-10.878329701865	6.731157652864
C	-1.443549424395	-8.173592441753	2.576273623797
C	-1.391825717857	-5.115539784633	4.276220570637
C	-2.480053086033	-9.515510565006	0.570520893682
C	-2.704703271438	-8.555183443248	1.765481519684
C	-2.487315298968	-4.279849826170	4.962534651769
C	-3.410277377566	-7.255302182298	1.228823441921
C	-3.162608479799	-8.555023453279	4.187370510931
C	-3.537419052085	-9.236841759738	2.869001154832
C	-3.206567420103	-9.536770348158	5.350010750938
C	-4.019261386325	-7.342022131058	4.551149744282
C	-4.902481970947	-7.405674750815	0.927685046269
H	0.348204442140	-0.257347509972	-4.215847495345
H	1.890489479980	-9.717092649515	4.461667474246
H	0.432475282796	0.881295563750	-2.850459204764
H	1.660319963742	-5.664210006447	-1.634292083457
H	1.276651937283	2.151126482744	-0.000358805023
H	2.700790880307	-12.808418591571	1.901959696853
H	1.928091949327	-1.432584501243	-5.495056921046
H	2.358431846266	-3.764808013341	-6.208068875214
H	1.684472001840	-1.951879139085	2.556759736695
H	2.456036901379	-6.668327888140	-2.865742782918
H	2.828975162775	-6.919741010922	-1.147763884820
H	3.199800630904	-5.409995629062	-4.563101504109
H	2.439138192755	0.679208840003	-5.134357875077
H	2.458247805464	0.033281225518	-0.031040153337
H	2.259939689934	-0.874058612273	3.833690697245
H	3.148957321656	-4.572839086960	0.849419251490
H	2.606223289987	-0.026831474831	-2.151235017410
H	2.983716041482	-2.481150697114	3.643330268284
H	2.664945625266	1.782760116146	-3.765008896792
H	4.020361742228	-10.723359199225	1.738508421062
H	3.992996565861	-6.355749769674	1.620594923699
H	4.302038406122	-8.586778814077	1.917610786851
H	3.958786867575	-4.736495860047	-1.011771556052
H	3.225937737133	-0.052625481512	1.748360426066
H	3.965561966041	0.657350830047	-4.227265484254
H	4.865968208257	-6.407058451633	-3.421845781773
H	4.050896904302	-3.627926215658	2.272283517960
H	5.297952139849	-6.658695225794	-1.718626600740
H	4.498218964836	-0.684589552951	2.755485191882
H	5.019268875317	-3.895585172293	0.833880189740
H	4.723476768338	0.287944266330	-2.141566407802
H	4.905811102104	-0.016654335322	0.140913687917
H	5.293579268950	-0.949372862726	-3.310184012380
H	5.897437611342	-5.225847817586	-2.587834445638
H	6.081936105433	-3.153047639228	-2.521467543978
H	6.081041159602	-3.613858567298	-0.797807008867
H	6.219383834159	-1.106834183796	0.604110605066
H	6.429488152710	-0.197398279462	-2.170076881688
H	5.935463801864	-2.207456247202	3.245083964584
H	6.362531962732	-3.897892923906	2.915726033651
H	6.922852876994	-2.621436211168	1.821495462080
H	7.192991854577	-2.361281934882	-1.386730653031
H	2.086218814781	-10.176447595956	6.166223328808
H	0.237343187972	-0.862516914382	-2.548097686503
H	1.589171181370	-11.395488885582	4.970700986205
H	0.573747102033	-4.702702338002	3.365887695892
H	0.220296464297	-12.675648373391	2.273474287206
H	-1.236268731057	2.178718963289	-0.015713332952
H	1.010710801851	-8.688317529045	7.569349007448
H	0.974640186063	-6.270125142562	8.095050575692
H	-0.513371992986	-8.913879400384	-0.204425221854
H	0.122102881686	-3.583728826753	4.667529989121
H	-0.658723947607	-3.447984327765	3.081247746344
H	-0.130108455666	-4.695853291165	6.539941668182
H	0.185546567583	-10.737293221148	7.602412017205
H	-0.863375405454	-10.504941133834	2.425677377104
H	-1.520539842706	-9.810966557374	-1.344746139291
H	-1.488141411884	-6.037626292478	1.075314935014
H	-0.582436191527	-10.305961707145	4.657520258357
H	-1.876204074423	-8.107512336140	-1.009305427552
H	-0.428086098240	-11.944159174351	6.456914598114
H	-2.474694425686	0.040283651379	-0.060634257542
H	-2.311488277522	-4.303657915562	0.162831841748
H	-2.655229161849	-2.092548619633	-0.199900662663
H	-1.826350735921	-5.578546973124	3.381826706395
H	-2.141490306385	-10.491060017926	0.945497089495
H	-1.497411980064	-10.648537371073	7.047082706444
H	-2.071996763019	-3.711883159399	5.809544935379
H	-2.909442367091	-6.955469147433	0.300811715336
H	-2.900023514148	-3.552187883370	4.245927134123
H	-3.479363613174	-9.707062883645	0.148272501489
H	-3.279002643384	-6.420496834347	1.929978752580
H	-2.640639092358	-10.449864534614	5.137835665660
H	-3.250764394927	-10.297913911427	2.924154924761
H	-2.847215399904	-9.082733155076	6.282864000968
H	-3.314803430565	-4.890337400762	5.342852885662
H	-3.672668138703	-6.905192422171	5.496706394687
H	-4.029001153821	-6.562554246596	3.782361273442
H	-4.615515379232	-9.201784261405	2.680837659625
H	-4.258771139014	-9.821681101844	5.498826446161
H	-5.112965755270	-8.284533825320	0.299498934762
H	-5.249335095540	-6.516761675257	0.378567713384
H	-5.510325968554	-7.483717707462	1.840523349129
H	-5.052452843659	-7.686511892324	4.700451926895
N	3.731940397022	-2.189228485334	-1.279686741788
N	-1.720292667248	-8.117543517963	3.853022138950

6

B	0.707583968541	0.474712956502	-2.860742037043
B	-0.709468797049	-0.476730918657	2.862893215332
C	0.202053273562	-1.315740088057	0.519690069393
C	0.015482830816	3.417648374544	-1.935488158550
C	0.275871203938	0.284632389535	-1.384771131502
C	0.453204119779	-2.689422412700	0.897510366378
C	0.455196250059	-0.987532174700	-0.822910569806
C	0.464790455918	-0.848186684176	-3.760135683994
C	0.434922151060	-2.802180380836	-4.871233114262
C	0.853377434995	2.481161233283	4.578424443453
C	0.947580964653	3.087583818912	-2.998490209442
C	1.292226631989	1.769280900078	-3.443995198902
C	1.248717715118	-2.510990401158	5.914455910943
C	1.364933192653	-1.020872396218	5.580682401145
C	1.520061012739	4.201359051946	-3.658111051721
C	1.834480120531	-1.083065056055	-7.141193813484
C	1.802947417612	-2.270580959768	-8.119598907390
C	2.223161177820	1.663716840197	-4.504776272620
C	1.952562916958	0.452038221268	3.547952759354
C	1.985415092540	-0.243943741440	6.757514439000
C	1.986083882515	0.241623226130	-7.903067298273
C	2.148088330178	-0.730352076226	4.303115325401
C	2.451981841711	4.054057825423	-4.676849690409
C	2.635274065797	-1.675511743188	-4.751314837941
C	2.819199567251	2.768719842052	-5.097946550136
C	2.642736889625	2.165920485793	1.757412367544
C	2.792158778137	0.837167831462	2.483642971060
C	2.917025749213	-1.300867146513	-6.087453591499
C	2.954249267730	2.068192228594	0.257811725852
C	3.330180941273	-2.454991011331	-2.406547089175
C	3.188159062130	-1.577850126590	3.897944849102
C	3.638313789606	-1.962309480414	-3.809169978930
C	3.743835367388	-1.422507491432	-1.348099597528

C	3.544320018366	3.236309604702	2.402871179921
C	3.966080716609	-3.826581158526	-2.133365585534
C	3.825417696331	-0.044070876141	2.137152280949
C	4.009465947302	-1.244525433499	2.821312231070
C	4.265185501001	-1.165153227445	-6.440261565796
C	4.971065710752	-1.796835347529	-4.215271614829
C	5.282001677089	-1.393972145155	-5.511837982308
C	-0.209793934102	1.311645109658	-0.519393554761
C	-0.023431167224	-3.421288059945	1.936873324022
C	-0.282037594790	-0.288320549175	1.385449413243
C	-0.463442849605	2.684789506817	-0.897989715537
C	-0.462159132933	0.983608203737	0.823193052217
C	-0.466165369093	0.848877298345	3.758354920324
C	-0.436938729927	2.806865520157	4.862280645510
C	-0.855571603511	-2.474840188565	-4.590181406796
C	-0.948441177933	-3.089536181195	3.005428305371
C	-1.289842303382	-1.770549806292	3.451632068874
C	-1.243550446758	2.521380790775	-5.911692682484
C	-1.359836691984	1.030199106607	-5.582655844113
C	-1.516253488319	-4.202372139533	3.670794137449
C	-1.825091698950	1.102413271825	7.146455366594
C	-1.804840131314	2.291994125350	8.122624965017
C	-2.212104953656	-1.663601291155	4.519909331944
C	-1.954359765151	-0.448185569933	-3.555878417511
C	-1.974248272959	0.255953492738	-6.764403477701
C	-1.962115528820	-0.222842651156	7.910233851964
C	-2.147974680417	0.735752063243	-4.309017195616
C	-2.439649189208	-4.053622282811	4.697013727048
C	-2.635103751827	1.673900777013	4.753997071072
C	-2.803004625920	-2.767686087822	5.119759113048
C	-2.647762399061	-2.165894738164	-1.770523573458
C	-2.796563435057	-0.835938785863	-2.494659955106
C	-2.911004846597	1.307052014757	6.093515592833
C	-2.959023644419	-2.069892773814	-0.270774146443
C	-3.341592795263	2.434175282155	2.406333860096
C	-3.190054920960	1.581533721576	-3.905404978978
C	-3.642628838111	1.948787794408	3.813025777655
C	-3.755827453040	1.394394269015	1.355341212288
C	-3.549493611060	-3.235058398213	-2.417693530966
C	-3.982764112864	3.802224468577	2.127780790907
C	-3.831612834548	0.043727513076	-2.149547772900
C	-4.014575853354	1.245272138928	-2.832108958170
C	-4.257416463014	1.166220324192	6.451134815505
C	-4.973228485845	1.778386750440	4.224024542250
C	-5.278212288791	1.382497771393	5.524155936525
H	0.273745210935	-4.474495458075	1.931226014619
H	0.813293424591	-1.795708900463	-1.457306164127
H	0.339087375826	-0.651415527081	5.457945594001
H	0.862293197720	-3.652373838474	-5.393759186374
H	0.552225070961	-2.644376491150	6.756146077302
H	1.067614365246	-3.242312821506	0.179767297044
H	0.858713224999	-1.045199946571	-6.638336906859
H	0.864826164363	-3.088943294521	5.061912634337
H	0.980441681097	-2.149406872144	-8.842229368653
H	1.226260301374	5.204118350351	-3.339314617664
H	1.153353001340	0.354507757346	-8.614807580251
H	1.392637948003	-0.404384708609	7.671797024875
H	1.785590539922	2.979303531015	4.822784218539
H	1.662450440665	-3.228688422880	-7.596435017818
H	1.596506132505	2.491647119588	1.862259228040
H	1.967675060912	1.105088848019	-7.223609449409
H	2.019803958118	0.838248429691	6.561738316502
H	2.243803418966	-2.600117729685	-2.341324086533
H	2.504726249896	0.677648154674	-4.861443752370
H	2.216224490444	-2.940996238614	6.217662525759
H	2.440365583531	1.219578410753	-0.207357227103
H	2.889631193217	4.937075658864	-5.148724153140
H	2.746542407936	-2.333665039138	-8.685043014646
H	2.629201592767	2.985423807311	-0.254737560854
H	2.922195106768	0.278139843812	-8.481777424297
H	3.014780031990	-0.587503211647	6.947805004330
H	3.550374399013	2.632212842810	-5.897560155993
H	3.307890586463	-0.434145008094	-1.554867686192
H	3.413426678440	-1.734808831836	-0.346065835833
H	3.648643432039	-4.569307082313	-2.881789525981
H	3.369752246967	-2.504342895449	4.442887215137
H	3.663668805868	-4.189379196743	-1.137764450862
H	3.342048255181	3.368715606001	3.475266462712
H	3.399309113973	4.208023659952	1.904740663705
H	4.034141465353	1.951937981044	0.077432005021
H	4.492249784283	0.211394949520	1.313295274679
H	4.523284587935	-0.873048793005	-7.459814646515
H	4.838946760724	-1.304038095578	-1.323002187884
H	4.604220501057	2.953680201526	2.299342565440
H	5.066038766282	-3.773015060923	-2.150547582010
H	4.812974267913	-1.922014468899	2.522420780623
H	5.774660571085	-1.998332326488	-3.504076973865
H	6.326380399398	-1.271448501870	-5.808723708911
H	-0.284149831505	4.470163048127	-1.931603445177
H	-0.820230534569	1.791856262059	1.457497049691
H	-0.334107353078	0.661430228139	-5.456496015766
H	-0.864425745159	3.657840454495	5.383492234112
H	-0.543014787736	2.657987010577	-6.749511471302
H	-1.082071924971	3.235968591641	-0.182544171683
H	-0.849334848773	1.074236087348	6.643090443520
H	-0.864433600343	3.097169246724	-5.055568670921
H	-0.980227044880	2.180746472070	8.844456269902
H	-1.225365884917	-5.205595070508	3.350801513773
H	-1.129433149838	-0.324507764464	8.623711666321
H	-1.377306384963	0.419285591810	-7.675475261398
H	-1.788179442777	-2.970166307098	-4.838794703461
H	-1.674701760613	3.250369290760	7.597147478292
H	-1.601652514645	-2.491817271696	-1.875833947512
H	-1.931937396106	-1.087064138704	7.232120232208
H	-2.008756580624	-0.826789494755	-6.571771405213
H	-2.256022016948	2.582225981151	2.336010321446
H	-2.490358835881	-0.677061689412	4.877831337651
H	-2.210035365296	2.951262883907	-6.218297700889
H	-2.444266306843	-1.222264614754	0.195180497862
H	-2.873310485503	-4.935993117545	5.173757912021
H	-2.748221975974	2.346766110378	8.689272004437
H	-2.634544441360	-2.987978331636	0.240639098859
H	-2.898912415953	-0.269828648848	8.487103618662
H	-3.002961270019	0.599280250583	-6.958576654327
H	-3.527295630091	-2.630042318266	5.925420481647
H	-3.317035213375	0.408313856555	1.566927653077
H	-3.428992184432	1.701358360095	0.350483253750
H	-3.664275571439	4.550265629132	2.870436029569
H	-3.370673283123	2.509037325794	-4.448950715746
H	-3.685679333228	4.160035260560	1.128767794743
H	-3.346563718264	-3.366148654349	-3.490144957557
H	-3.405080292444	-4.207465090556	-1.920736527830
H	-4.038771208676	-1.952852680812	-0.090023645230
H	-4.500634042449	-0.213871342322	-1.328124212878
H	-4.510833912249	0.879763696339	7.473483900868
H	-4.850716937589	1.273094902154	1.334107124451
H	-4.609357016200	-2.952211080606	-2.314354843833
H	-5.082474271820	3.745543815562	2.150013502839
H	-4.819714802855	1.921439256877	-2.534615979042
H	-5.779920212095	1.970461276901	3.513723169079
H	-6.321007249908	1.256044665157	5.824928790660
N	0.816176470517	1.277586140087	3.897215747920
N	1.236399320419	-1.779197286531	-4.381620284438
N	-0.817930853487	-1.273804882599	-3.904578914929
N	-1.237860818476	1.780477536140	4.378852147211

7

B	-0.412876402938	8.329570389215	0.519115121236
B	-1.636241203837	2.485510714247	0.018815234177

C	0.329883938174	8.797468057939	3.436809533970
C	0.245995905392	5.904343456782	0.249078511135
C	1.708700641771	8.307505008951	3.012040449044
C	-2.131040776683	6.306020839429	0.399450132634
C	-3.953553406504	3.998543224286	-2.738240181582
C	-0.799324886670	6.834080724226	0.387624684951
C	-3.352759561894	7.070096825685	0.503612148455
C	4.477524722931	9.047086058508	2.061684595313
C	3.306504273582	8.928533480496	1.147905609170
C	1.166805603067	8.615182284967	0.550283840204
C	-3.602234202446	8.407042369530	0.548479732381
C	-1.313874267766	9.577993922378	0.530115675696
C	-2.747864934166	9.576215438366	0.531803130090
C	3.228521785597	9.095062267176	-0.214587159257
C	-0.668990047703	10.844921610888	0.547902447768
C	1.738260583016	7.735154333992	-2.755736321418
C	1.271652949775	8.916977853406	-1.906950450950
C	-3.436087997708	10.815257762200	0.537713817340
C	4.312946407632	9.460910820082	-1.171448908258
C	-1.362063278785	12.047142313367	0.547105039288
C	-2.765060337222	12.029502927177	0.539133076958
C	1.431004199241	10.273170373597	-2.589185181299
C	-2.379039813484	2.013573138893	-2.898212703734
C	-2.295138067733	4.910906994552	0.287046843992
C	-3.757837768042	2.504135804940	-2.474083969462
C	0.081891376666	4.509231746818	0.136589646536
C	1.904467196022	6.812757200026	3.274284633367
C	-1.249809841346	3.981133826952	0.148833348546
C	1.303590911342	3.745174854356	0.031996251571
C	-6.526688499747	1.765878659502	-1.522653787380
C	-5.355632657965	1.885644084067	-0.609072355580
C	-3.215915941700	2.199835581620	-0.011950419930
C	1.553098523131	2.408222258143	-0.012529574759
C	-0.735181543029	1.237137802087	0.008352296688
C	0.698807964066	1.239006261679	0.005519998668
C	-5.277614572762	1.721092661547	0.753658080724
C	-1.380009439470	-0.029840289980	-0.007684974077
C	-3.787183302762	3.084955340261	3.292556147881
C	-3.320722664660	1.901683399873	2.445700376831
C	1.387100807988	0.000000000009	0.000000000000
C	-6.362014386448	1.356738803291	1.711116275156
C	-0.686860770489	-1.232017088276	-0.006412762158
C	0.716142980933	-1.214286685520	0.000225440877
C	-3.480296665964	0.546627120644	3.130140329234
H	-0.478104714138	8.215874120311	2.965920631599
H	2.455034903288	8.868772103198	3.589680601893
H	1.273534632977	6.268685819869	0.233988803651
H	-2.280146438366	2.149273107637	-3.984947439883
H	4.638368389366	8.126084600136	2.643321402267
H	-4.935285874052	4.346924503598	-2.384998608394
H	0.183097242736	9.860675936707	3.203471719101
H	-3.172711713891	4.591679267064	-2.240142661401
H	-4.249746784821	6.443537570229	0.527527787769
H	-3.889257426952	4.187801289634	-3.820074046038
H	4.350295749264	9.877467834391	2.775463999026
H	5.387187645905	9.242799708788	1.479980150920
H	-4.665532565988	8.660110199075	0.600331060603
H	1.595736078529	6.784879378166	-2.220800540955
H	0.420949228081	10.887162860253	0.566225925273
H	0.198433391787	8.777130785872	-1.708014230031
H	5.286260879671	9.378971827656	-0.670874558351
H	2.796220336831	7.822695554949	-3.041161747740
H	-4.528404661379	10.805176451421	0.542004694513
H	4.330656591806	8.801266090427	-2.049759232349
H	1.143347947345	7.703247222042	-3.680758594716
H	4.207165857364	10.496953475622	-1.529181924699
H	-0.819905225394	12.995332086965	0.557365724150
H	-3.329042581328	12.965435835468	0.540230319669
H	1.110611668232	11.088240887866	-1.923929562294
H	2.465936153419	10.459674411671	-2.907230052466
H	0.796713115175	10.293078058172	-3.487859937529
H	-1.571026902889	2.595667096997	-2.427969759173
H	-4.504196725750	1.942164806845	-3.051016726589
H	-3.322675303593	4.546570987549	0.302173216369
H	0.231062366973	8.660478988028	4.523389551925
H	-6.687398003235	2.686030095220	-2.105670261575
H	2.886241944376	6.464868794127	2.920673088481
H	-2.232337699131	0.950636692519	-2.663606500775
H	1.123698868985	6.220233623909	2.775338966172
H	2.200534627389	4.371767058466	0.007309791707
H	1.840093155838	6.622092141401	4.355866813062
H	-6.399573382172	0.934408891918	-2.235184194789
H	-7.436370783070	1.571164607041	-0.940644172309
H	2.616372816029	2.155184769693	-0.065017229959
H	-3.644512473748	4.034325259704	2.756065069935
H	-2.469966864452	-0.072163880582	-0.024951861919
H	-2.247485358898	2.041022350414	2.246525387386
H	-7.335368839811	1.438188191807	1.210538209667
H	-4.845177109457	2.998008016917	3.578048708447
H	2.479412727211	0.010139790961	-0.005253400235
H	-6.379490171707	2.017577969310	2.588536047349
H	-3.192315629942	3.118282948121	4.217555084236
H	-6.256384904664	0.321163041058	2.070246997636
H	-1.228967015689	-2.180250676713	-0.015302178738
H	1.280179261802	-2.150187419650	-0.000589792051
H	-3.160152541023	-0.269629160595	2.466198991131
H	-4.515237102125	0.360862921915	3.448594312080
H	-2.845925259480	0.528058426051	4.028789161458
N	2.017216327849	8.633637670485	1.595615560398
N	1.889500305789	8.893809105833	-0.555378800592
N	-4.066345218031	2.179867747206	-1.057235916180
N	-3.938577429787	1.922798714927	1.094121064531

8

B	-2.576675383679	-1.554136533442	-0.179925878324
B	-7.590087051690	-0.454077475235	2.982915440372
C	-2.736481659875	-2.709944496297	-1.167249226061
C	-5.556146176753	0.259386951929	1.629927067307
C	-1.785777243131	-2.654188647901	-4.060384002229
C	-3.859282392852	-1.198189021466	0.661288371596
C	-1.650303474502	-3.503800923034	-1.704864698779
C	-4.348328718873	0.112582324554	0.919807043663
C	-7.599460116380	1.421013595790	7.924755377938
C	-0.215504494056	-4.470203682837	-3.152607853451
C	-0.989225847019	-4.705739027958	0.404931299585
C	0.518862781687	-4.916316760597	-4.257857878926
C	-6.143688755423	0.977543309706	4.553818539728
C	-5.479474845972	-1.389316587339	5.142937705545
C	0.045701066783	-4.975626097496	-1.863570516705
C	-3.694973519266	1.331211387541	0.468684157093
C	-0.673397050325	-1.765765048291	-4.617775957469
C	1.512137071412	-5.867706966489	-4.021006236922
C	1.039520844788	-5.927762442544	-1.623570910295
C	-6.097290271498	3.472930682118	4.211377627889
C	1.770208247659	-6.365983425788	-2.728074288744
C	-6.657189531481	4.415188084796	5.275747778190
C	-2.414037038114	1.556872518854	0.083433277105
C	-1.237833931602	-0.738595977972	-0.012734146582
C	-5.043666011514	4.120311374436	3.309722516157
C	-1.235894188431	0.691251851590	0.027956775874
C	-4.157603325379	0.677667137167	5.584516465341
C	0.022834493499	-1.375245688518	-0.015221745859
C	-4.376077365705	2.063062848498	5.463655100683
C	-3.013036249424	0.156752554452	6.191683580897
C	0.000000000000	1.383357139575	0.000000000049
C	1.230757823428	-0.679690174642	0.003734594573
C	-3.451626146814	2.979414470305	5.976105310094
C	1.220135803106	0.718922849967	-0.003735478056
C	-2.087311733617	1.075282586121	6.691454545084

C	-2.306424183623	2.461558667658	6.586960162720
C	-7.429750590157	0.701452576004	3.970462165913
C	-4.610922131314	-2.267610488300	1.172565468469
C	-8.378260715702	0.644908218868	6.864195350888
C	-6.307720408532	-0.810019928196	2.141299966593
C	-8.515605441409	1.495107112927	4.509023150951
C	-5.818745095999	-2.120805698234	1.882695819195
C	-2.563788553763	-3.430482722417	-5.121378018428
C	-9.949365125870	2.461049411678	5.958076270309
C	-9.178139204891	2.697705308760	2.400060587614
C	-10.682958872353	2.906790418111	7.063986424871
C	-4.022229362889	-2.985937171795	-1.751352189355
C	-4.685790204463	-0.618964619208	-2.340706950327
C	-10.211513523719	2.966846201952	4.669374950337
C	-6.472067263582	-3.339440910285	2.333839612088
C	-9.490110748159	-0.243741931909	7.422281146579
C	-11.676437712137	3.858208984622	6.828138742004
C	-11.205548573597	3.919017608021	4.430377837691
C	-4.069584997535	-5.481279656794	-1.408439619147
C	-11.935455568277	4.356872962838	5.535540263868
C	-3.509444706961	-6.423993564140	-2.472278198435
C	-7.752899747090	-3.565094428765	2.719446009355
C	-8.929005185118	-1.269599944681	2.816146363177
C	-5.123922616716	-6.128081717221	-0.507194252204
C	-8.931000422289	-2.699447886431	2.775350515017
C	-6.007639982474	-2.685746895147	-2.783274764083
C	-10.189656024012	-0.632919990863	2.819142423927
C	-5.789522864612	-4.071175671991	-2.662172297733
C	-7.151704111044	-2.164650776203	-3.391232219139
C	-10.166899336187	-3.391523418427	2.803679238757
C	-11.397606020327	-1.328446691197	2.800555255113
C	-6.713800165426	-4.987380134449	-3.175203717629
C	-11.387018167556	-2.727055991577	2.807898596891
C	-8.077252263652	-3.083030196916	-3.891605609556
C	-7.858473069176	-4.469339850329	-3.786892744513
H	-2.484164654212	-2.002413926501	-3.524220770201
H	-5.916946094511	1.275779642342	1.802269483133
H	-6.537167864857	-1.616676070190	4.967306695386
H	-1.182150223919	-5.783978998894	0.504852061745
H	-8.244857890890	2.112530797468	8.486296819093
H	-1.822586534845	-4.136192973256	0.830214457219
H	0.331796969965	-4.547376424971	-5.264802903552
H	-4.336946467163	2.218083257364	0.498743676223
H	-7.158752417628	0.711814849867	8.641027633801
H	-6.914624420167	3.155709678474	3.558353416156
H	-1.121616255687	-1.000292805969	-5.268645770858
H	-7.441608222467	3.925464874153	5.869820191160
H	-4.877612615470	-1.926273716435	4.398425585009
H	0.057531604849	-2.329045383049	-5.214808457897
H	-6.783671730423	2.000559262967	7.468027129102
H	-5.198390825863	-1.717691933694	6.153575291654
H	-7.096203233233	5.298442845443	4.788153390222
H	2.103199063091	-6.235677718105	-4.862396190555
H	-0.063774897804	-4.454320234248	0.942805714421
H	1.232847784705	-6.311033223378	-0.620786185042
H	-0.141635122973	-1.256694466311	-3.799742287154
H	2.556071416394	-7.111407242281	-2.588789552196
H	-2.185996930898	2.602219018300	-0.149937960438
H	-5.519823444055	4.928211033127	2.734090998258
H	0.076377212421	-2.459417598247	-0.027059333725
H	-4.633495440795	3.385398661610	2.601621162005
H	-5.872381712391	4.763021333068	5.964174971845
H	-4.211938996334	4.559362180796	3.877068230899
H	-2.849668038414	-0.918500998238	6.274539983267
H	-0.015184266866	2.476478916357	-0.000674227245
H	-3.608359246312	4.054996853123	5.915716121569
H	2.176089156676	-1.228176458084	0.007861034442
H	2.155186415149	1.284562672153	-0.010203235172
H	-1.179175081143	0.710304565918	7.176099086916
H	-1.565730962031	3.152861348869	6.994795922162
H	-7.680249057574	-0.006684324929	6.327301531398
H	-4.250138703603	-3.284007372294	1.000207837774
H	-3.628187690827	-0.391740722728	-2.164336267176
H	-8.985474946039	3.776012586114	2.300373156650
H	-1.918031717008	-4.122275510933	-5.682163571232
H	-8.344949578559	2.128442001359	1.974068129670
H	-10.495163723239	2.537539718627	8.070679375609
H	-5.830141388154	-4.226334830949	2.303516646678
H	-3.003775446255	-2.721433302634	-5.838241861173
H	-3.252511448086	-5.164206669175	-0.755030980175
H	-9.041298704068	-1.009322896122	8.072616088217
H	-2.724465867721	-5.934732750502	-3.065973268135
H	-5.288103315202	-0.081981308344	-1.596568330162
H	-10.220608081412	0.319333734999	8.020031613910
H	-3.380062681844	-4.009756000836	-4.665171717821
H	-4.966147973559	-0.290482527770	-3.351511383535
H	-3.071099380554	-7.307333862952	-1.984238834488
H	-12.266911048478	4.225893955427	7.670064190942
H	-10.103881864472	2.446300911639	1.862681013965
H	-11.399619250490	4.302569198303	3.427844751791
H	-10.022490201457	-0.752675948619	6.604562781883
H	-12.721447326080	5.102310369267	5.397051751639
H	-7.980900209911	-4.610454245480	2.952795893388
H	-4.648344795421	-6.935964065918	0.068942575668
H	-10.243153136550	0.451250566588	2.831104864624
H	-5.534263055127	-5.392838938974	0.200458657097
H	-4.293985350328	-6.771631900293	-3.161109806974
H	-5.955461575124	-6.567046624526	-1.074872818268
H	-7.314812621377	-1.089370036731	-3.474249877321
H	-10.151744057366	-4.484645881468	2.804267621876
H	-6.557311936593	-6.062985868472	-3.114650751575
H	-12.342926969191	-0.779937342514	2.796817253347
H	-12.322081172639	-3.292671358287	2.814648696605
H	-8.984986572104	-2.717904738756	-4.376892284034
H	-8.599013046677	-5.160524366154	-4.195207084346
N	-1.261780428844	-3.555429007921	-3.010543151672
N	-0.859581198837	-4.365036441200	-1.002992634425
N	-5.268003894475	0.043097069465	5.037935016469
N	-5.597711651721	2.213659079408	4.801836764063
N	-8.903174879043	1.546344160553	5.814983092030
N	-9.306826394789	2.356536835155	3.807963831526
N	-4.897510926022	-2.051358061441	-2.235945541768
N	-4.568387281975	-4.221971719251	-1.999473533735

9

B	1.27900947787301	-0.83624244812925	2.56678433830913
B	-1.2778772362295	0.82954078082041	-2.56365377349877
C	0.01777535296340	-1.60886751039247	7.66339455374156
C	0.12795479632811	0.86343221842031	1.06167001730501
C	0.08546234609718	-0.47846288929489	6.83515572520800
C	0.44462171914547	-0.73972177486490	3.83973213130259
C	0.52169080111237	-1.31980893591001	0.10259120302894
C	0.62778530584400	-0.43537522244672	1.20337461953733
C	0.7049730039273	-0.64582448028603	-7.12132110094742
C	0.85699700628687	1.62653160801900	-8.74520791486545
C	1.05005514477014	-2.67672956660366	0.11722126782046
C	1.31764721000642	3.21659875079282	-5.17551571947492
C	1.79519713263949	4.14939380797241	-6.09510763099245
C	1.5738941054007	-0.62522449952996	-8.21254189470917
C	1.65275576510573	0.51097580118698	-9.02357134021931
C	1.4540583165786	-1.94494293733228	-4.05085991383447
C	1.72805934579105	2.92735390026323	-1.92471001893874
C	1.93608309136092	1.96001436336535	-5.04919660149865
C	1.95312805829452	1.21153710466378	5.47135224869952
C	1.93632037689968	1.56337917246345	-2.18677587526731
C	2.09851774808361	-3.17333212345044	0.82011857454711
C	2.24794156311540	3.50703152901999	-0.76694787793392
C	2.16227828917444	1.95998793470402	4.30333824505636
C	2.1918888869816	-0.76211506339226	-3.91005452110426

C	2.30309074925872	1.76681610235252	6.71506999005028
C	2.10391994143970	-3.17846475673909	-4.13666501819756
C	2.44532182218518	-2.94811680020670	5.12706184338176
C	2.50474311689120	-1.69280788974828	5.74844656844156
C	2.88626475068107	3.83282428802775	-6.91065173909291
C	2.77707762457780	-1.32437461273508	2.47548207019115
C	2.66317728232065	0.78940746002522	-1.26803975176211
C	2.72590412429721	3.23582196646184	4.37253778610082
C	2.97922607992934	2.73394124767463	0.14001732318616
C	3.01591976559944	1.63992550962287	-5.88291695335841
C	2.87155041133081	3.04060524738441	6.78055829990280
C	3.05045899726882	-2.49414464448680	1.70489006460109
C	3.08492490895972	3.77700674442239	5.61059494709422
C	3.18094888271542	1.37493528509024	-0.11171348742754
C	3.48402962984609	2.57462225424952	-6.81058056856507
C	3.42786644972268	-3.90360148483056	5.38928540797015
C	3.55015019072447	-1.40082654050761	6.63745403658163
C	3.4988359972068	-3.23778862024607	-4.07905060733462
C	3.5961028421870	-0.82787692162930	-3.84098161207519
C	3.85567420621497	-0.73219510711190	3.15910450244477
C	4.47082884974931	-3.61172854948687	6.27378019947649
C	4.52909695902341	-2.36137553313311	6.89818155372232
C	4.34228726654012	-3.06747389091761	1.75971332907131
C	4.2427995293045	-2.06125322996503	-3.93021964387512
C	5.13912994832376	-1.27602654807185	3.15353567547120
C	5.37992480084603	-2.47051609211627	2.46700708152649
C	-0.0136505931312	1.61357769266991	-7.65472098013511
C	-0.1240032040629	-0.86947292242608	-1.05947414863377
C	-0.0858969333784	0.47890815782934	-6.83272626912328
C	-0.4448318335552	0.73438643559169	-3.83762302852419
C	-0.5172120459877	1.31396787928790	-0.10054954493970
C	-0.6243142482985	0.42923890896262	-1.20105714042561
C	-0.7060002769916	0.64663868348043	7.12069291697251
C	-0.8508364153982	-1.61826602394697	8.75554477793560
C	-1.0447631356755	2.67116742424273	-0.11511280530705
C	-1.3271169081400	-3.21551235519917	5.17604067448811
C	-1.8099560638677	-4.14762512543494	6.09354961956399
C	-1.5724275085555	0.62984197019002	8.21394661928600
C	-1.6469435302546	-0.50226081679293	9.03106704772798
C	-1.4460729529322	1.94604206154394	4.04437594098180
C	-1.7341607956843	-2.92820775301096	1.92687107498885
C	-1.9425491428488	-1.95765010216519	5.04770414896816
C	-1.9530011283276	-1.21490324661576	-5.47246149438256
C	-1.9359942193143	-1.56266220826967	2.18572124615393
C	-2.0942966167636	3.16760006107234	-0.81642139584563
C	-2.2539223162172	-3.50737048269695	0.76879167030728
C	-2.1625934715017	-1.96534032724182	-4.30579070537216
C	-2.1875578125610	0.76527609507719	3.90550889248320
C	-2.3061558508217	-1.76652185251175	-6.71691066046672
C	-2.0918902332890	3.18205407198330	4.12445769849740
C	-2.4471418245847	2.94411048107244	-5.12052542741900
C	-2.5058280441704	1.69011451813898	-5.74459940378412
C	-2.9037413134141	-3.82931411331660	6.90473637976372
C	-2.7759013980179	1.31780715910057	-2.46963516338486
C	-2.6570827438915	-0.78683780095577	1.26402021802708
C	-2.7285522303225	-3.24003371999301	-4.37712555770903
C	-2.9792564137558	-2.73235299663486	-0.14133053673007
C	-3.0256279512979	-1.63610245233531	5.87665466977660
C	-2.8765707540047	-3.03932345110688	-6.78454515368334
C	-3.0479275638703	2.48784381712418	-1.69889474526882
C	-3.0904474936017	-3.77765814825006	-5.61589550060936
C	-3.1745484924955	-1.37181924716271	0.10726865429127
C	-3.4992026559353	-2.57023888395712	6.80211899475664
C	-3.4285807294134	3.90067741836826	-5.38295492843068
C	-3.5494209340408	1.40056307848551	-6.63652347196918
C	-3.4864628813794	3.24594241447082	4.06350640582853
C	-3.5913297210733	0.83570181476875	3.83204457305705
C	-3.8559434503419	0.72521759151827	-3.15061728132393
C	-4.4699532099547	3.61106065320527	-6.27004480493607
C	-4.5273657832599	2.36211619595688	-6.89731109334567
C	-4.3401569970301	3.06054219793605	-1.75053096192000
C	-4.2340148111029	2.07158485771648	3.91554574327662
C	-5.1395853903547	1.26857503851764	-3.14208706682855
C	-5.3793165167905	2.46293206499292	-2.45499759503741
H	0.21056229656734	1.56107536957408	1.89556206645438
H	0.44385026532572	3.46346518099661	-4.57330326600338
H	0.5809965545025	-3.35918429810389	-0.59875922608088
H	0.6360640229457	-2.48247737322032	7.45439230138410
H	0.3673264018354	-1.90699960958832	-4.09168143937592
H	0.6389648044014	-1.54461814282376	-6.50915135136605
H	0.9175334745086	2.51595910455565	-9.37586250344017
H	1.30484756226335	5.12180182797973	-6.18118647605489
H	1.15146341493196	3.54616022918557	-2.60997440883569
H	1.65220252886427	-3.16364921056330	4.41207022962798
H	1.5150634977593	-4.09203198360503	-4.24551817906248
H	1.87973308804064	1.55638693561395	3.33135647549395
H	2.07389154488314	4.56789092614149	-0.57311160086690
H	2.11732893243352	1.21665450006711	7.63909180620583
H	2.1874716509700	-1.50229205821889	-8.43054334565149
H	2.36513508658113	-4.21258186053836	0.59955480180147
H	2.33286380710277	0.52567508304317	-9.87864346958597
H	3.25700040573001	4.55972500949681	-7.63719665005762
H	2.8099607950067	-0.27626626914027	-1.43134091886623
H	2.87884580309893	3.80739632461936	3.45434497613533
H	3.38719078105199	3.18887651115823	1.04596738885882
H	3.13972031486011	3.46197737557540	7.75210518149786
H	3.38534228609302	-4.87357864394114	4.88907270356616
H	3.48292681197422	0.65731377621939	-5.83805192061050
H	3.61982923728152	-0.42399502265747	7.11630379583737
H	3.52418341653128	4.77593538355898	5.66518513411590
H	3.69628472714408	0.17590459450556	3.73733182599401
H	3.72422837911573	0.75480089400031	0.60376351884039
H	4.31775081077183	2.30916967759393	-7.46448416329229
H	4.0095077930225	-4.20153827396226	-4.14501510591436
H	4.18817926810993	0.07816688428011	-3.70100347978154
H	4.52943191081345	-3.98970712036657	1.20272178831555
H	5.24459513953989	-4.35742911322099	6.47180904549723
H	5.34481003049546	-2.12742301104960	7.58617225910184
H	5.3329701154257	-2.10449573414940	-3.87487025595523
H	5.94157149345881	-0.78340655649770	3.70802924290629
H	6.37210674313491	-2.92853895220814	2.47837453216587
H	-0.2072416212433	-1.56737613741931	-1.89309060213328
H	-0.4508277415165	-3.46354115683867	4.57785842818435
H	-0.5752436367504	3.35336140864484	0.60096199468919
H	-0.6325098448677	2.48641508014129	-7.44419066610151
H	-0.3595732372070	1.90452557569797	4.08797266669840
H	-0.6439323443127	1.54204285537144	6.50324720375723
H	-0.9071866039081	-2.50410015880903	9.39162674176285
H	-1.3215407379844	-5.12083621202867	6.18151875610355
H	-1.1616538799627	-3.54842177896512	2.61436525650952
H	-1.6555276551768	3.15775516496296	-4.40334551683395
H	-1.5001661130740	4.09391535450080	4.23198132088509
H	-1.8769216432481	-1.56491337563771	-3.33340788472783
H	-2.0852794966583	-4.56959075927241	0.57766668188625
H	-2.1192476276380	-1.21527336612555	-7.64005135066173
H	-2.1855383460954	1.50765165122745	8.43026836787872
H	-2.3600374196054	4.20720158663440	-0.59644843869855
H	-2.3265393127326	-0.51473385048631	9.88658009229597
H	-3.2786126096636	-4.55564370744028	7.62972170715828
H	-2.7979321810914	0.28007324912039	1.42446460716530
H	-2.8834473957155	-3.81249361428655	-3.45980705321996
H	-3.3860812570678	-3.18667224951647	-1.04814307703367
H	-3.1476951774776	-3.45762429828288	-7.75660093769932
H	-3.3866226242437	4.86960274917535	-4.88065081849384
H	-3.4905321524313	-0.65259688114885	5.83044537680974
H	-3.6183798132974	0.42493360175187	-7.11783574991425
H	-3.5308158266406	-4.77600161326843	-5.67222972256098
H	-3.6976836059561	-0.18290391043686	-3.72900148079553
H	-3.7141048895231	-0.75050871498986	-0.61002744195385
H	-4.3349657474566	-2.30337622271591	7.45283136400958

H	-3.9939353929061	4.21173838257345	4.12400656214253
H	-4.1860725952033	-0.06855560759901	3.69192933932104
H	-4.5263407042691	3.98274842061608	-1.19316851127912
H	-5.2426204544215	4.35775892307687	-6.46860648641487
H	-5.3420757756851	2.12975363406374	-7.58702811774149
H	-5.3239507638224	2.11826745117619	3.85839719071880
H	-5.9432080735378	0.77539491494998	-3.69436778342254
H	-6.3718494900344	2.92027817788356	-2.46366137652242
P	1.20856104799412	-0.46206313213158	5.40207115193054
P	1.31769218373309	0.82757457041579	-3.74926677942750
P	-1.2093936223632	0.45903850283167	-5.40003730880380
P	-1.3180702065442	-0.82740497844481	3.74867984114106

References

1. Harris, R. K.; Becker, E. D.; Menezes, S. M. C. d.; Goodfellow, R.; Granger, P., NMR nomenclature. Nuclear spin properties and conventions for chemical shifts(IUPAC Recommendations 2001). *Pure Appl. Chem.* **2001,** *73*, 1795-1818
2. Caruso Jr., A.; Siegler, M. A.; Tovar, J. D., Synthesis of Functionalizable Boron-Containing π-Electron Materials that Incorporate Formally Aromatic Fused Borepin Rings. *Angew. Chem. Int. Ed.* **2010,** *49*, 4213-4217
3. Hollister, K. K.; Molino, A.; Jones, N.; Le, V. V.; Dickie, D. A.; Cafiso, D. S.; Wilson, D. J. D.; Gilliard, R. J., Jr., Unlocking Biradical Character in Diborepins. *J. Am. Chem. Soc.* **2024,** *146*, 6506-6515
4. Jazzar, R.; Dewhurst, R. D.; Bourg, J.-B.; Donnadieu, B.; Canac, Y.; Bertrand, G., Intramolecular "Hydroiminiumation" of Alkenes: Application to the Synthesis of Conjugate Acids of Cyclic Alkyl Amino Carbenes (CAACs). *Angew. Chem. Int. Ed.* **2007,** *46*, 2899-2902
5. Arduengo, A. J.; Krafczyk, R.; Schmutzler, R.; Craig, H. A.; Goerlich, J. R.; Marshall, W. J.; Unverzagt, M., Imidazolylidenes, imidazolinylidenes and imidazolidines. *Tetrahedron* **1999,** *55*, 14523-14534
6. Kuhn, N.; Kratz, T., Synthesis of Imidazol-2-ylidenes by Reduction of Imidazole-2(3H)-thiones. *Synthesis* **1993,** *1993*, 561-562
7. Chen, W.-C.; Hsu, Y.-C.; Lee, C.-Y.; Yap, G. P. A.; Ong, T.-G., Synthetic Modification of Acyclic Bent Allenes (Carbodicarbenes) and Further Studies on Their Structural Implications and Reactivities. *Organometallics* **2013,** *32*, 2435-2442
8. Zybill, C.; Mueller, G., Mononuclear complexes of copper(I) and silver(I) featuring the metals exclusively bound to carbon. Synthesis and structure of (.eta.5-pentamethylcyclopentadienyl)[(triphenylphosphonio)(triphenylphosphoranylidene)methyl]copper(I). *Organometallics* **1987,** *6*, 2489-2494
9. Pranckevicius, C.; Iovan, D. A.; Stephan, D. W., Three and four coordinate Fe carbodiphosphorane complexes. *Dalton Trans.* **2016,** *45*, 16820-16825
10. Bruker (**2020**). SAINT, Bruker-AXS Inc., Madison, Wisconsin, USA.
11. Krause, L.; Herbst-Irmer, R.; Sheldrick, G.; Stalke, D., Comparison of silver and molybdenum microfocus X-ray sources for single-crystal structure determination. *J. Appl. Crystallogr.* **2015,** *48*,
12. Sheldrick, G., SHELXT - Integrated space-group and crystal-structure determination. *Acta Cryst. A* **2015,** *71*, 3-8
13. Sheldrick, G. M., Crystal structure refinement with SHELXL. *Acta Crystallogr C Struct Chem* **2015,** *71*, 3-8
14. Müller, P., Practical suggestions for better crystal structures. *Crystallography Reviews* **2009,** *15*, 57-83
15. Dolomanov, O. V.; Bourhis, L. J.; Gildea, R. J.; Howard, J. A. K.; Puschmann, H., OLEX2: a complete structure solution, refinement and analysis program. *J. Appl. Crystallogr.* **2009,** *42*, 339-341
16. Neese, F., Software update: The ORCA program system—Version 5.0. *WIREs Comput Mol Sci* **2022,** *12*, e1606
17. Weigend, F.; Ahlrichs, R., Balanced basis sets of split valence, triple zeta valence and quadruple zeta valence quality for H to Rn: Design and assessment of accuracy. *Phys. Chem. Chem. Phys.* **2005,** *7*, 3297-3305
18. Grimme, S.; Antony, J.; Ehrlich, S.; Krieg, H., A consistent and accurate ab initio parametrization of density functional dispersion correction (DFT-D) for the 94 elements H-Pu. *J. Chem. Phys.* **2010,** *132*,
19. Barone, V.; Cossi, M., Quantum Calculation of Molecular Energies and Energy Gradients in Solution by a Conductor Solvent Model. *J. Phys. Chem.* **1998,** *102*, 1995-2001

20. te Velde, G.; Bickelhaupt, F. M.; Baerends, E. J.; Fonseca Guerra, C.; van Gisbergen, S. J. A.; Snijders, J. G.; Ziegler, T., Chemistry with ADF. *J. Comput. Chem.* **2001,** *22*, 931-967
21. Geuenich, D.; Hess, K.; Köhler, F.; Herges, R., Anisotropy of the Induced Current Density (ACID), a General Method To Quantify and Visualize Electronic Delocalization. *Chem. Rev.* **2005,** *105*, 3758-3772
22. Yanai, T.; Tew, D. P.; Handy, N. C., A new hybrid exchange–correlation functional using the Coulomb-attenuating method (CAM-B3LYP). *Chem. Phys. Lett.* **2004,** *393*, 51-57
